# Photoinduced Autonomous Nonequilibrium Operation of a Molecular Shuttle by Combined Isomerization and Proton Transfer Through a Catalytic Pathway

**DOI:** 10.1021/jacs.1c13537

**Published:** 2022-05-16

**Authors:** Federico Nicoli, Massimiliano Curcio, Marina Tranfić Bakić, Erica Paltrinieri, Serena Silvi, Massimo Baroncini, Alberto Credi

**Affiliations:** † CLAN-Center for Light Activated Nanostructures, ISOF-CNR, Via Gobetti 101, 40129 Bologna, Italy; ‡ Dipartimento di Chimica Industriale “Toso Montanari”, Università di Bologna, Viale Risorgimento 4, 40136 Bologna, Italy; § Dipartimento di Chimica “G. Ciamician”, Università di Bologna, Via Selmi 2, 40126 Bologna, Italy; ∥ Dipartimento di Scienze e Tecnologie Agro-Alimentari, Università di Bologna, Viale Fanin 44, 40127 Bologna, Italy

## Abstract

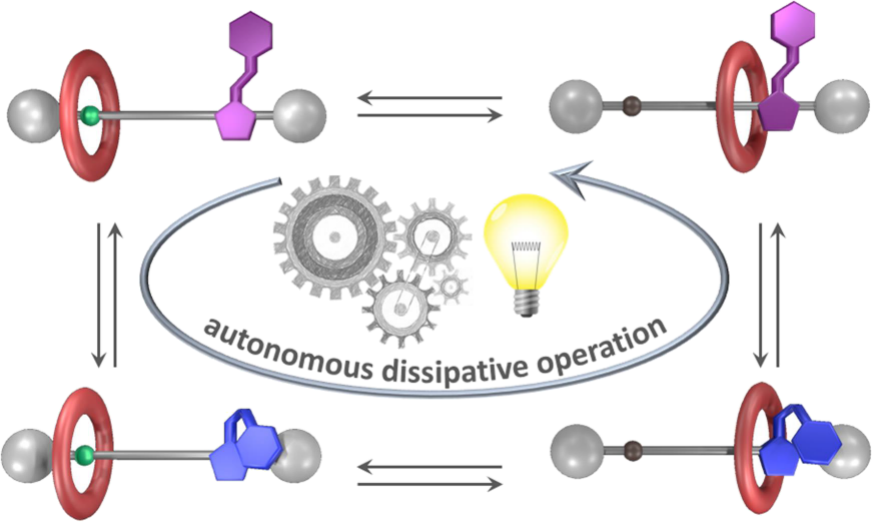

We describe a [2]rotaxane whose recognition sites for the ring are a dibenzylammonium moiety, endowed with acidic and H-bonding donor properties, and an imidazolium center bearing a photoactive phenylazo substituent. Light irradiation of this compound triggers a network of *E*/*Z* isomerization and proton transfer reactions that enable autonomous and reversible ring shuttling away from equilibrium.

## Introduction

The construction of nanoscale machines^1−3^ activated by light as a clean and highly controllable energy source^4,5^ can enable ground-breaking applications in technology and medicine.^6−14^ Although artificial molecular machines that exploit light energy autonomously—i.e., able to repeatedly execute their function once triggered by a stimulus, without additional external intervention^15^—to generate continuous motion are appealing,^1−8^ also for energy conversion purposes,^16^ their development is highly challenging, and only a few classes of systems have been reported to date.^17−21^ Here, we describe a [2]rotaxane in which reversible and continuous ring shuttling between the extremities of an axle occurs as a result of the entanglement of photoinduced isomerization and proton transfer processes, which is made possible by the mechanical interlocking of the molecular components.

**Figure 1:**
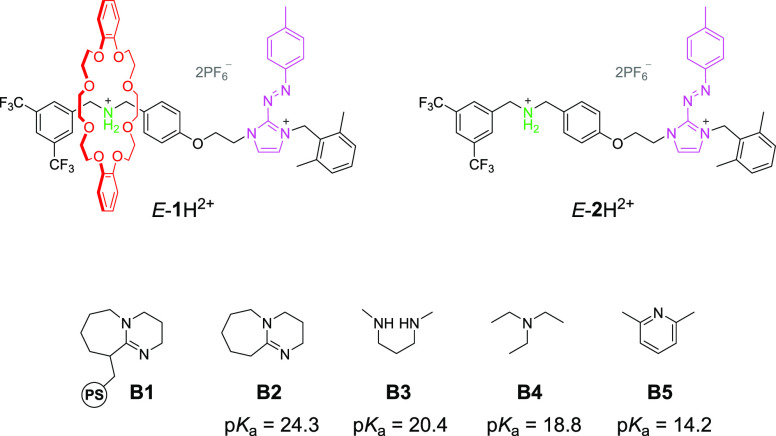
Structures of rotaxane *E*-**1**H^2+^, thread *E*-**2**H^2+^, and bases **B1**-**B5** with their respective p*K*_a_ values in acetonitrile.^33^

Rotaxane *E*-**1**H^2+^ (Figure 1) consists of a dibenzo-24-crown-8 (DB24C8) ring and an axle containing a pH-responsive dibenzylammonium site and a photosensitive arylazoimidazolium unit. Imidazolium cations were employed in rotaxane chemistry as both hydrogen bond donor^22−25^ and ionic^26^ stations, while azoimidazolium compounds found applications in light-effected ionic liquids,^27,28^ high-energy materials,^29^ and photoresponsive drugs.^30^ Nonetheless, the potential of arylazoimidazolium as the active unit for molecular machines is to date unexplored.

## Results and Discussion

**Figure 2:**
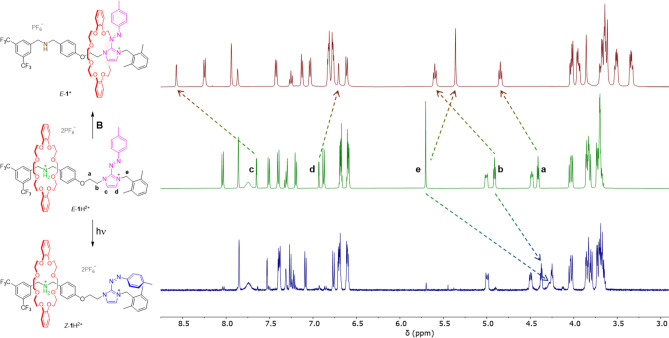
Partial ^1^H NMR spectra (500 MHz, acetonitrile-*d*_3_, 298 K) of *E*-**1**H^2+^ (center), *E*-**1**^+^ (top), and *Z*-**1**H^2+^ (bottom).

**Scheme 1:**
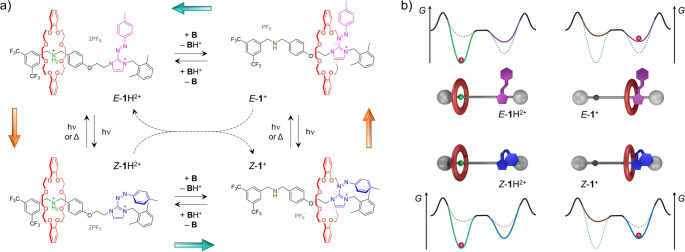
(a) Closed Network of Acid–Base (Horizontal) and Photochemical (Vertical) Reactions Connecting the Four States That Can Be Obtained from Rotaxane *E*-**1**H^**2**+^ and (b) Qualitative Energy Diagrams of the Corresponding Rotaxane States Indicating the Changes in Energy Induced by the Deprotonation/Protonation and Isomerization/Back-Isomerization Processes^a^ The inner dashed arrows indicate the bimolecular proton transfer between *Z*-**1**H^**2**+^ and *E*-**1**^+^. The outer colored arrows indicate the preferential anticlockwise direction during the out-of-equilibrium cycling. The red sphere indicates the preferential position of the macrocycle along the axle

Rotaxane *E*-**1**H^2+^ and its axle *E*-**2**H^2+^ (Figure 1) were synthesized according to the procedures reported in the Supporting Information and investigated by means of NMR spectroscopy and UV–visible spectrophotometry in acetonitrile. The ^1^H NMR spectrum of *E*-**1**H^2+^ (Figure 2, center) confirmed that the DB24C8 ring encircles the ammonium station. Treatment of *E*-**1**H^2+^ with a base **B** (Figure 1) led to the immediate (on the time scale of the experiment, that is, a few minutes) formation of the deprotonated rotaxane *E*-**1**^+^ in which the ring encircles the azoimidazolium station (Scheme 1a). The ring shuttling is supported by the deshielding observed for the nuclei of the ethylene bridge **a** and **b** and the imidazolium proton **c**, as well as by the shielding of the nuclei **d** and **e** (Figure 2, top). The p*K*_a_ of the ammonium center of *E*-**1**H^2+^, determined by titration with the base **B3**, is 20.74. By comparison, the free axle *E*-**2**H^2+^ exhibits a p*K*_a_ of 14.15, indicating that the acidity of the ammonium center of the rotaxane is significantly lowered because of the interaction with the surrounding ring, in line with literature reports.^31,32^

Upon irradiation of *E*-**1**H^2+^ at 365 nm, several resonances underwent a pronounced shielding (Figure 2, bottom), consistent with the isomerization to *Z*-**1**H^2+^ (Scheme 1). The NMR spectrum indicated that in *Z*-**1**H^2+^, the DB24C8 ring remains on the ammonium station. Notably, no photoisomerization was observed for the deprotonated rotaxane *E*-**1**^+^ in analogous experimental conditions or at lower temperatures. In contrast, the deprotonated axle *E*-**2**^+^ was photoconverted to the corresponding *Z*-form, although a lower temperature was required to enable a substantial accumulation of the latter (Figure S42). The p*K*_a_ of *Z*-**2**H^2+^, determined by titration with the base **B5** under photostationary conditions, resulted to be 14.14 (Figure S52); as this value is practically identical to that of *E*-**2**H^2+^, isomerization of the azoimidazolium unit does not affect the acidic properties of the ammonium when there is no ring encircling the axle.

In an attempt to obtain *Z*-**1**^+^ by deprotonation of *Z*-**1**H^2+^, a solution of *E*-**1**H^2+^ under continuous irradiation (PSS, 90:10 *Z/E* ratio) was reacted with bases **B2**, **B3**, and **B4** (Figures S46–S48). Surprisingly, in all cases, regardless of the strength of the base used, the addition of a substoichiometric amount of base (0.10 equivalents) caused the complete disappearance of the resonances of *Z*-**1**H^2+^, with formation of a mixture of *E*-isomers only. To further investigate this unexpected phenomenon, *E*-**1**^+^ was reacted with substoichiometric amounts of trifluoroacetic acid (to afford *E*-**1**H^2+^) under constant 365 nm irradiation. Despite the ability of *E*-**1**H^2+^ to photoisomerize (vide supra), the sole product observed during the titration was *E*-**1**H^2+^ itself (Figure S49); *Z*-**1**H^2+^ started to appear only upon addition of 1 equivalent of acid, i.e., after complete protonation of the reactant. These results show that *Z*-**1**H^2+^—which can be efficiently produced by irradiation of *E*-**1**H^2+^—cannot exist in solution, even under continuous illumination, if the deprotonated rotaxane *E*-**1**^+^ is present, pointing to the occurrence of a secondary reaction pathway responsible for a fast *Z* → *E* transformation.

The unsuccessful observation of *Z*-**1**^+^ upon irradiation of the *E*-isomer could arise from (i) low *E* → *Z* and/or high *Z* → *E* photoisomerization efficiencies, (ii) a very fast *Z* → *E* thermal isomerization, or a combination of these factors. To study this problem, we resorted to the UV–visible spectroscopic analysis on dilute solutions.

Irradiation at 365 nm of 20 μM *E*-**1**H^2+^ at 298 K afforded a PSS with a *Z*/*E* composition of ∼70:30. Under these conditions, also *E*-**1**^+^ could be photoisomerized to its *Z*-form, with a *Z*/*E* ratio of ∼30:70 at the PSS (Figure S55). The molar absorption coefficients at 375 nm (wavelength of the maximum of the *E* isomer) are similar for each *E*-**1**H^2+^/*E*-**1**^+^ and *Z*-**1**H^2+^/*Z*-**1**^+^ pair of rotaxanes (Table 1), but the *Z*-isomers exhibit significantly lower values than the *E*-forms. Hence, the *E*–*Z* interconversion could be conveniently monitored by measuring the absorbance at 375 nm. The rate constant for *Z* → *E* thermal isomerization of *Z*-**1**^+^ resulted to be almost 20 times faster than that of *Z*-**1**H^2+^, which in turn is comparable with those of the free axles *Z*-**2**H^2+^ and *Z*-**2**^+^.

**Table 1:** Photochemical Properties, Compositions of the Photostationary State (PSS) and Rates of Back-Isomerization for the Rotaxane and the Free Thread Compounds (Acetonitrile, *c* = 2.0 × 10^–5^ M, 298 K)

compound	absorption		PSS	back-isomerization
	λ_max_/nm	*ε*_375_(*E*)/cm^–1^ M^–1^	*E*:*Z*/%	*k*_Δ_/s^–1^
**1**H^2+^	375	25,832	30:70	(3.97 ± 0.06) × 10^–4^
**1**^+^	375	24,647	69:31	(6.96 ± 0.02) × 10^–3^
**2**H^2+^	375	24,128	17:83	(2.70 ± 0.01) × 10^–4^
**2**^+^	375	23,040	28:72	(6.63 ± 0.02) × 10^–4^

Taken together, these observations indicate that the presence of DB24C8 around the photoresponsive station leads to an enhanced *Z* → *E* thermal isomerization and that the main reason for the inefficient photogeneration of *Z*-**1**^+^ is likely its rapid thermal decay.

With this information in hand, we proceeded to study the interplay between the azoimidazolium photoisomerization and the ammonium acid–base properties suggested by the NMR experiments.

We previously showed that, in rotaxanes similar to **1**H^2+^, the apparent p*K*_a_ of the ammonium center depends on the strength of noncovalent interactions between the ring and the secondary pH-insensitive station.^34^ We also demonstrated that the modulation of such interactions by electrochemical stimuli can be exploited to modify the ammonium acidity.^35^ Such a behavior can be explained with thermodynamic arguments and is enabled by the ability of the ring to shuttle (that is, exchange chemical information) between the stations.^24−26^ We thus hypothesize that in **1**H^2+^, owing to the presence of a photoreactive secondary station, the ammonium p*K*_a_ could be modulated with light.

First of all, an experiment analogous to the one described in the NMR section was performed: a PSS mixture of the two isomers of **1**H^2+^(*Z/E* ∼ 70:30) was treated with either base **B2** or **B3** under continuous irradiation (Figure S59). In both cases, the addition of 0.10 equivalents of base caused an increase in absorbance at 375 nm, indicating the conversion to *E*-forms, coherently with the NMR data. This result may be explained in terms of the fast back-isomerization of *Z*-**1**^+^ produced by deprotonation of *Z*-**1**H^2+^; the increase of absorbance, however, was slower and much larger than that expected on this basis, suggesting that the addition of a base triggers an alternative process.

**Figure 3:**
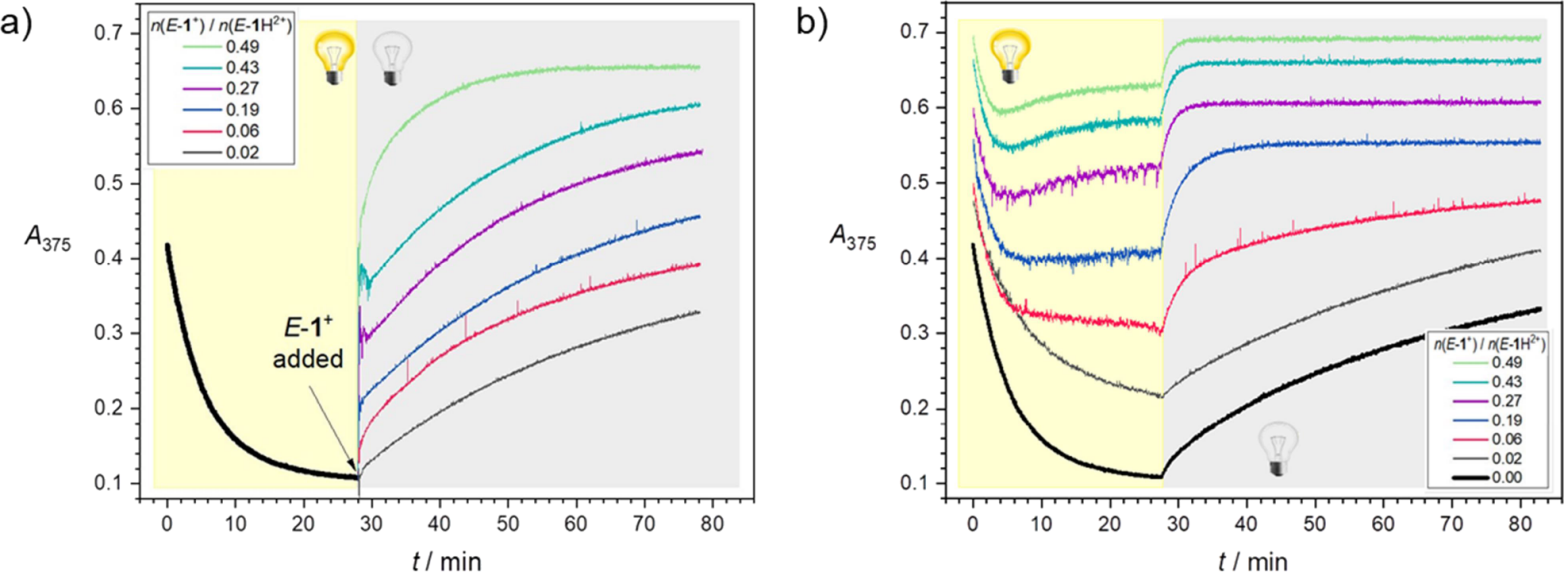
Time-dependent absorption changes at 375 nm for (a) *E*-**1**H^2+^ solutions irradiated at 365 nm until the PSS (yellow background) and upon termination of the irradiation and concomitant addition of different amounts of *E*-**1**^+^ (gray background); (b) solutions containing *E*-**1**H^2+^ and *E*-**1**^+^ in different proportions, upon irradiation at 365 nm light (yellow background), and subsequent rest in the dark (gray background). Conditions: 18 μM *E*-**1**H^2+^, acetonitrile, 298 K.

To investigate the relative acid–base properties of *E*-**1**H^2+^ and *Z*-**1**H^2+^ without increasing too much the complexity of the system, we used *E*-**1**^+^ as an “internal” base, thus avoiding the introduction of exogenous base/conjugated acid pairs. A sequential “light-on/light-off” experiment^36^ was performed: a solution of *E*-**1**H^2+^ was irradiated to the PSS, then the irradiation was stopped, and a known amount of *E*-**1**^+^ was added. This led to an increase in absorbance that was faster for larger amounts of *E*-**1**^+^ (Figure 3a). Should *E*-**1**H^2+^ be a stronger acid than *Z*-**1**H^2+^, no deprotonation reaction would occur: the addition of *E*-**1**^+^ would cause an immediate increase in absorbance, due to *E*-**1**^+^ added, followed by a slow increase related to the sole thermal back-isomerization of *Z*-**1**H^2+^. However, the time-dependent absorbance changes could be fitted only using a two-contribution kinetic model, providing two rate constants (Table S1). One of them, *k*_B_, correlates well with the thermal isomerization rate of *Z*-**1**H^2+^, while the other, *k*_A_, is an apparent pseudo-first order rate constant and increases proportionally to the amount of *E*-**1**^+^ added.

This likely results from *Z*-**1**H^2+^ being more acidic than *E*-**1**H^2+^ (Scheme 1b), so that *E*-**1**^+^ deprotonates *Z*-**1**H^2+^ to yield *E*-**1**H^2+^ and *Z*-**1**^+^ (eq 1 and Scheme 1a, dashed arrows).
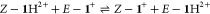
1

Such a scenario implies an immediate increase of absorbance at 375 nm, proportional to the amount of *E*-**1**^+^ added, followed by a slower increase arising from (i) the thermal back-isomerization of *Z*-**1**H^2+^ and (ii) the formation of *Z*-**1**^+^ and its subsequent thermal back-isomerization, in line with the experimental data.

It is important to note that *Z*-**1**^+^ produced in reaction 1 will isomerize back to the relatively more basic species *E*-**1**^+^ within seconds (Table 1). This triggers a catalytic pathway that can proceed until all the *Z*-**1**H^2+^ is converted into *E*-**1**H^2+^ (Figure S60a). This behavior is not exhibited by the axle **2**H^2+^, corroborating that the presence of the interlocked ring is essential to induce the p*K*_a_ difference between *E* and *Z* isomers (Figure S61).

Our results also confirm that rotaxane **1**H^2+^, in the presence of a base **B** of suitable strength, functions as a molecular shuttle in which the reversible translation of the ring occurs autonomously as a result of light-induced isomerization and proton-transfer processes (Scheme 1a). In fact, because of the different acidity of the *E* and *Z* isomers and the different PSS composition of the protonated and deprotonated compounds (Table 1), both the acid–base equilibria (horizontal processes) and the photoreactions (vertical processes) impart directionality to the cycle, making the anticlockwise pathway in Scheme 1a preferable with respect to the clockwise one. Obviously, light energy must be supplied to the system in order to connect together the acid–base equilibria and establish a closed reaction.^15^ Moreover, we have shown that the deprotonated rotaxane *E*-**1**^+^ can also play the role of **B**.

To gain direct observation of the states afforded by autonomous light-driven cycling, solutions containing different amounts of *E*-**1**H^2+^ and *E*-**1**^+^ were exposed to stationary irradiation at 365 nm and their absorbance at 375 nm was monitored over time (Figure 3b, yellow background). An initial absorbance decrease was detected in all cases because of the *E* → *Z* photoconversion. However, for solutions containing higher amounts of *E*-**1**^+^, the decrease was clearly followed by an increase (Figure 3b, *E*-**1**^+^/*E*-**1**H^2+^ ≥ 0.19). Such an inversion of trend, which was not observed for the *E*-**2**^+^/*E*-**2**H^2+^ system (Figure S62), implies that another process involving the transformation of *Z*-species takes over the isomerization and leads to higher absorbance values, owing to the accumulation of *E*-species. The experiments shown in Figure 3a indicate that this process is the deprotonation of *Z*-**1**H^2+^ by *E*-**1**^+^ to yield *Z*-**1**^+^, which rapidly back-isomerizes to *E*-**1**^+^, causing the observed increase in absorbance. The rate of the bimolecular deprotonation reaction depends on the concentrations of both *Z*-**1**H^2+^ and *E*-**1**^+^. Thus, the reaction is relatively slow at the beginning of the experiment, as the concentration of photogenerated *Z*-**1**H^2+^ is initially low, and becomes faster over time until it outperforms isomerization. Larger *E*-**1**^+^/*E*-**1**H^2+^ ratios cause faster deprotonation reactions and result in shorter times required to reach the inversion point (Figure 3b, yellow background). Eventually, the two reactions even out and a dissipative nonequilibrium state is reached under constant illumination at the absorbance plateau. In a closed system, this is a state sustained by an external energy input in which the detailed balance is not fulfilled.^37^ Thereby, the concentrations of the species involved in the cycle reach a stationary state at which the rates of all the reactions making up the cycle are equal and nonzero. The nonequilibrium nature of these states is confirmed by the full relaxation of the system in the dark to the global energy minimum consisting of *E*-**1**^+^ and *E*-**1**H^2+^ (Figure 3b, gray background).

In summary, we have reported a new strategy to achieve light-driven autonomous and reversible shuttling in a rotaxane. Though photoisomerization and proton-transfer processes have been frequently reported to operate molecular machines, their implementation for autonomous out-of-equilibrium operation has never been demonstrated.^38,39^ Here, the closed reaction network can be traveled in a preferential direction upon supplying light energy, enabled by the population of dissipative nonequilibrium states through the continuous interconversion of forms in which the ring encircles different portions of the axle. The present system is particularly interesting because the cycle directionality is supported by both energy ratchet (the energy stimulus changes the constants of the acid–base equilibria) and information ratchet (the position of the ring along the axle affects the *E–Z* interconversion) mechanisms. More generally, the rational design of dynamic chemical systems that can use light to operate away from thermal equilibrium is a stimulating challenge and a topic of high fundamental and applicative interest.

## Supporting Information Available

Methods, synthetic details, and spectroscopic and photochemical data (PDF)
