# Supplementary material for: Photoinduced Autonomous Nonequilibrium Operation of a Molecular Shuttle by Combined Isomerization and Proton Transfer Through a Catalytic Pathway
Source: J Am Chem Soc. 2022 May 16;144(23):10180–5. doi: 10.1021/jacs.1c13537 (PMC9204767; doi:10.1021/jacs.1c13537)
Supplement: Supplementary file 1 — ja1c13537_si_001.pdf [file ja1c13537_si_001.pdf]

# Photoinduced Autonomous Non-Equilibrium Operation of a Molecular Shuttle by Combined Isomerization and Proton Transfer Through a Catalytic Pathway

Federico Nicoli,[a,b,‡] Massimiliano Curcio,[a,b,‡] Marina Tranfić Bakić,[a,b,‡] Erica Paltrinieri,[a,b] Serena Silvi,[b,c] Massimo Baroncini,[b,d] Alberto Credi[a,b,*]

[a] Department of Industrial Chemistry "Toso Montanari", University of Bologna, Bologna, Italy.

[b] Center for Light Activated Nanostructures, National Research Council of Italy, Bologna, Italy.

[c] Department of Chemistry "Giacomo Ciamician", University of Bologna, Bologna, Italy.

[d] Department of Agricultural Science and Technology, University of Bologna, Bologna, Italy.

## SUPPORTING INFORMATION

# Table of contents

**1. Experimental details ........................................ 3**

NMR measurements ........................................ 3

NMR acidity measurements ........................................ 3

UV acidity determination experiments ........................................ 4

UV irradiation experiments ........................................ 4

**2. Synthetic procedures ........................................ 5**

**3. NMR data ........................................ 14**

**4. Spectrophotometric data ........................................ 44**

## 1. Experimental details

Solvents and reagents 3,5-bis-(trifluoromethyl)benzylamine, 4-hydroxybenzaldehyde, sodium borohydride, di-*tert*-butyl-dicarbonate, potassium carbonate, 1,2-dibromoethane, 4-methylaniline, sodium nitrite, hexafluorophosphoric acid, Imidazole, dibenzo-24-crown-8, 2-(Bromomethyl)-1,3-dimethylbenzene, ammonium hexafluorophosphate, trifluoroacetic acid, polystyrene-supported DBU, *N*,*N*-dimethylpropane-1,3-diamine (dMPDA) and lutidine were all used as supplied by Fluorochem, Sigma-Aldrich or VWR without further purification.

Flash column chromatography was performed using Sigma Aldrich Silica 40 (230-400 mesh size or 40-63 μm) as the stationary phase. Gel permeation chromatography was performed using Biorad Biobeads SX-1 as the stationary phase. Thin layer chromatography was performed on TLC Silica gel 60 F254 coated aluminium plates from Merck.

**NMR measurements**

$^{1}$H NMR spectra were recorded on an Agilent DD2 spectrometer operating at 500 MHz or a Varian Mercury spectrometer operating at 400 MHz; $^{13}$C NMR spectra were recorded on an Agilent DD2 spectrometer operating at 126 MHz or a Varian Mercury spectrometer operating at 101 MHz; $^{19}$F NMR spectra were recorded on an Agilent DD2 spectrometer operating at 470 MHz. Chemical shifts are quoted in ppm relative to tetramethylsilane ($SiMe_4$, δ = 0 ppm), using the residual solvent peak as a reference standard; all coupling constants (*J*) are expressed in Hertz (Hz). The samples were irradiated directly inside the thermostated NMR probe, using a 1 mm silica core optical fiber (Thorlabs) connected to a Prizmatix UHP-T-365-SR LED Illuminator (1.5 W, $\lambda_{max}$= 369 nm, FWHM, 15.56 nm) through a FCA-SMA adaptor. At its other end, the protective coating of the optical fiber was removed (about 6 cm) and the exposed fibre was sanded to enable the diffusion of light from the fibre core into the solution. The fibre prepared in this way was immersed directly into the thermostated solution (550 μL of 5 mM solution of the rotaxane/free thread) in the NMR tube. The obtained experimental data were processed using MestReNova software and OriginPro 2019. The photon flow at the end of the fibre was determined to be $4 \times 10^{-8}$ Einstein $s^{-1}$ using the ferrioxalate actinometry in its "microversion".[1]

**NMR acidity measurements**

Deprotonation experiments were carried out in acetonitrile-$d_3$ using heterogeneous [**B1**, polystyrene-supported DBU], and homogeneous [**B2**, DBU, p$K_a$ = 24.3; **B3**, dMPDA, p$K_a$ = 20.4; **B4**, Triethylamine, p$K_a$ = 18.8; **B5**, 2,6-Lutidine, p$K_a$ = 14.2] bases.[2] The spectra were acquired upon stepwise addition of the base directly into the NMR tube containing 550 μL of $c \approx 5$ mM solution of the sample. The experiments were performed in the dark or under continuous irradiation (using the set-up described in the previous paragraph) to assess the acidity of the *Z* moieties. Experimental data were processed using HypNMR from HYPERQUAD suite of programs[3] and OriginPro 2019. The error on the p$K_a$ values is estimated to be ±0.1 units, calculated as the average mean square root in the p$K_a$ values of the ammonium stations investigated.

**UV acidity determination experiments**

The acidity determinations were performed spectrophotometrically, using a double-beam Perkin-Elmer lambda 750 spectrophotometer at room temperature by a titration with a suitable organic base or acid. The spectra were acquired upon stepwise addition of the base directly into the 1 cm optical path length quartz cell, with scanning speed of 500 nm $min^{-1}$ with a 1 nm resolution. All titrations were performed at least in triplicate, and the obtained experimental data were processed using HypSPEC from HYPERQUAD suite of programs[3] and OriginPro 2019.

**UV irradiation experiments**

The isomerization experiments were performed using a diode array spectrophotometer Avantes StarLine AvaSpec-ULS2048CL-EVO-RS equipped with an optical fiber which enables the fast acquisition of UV/Vis spectra (ms) and concomitant *in situ* irradiation. About 3 ml of a solution ≈ 2 × $10^{-5}$ mol $dm^{-3}$ of the rotaxane/free thread in a 10mm pathlength quartz cuvette was continuously irradiated by a medium pressure Hg lamp (200 W) at room temperature. The irradiation wavelength was selected using interference filters and the intensity of the incident light was regulated with a pinhole of opportune diameter. The photon flow was determined by the "microversion" of the ferrioxalate actinometer[1] and resulted to be ≈ 1.5 × $10^{-9}$ Einstein $s^{-1}$. The acquisition of the spectra was prolonged after interrupting the irradiation of the sample, in order to study the thermal back-isomerization processes. The rate constants for thermal back-isomerization were obtained by fitting the back-isomerization spectral data at the wavelength of the maximum against time using a first-order kinetics (exponential) model in OriginPro 2019.

## 2. Synthetic procedures

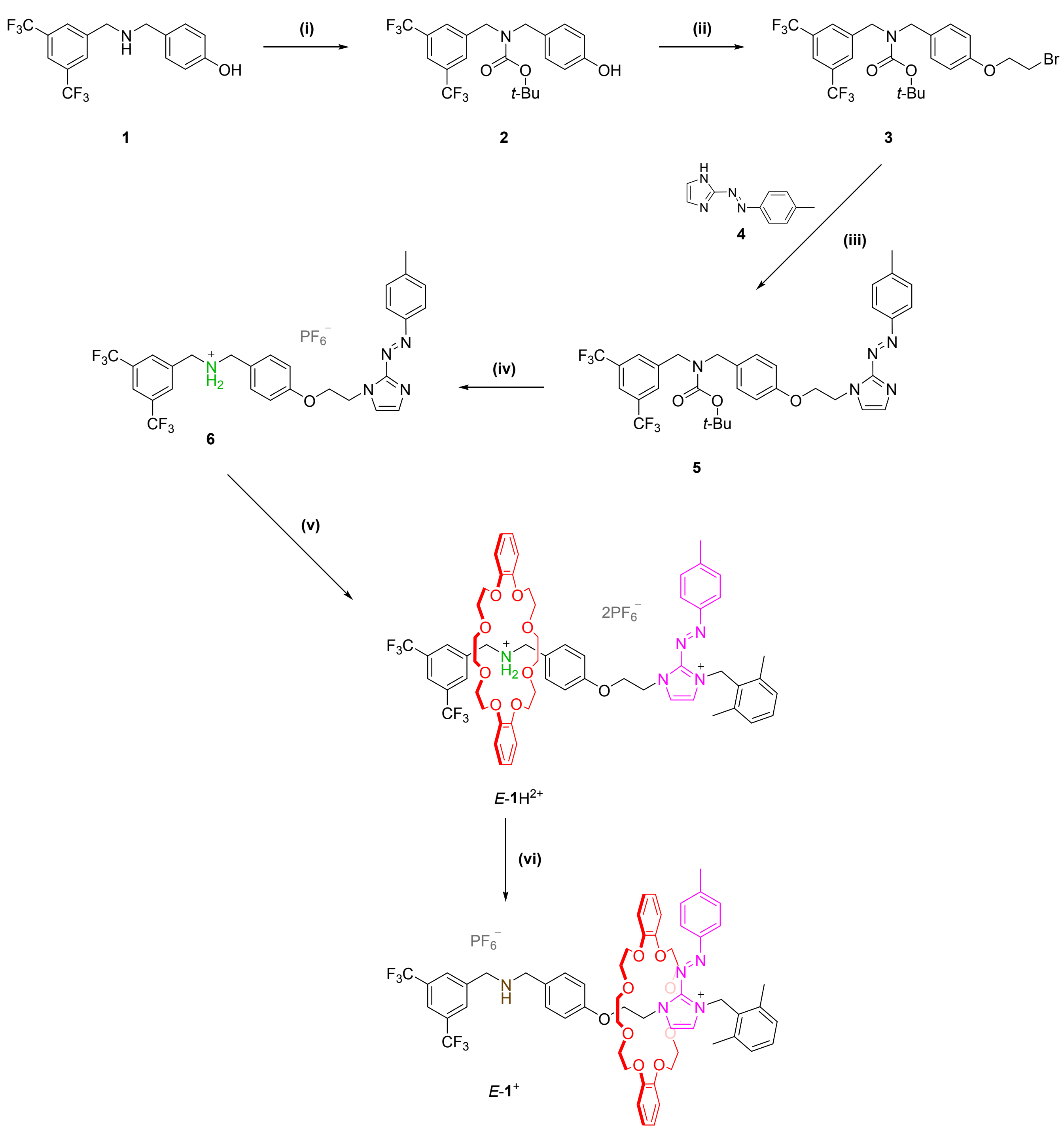


**Figure S1.** Synthesis of *E*-**1**H$^{2+}$ and *E*-**1**$^{+}$. (i) $Boc_2O$, THF; (ii) $BrCH_2CH_2Br$, $K_2CO_3$, MeCN; (iii) **4**, $K_2CO_3$, MeCN; (iv) $HPF_6$, THF; (v) DB24C8, *o*-$Me_2$-BnBr, MeCN; (vi) Base, MeCN.

**4-(((3,5-bis(trifluoromethyl)benzyl)amino)methyl)phenol, 1**

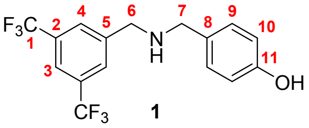

1

An Ethanol solution (200 mL) of 3,5-trifluoromethyl-benzylamine (19.45 g, 80.0 mmol) and 4-hydroxybenzaldehyde (9.77 g, 80.0 mmol) was stirred at 40 °C under reduced pressure to remove the solvent. The procedure was repeated three times providing the imine intermediate product, which was re-dissolved in Ethanol (200 mL). $NaBH_4$ (6.66 g, 176 mmol) was added portionwise and the resulting mixture was stirred at room temperature for 18 h. Removal of the solvent under reduced pressure provided a colourless oil which was dissolved in Ethyl Acetate (100 mL), washed with water (3×100 mL) and dried over $MgSO_4$. Filtration and removal of the solvent under reduced pressure afforded the crude product which, was purified by flash chromatography (Chloroform, $R_f$ = 0.06) to obtain **1** as a pale yellow solid (21.45 g, 77%). $^1$H NMR (500 MHz, 298 K, Chloroform-*d*) δ 7.81 (s, 2H, **4**), 7.77 (s, 1H, **3**), 7.14 (d, *J* = 8.1 Hz, 2H, **9**), 6.70 (d, *J* = 8.1 Hz, 2H, **10**), 3.93 (s, 2H, **6**), 3.76 (s, 2H, **7**). $^{13}$C NMR (126 MHz, 298 K, Chloroform-*d*) δ 155.64, 141.97, 131.85 (q, *J* = 32.8 Hz) 130.36, 129.93, 128.68, 128.65, 128.62, 128.59, 123.44 (q, *J* = 273.4 Hz), 121.38 (p, *J* = 3.8 Hz), 115.90, 52.73, 52.02. $^{19}$F NMR (470 MHz, 298 K, Chloroform-*d*) δ –62.90 (s, 6F, **1**).

***tert*-butyl (3,5-bis(trifluoromethyl)benzyl)(4-hydroxybenzyl)carbamate, 2**

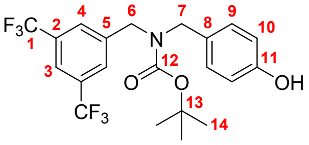

2

A solution of **1** (19.55 g, 55.97 mmol) and di-*tert*-butyl-dicarbonate (12.21 g, 55.97 mmol) in Tetrahydrofuran (600 mL) was stirred at room temperature for 18 h. Removal of the solvent under reduced pressure provided the product as a crude oil which was purified by flash chromatography (Chloroform, $R_f$ = 0.1) to obtain product **2** as an off-white solid (21.96 g, 87%). $^1$H NMR (500 MHz, 298 K, Chloroform-*d*) δ 7.74 (s, 1H, **3**), 7.57 (s, 2H, **4**), 7.04 (m, 2H, **9**), 6.77 (d, *J* = 8.0 Hz, 2H, **10**), 6.42 (s, 1H, OH), 4.42 (m, 4H, **6**+**7**), 1.46 (s, 9H, **14**). $^{13}$C NMR (126 MHz, 298 K, Chloroform-*d*) δ 156.2 (d, *J* = 53.3 Hz), 155.9, 141.0 (d, *J* = 53.3 Hz), 131.9 (q, *J* = 33.3 Hz), 129.5 (d, *J* = 65.4 Hz), 128.75 (d, *J* = 10.5 Hz), 127.7, 123.4 (q, *J* = 273.4 Hz), 121.3, 115.8, 81.5, 50.2, 49.1 (d, *J* = 68.8 Hz), 28.5. $^{19}$F NMR (470 MHz, 298 K, Chloroform-*d*) δ –62.98 (s, 6F).

**tert-butyl (3,5-bis(trifluoromethyl)benzyl)(4-(2-bromoethoxy)benzyl)carbamate, 3**

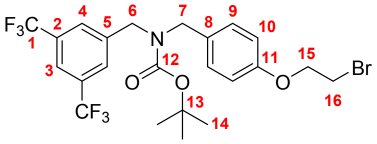


**3**

A solution of **2** (449 Mg, 1.00 mmol), 1,2-dibromoethane (2.15 mL, 25.00 mmol) and caesium carbonate (815 mg, 2.50 mmol) in dry acetonitrile (25 mL) was stirred at 80 °C for 18 h under a nitrogen atmosphere. The solution was cooled down to room temperature and the solvent removed under reduced pressure to provide a crude mixture which was dissolved in Dichloromethane (50 mL), washed with water (3×50 mL) and dried over anhydrous $MgSO_4$. The crude compound was purified by flash chromatography (Chloroform, $R_f = 0.6$) to provide the product **3** as a colorless oil (250 mg, 45%). $^1$H NMR (500 MHz, 298 K, Chloroform-*d*) δ 7.74 (s, 1H, **3**), 7.56 (bs, 2H, **4**), 7.13 (bd, *J* = 25.9 Hz, 2H, **9**), 6.85 (d, *J* = 8.3 Hz, 2H, **10**), 4.60 - 4.30 (m, 4H, **6**+**7**), 4.27 (t, *J* = 6.3 Hz, 2H, **16**), 3.63 (t, *J* = 6.3 Hz, 2H, **15**), 1.49 (m, 9H, **14**). $^{13}$C NMR (126 MHz, 298 K, Chloroform-*d*) δ 157.8, 155.8 (d, *J* = 29.8 Hz), 141.3 (d, *J* = 34.9 Hz), 131.8 (q, *J* = 33.2 Hz), 130.3, 129.5 (d, *J* = 74.8 Hz), 127.7 (d, *J* = 25.2 Hz), 123.4 (q, *J* = 273.4 Hz), 121.2, 115.1, 81.0, 68.1, 50.1, 49.2 (d, *J* = 55.1 Hz), 29.1, 28.4. $^{19}$F NMR (470 MHz, 298 K, Chloroform-*d*) δ –62.94 (s, 6F).

**(*E*)-*p*-methyl-phenylazoimidazole, 4**

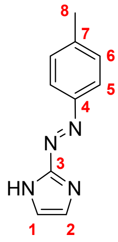


**4**

The product was prepared by modifying a previously reported procedure.[4] Imidazole (2.50 g, 36.70 mmol) was added to a solution of $K_2CO_3$ (5.07 g, 36.70 mmol) in water (80 mL). The solution was cooled down to 0 °C and 4-methylphenyldiazonium hexafluorophosphate (9.69 g, 36.7 mmol) was added protionwise and the resulting solution left to warm up to room temperature and stirred for 18 h. The red-brownish precipitate was collected and dissolved in 1 M HCl and the undissolved dark brown byproduct filtered off. The solution was neutralized with NaOH and the yellow precipitate collected by filtration. Recrystallization from ethanol provided the pure product **4** as dark orange crystals (1.56 g, 23%). $^1$H NMR (500 MHz, 298 K, Dimethyl Sulfoxide-$d_6$) δ 12.91 (s, 1H, NH), 7.76 (d, *J* = 8.1 Hz, 2H, **5**), 7.39 (d, *J* = 8.1 Hz, 2H, **6**), 7.33 (s, 2H, **1**+**2**), 2.39 (s, 3H, **8**). $^{13}$C NMR (126 MHz, 298 K, Dimethyl Sulfoxide-$d_6$) δ 154.60, 150.10, 141.77, 130.07, 122.30, 21.04.

***tert*-butyl-(*E*)-(3,5-bis(trifluoromethyl)benzyl)(4-(2-(2-(p-tolyldiazenyl)-1H-imidazol-1-yl)ethoxy)benzyl)carbamate, 5**

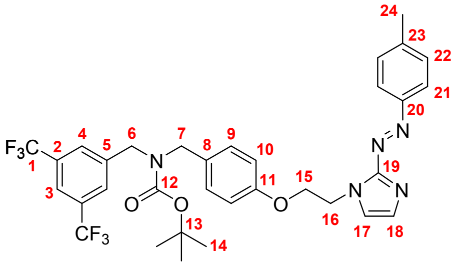

**5**

Intermediate **3** (1.50 g, 2.70 mmol), 1-methyl-2-(phenylazo)imidazole **4** (502 mg, 2.70 mmol), 18-crown-6 (210 mg, 0.81 mmol) and potassium carbonate (750 g, 5.40 mmol) were suspended in dry Acetonitrile (100 mL) and stirred at 80 °C for 24 h under a nitrogen atmosphere. The solvent was removed under reduced pressure and the crude mixture dissolved in Ethyl Acetate (100 mL), washed with water (3×50 mL) and dried over $MgSO_4$. Filtration and removal of the solvent under reduced pressure provided a red-brownish oil which was purified by flash chromatography (Chloroform, $R_f = 0.1$) to obtain the product **5** as a yellow crystalline solid (1.10 g, 63%). $^1$H NMR (500 MHz, 298 K, Chloroform-*d*) δ 7.88 (d, *J* = 8.3 Hz, 2H, **21**), 7.72 (s, 1H, **3**), 7.54 (m, 2H, **4**), 7.35 - 7.27 (m, 4H, **17**+**18**+**22**), 7.11 (m, 2H, **9**), 6.79 (d, *J* = 8.3 Hz, 2H, **10**), 4.81 (t, *J* = 5.2 Hz, 2H, **15**), 4.54 - 4.24 (m, 6H, **16**+**6**+**7**), 2.43 (s, 3H, **24**), 1.60 - 1.37 (m, 9H, **14**). $^{13}$C NMR (126 MHz, 298 K, Chloroform-*d*) δ 157.71, 155.78, 152.23, 151.29, 142.66, 131.70 (q, *J* = 33.2 Hz), 130.37, 130.26, 129.92, 129.75, 129.68, 129.12, 127.61, 123.56, 123.37, 123.33 (q, *J* = 273.4 Hz), 121.16, 114.84, 81.01, 67.41, 50.04, 49.36, 48.97, 45.41, 28.40, 21.69. $^{19}$F NMR (470 MHz, 298 K, Chloroform-*d*) δ –62.92 (s, 6F, **1**).

**(*E*)-N-(3,5-bis(trifluoromethyl)benzyl)-1-(4-(2-(2-(p-tolyldiazenyl)-1H-imidazol-1-yl)ethoxy)phenyl)methanaminium hexafluorophosphate, 6**

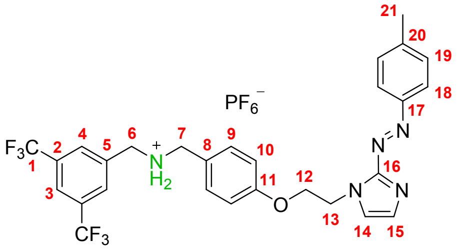

**6**

A solution of 5 (1.00 g, 1.51 mmol) and $HPF_6$ (60%$_{w/w}$ in $H_2O$, 1.33 mL, 15.1 mmol) in Tetrahydrofuran (100 mL) was stirred at room temperature for 3 h. The solvent was removed under reduced pressure and the residue dissolved in Dichloromethane (100 mL) and washed with a saturateed aqueous solution of ammonium hexafluorophosphate (3×100 mL). The organic phases were dried over anhydrous $MgSO_4$, filtration and removal of the solvent under reduced pressure provided the product **6** as an orange solid (555 mg, 52%). $^1$H NMR (500 MHz, 298 K, Dimethyl Sulfoxide-*d*$_6$) δ 8.16 (s, 2H, **4**), 8.11 (s, 1H, **3**), 7.75 (d, *J* = 8.0 Hz, 2H, **18**), 7.68 (s, 1H, **14**), 7.36 (d, *J* = 8.1 Hz, 2H, **19**), 7.32 (d, *J* = 8.3 Hz, 2H, **9**), 7.25 (s, 1H, **15**), 6.92 (d, *J* = 8.3 Hz, 2H, **10**), 4.82 (t, *J* = 5.2 Hz, 2H, **12**), 4.40 (t, *J* = 5.2 Hz, 2H, **13**), 4.25 (s, 2H, **6**), 4.03 (s, 2H, **7**), 2.39 (s, 3H, **21**). $^{13}$C NMR (126 MHz, 298 K, Dimethyl Sulfoxide-*d*$_6$) δ 158.14, 151.89, 150.81, 141.88, 141.79, 131.09, 130.63, 130.13 (q, *J* = 33.2 Hz), 130.07, 129.95, 129.90, 129.86, 124.62, 123.26 (q, *J* = 273.4 Hz), 122.57, 122.31, 114.55, 67.17, 50.46, 49.22, 44.85, 21.03. $^{19}$F NMR (470 MHz, 298 K, Acetonitrile-*d*$_3$) δ –63.63 (s, 6F, **1**), –72.54 (d, *J* = 707.4 Hz, $PF_6$).

***E*-1H$^{2+}$**

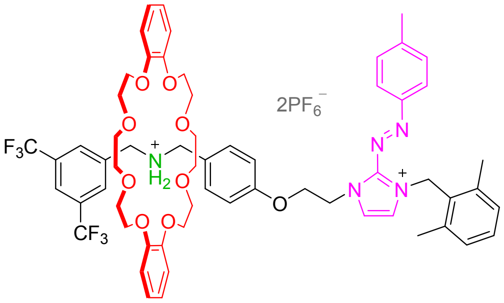


*E*-**1**H$^{2+}$

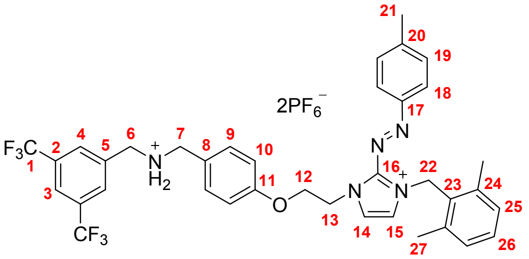


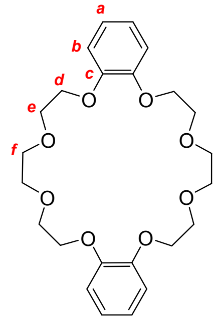

A solution of **6** (230 mg, 0.326 mmol) and dibenzo-24-crown-8 (292 mg, 0.652 mmol) in Acetonitrile (15 mL) was stirred at room temperature for 30 min. Neat 2-(Bromomethyl)-1,3-dimethylbenzene (325 mg, 1.63 mmol) was then added and the resulting mixture stirred at 80 °C in a microwave reactor for 2 h. The solution was cooled down to room temperature and the solvent removed under reduced pressure to provide a crude mixture which was dissolved in Dichloromethane (50 mL), washed with water (3×50 mL) and a saturated aqueous solution of potassium hexafluorophosphate (3×20 mL) and dried over anhydrous $MgSO_4$. The crude compound was purified by flash chromatography (Dichloromethane, then 90:10 Dichloromethane:Methanol, then 0.1 M $H_4NPF_6$ in Methanol) and repeated Gel Permeation Chromatography (Dichloromethane) to provide the product *E*-**1**H$^{2+}$ as a bright orange solid (274 mg, 65%). $^1$H NMR (500 MHz, 298 K, Acetonitrile-$d_3$) δ 8.04 (d, *J* = 8.3 Hz, 2H, **18**), 7.85 (d, *J* = 1.7 Hz, 2H, **4**), 7.74 (s, 2H, $NH_2$), 7.65 (d, *J* = 2.2 Hz, 1H, **14**), 7.51 (d, *J* = 8.2 Hz, 2H, **19**), 7.39 (d, *J* = 8.3 Hz, 2H, **9**), 7.34 - 7.27 (m, 2H, **3**+**26**), 7.20 (d, *J* = 7.6 Hz, 2H, **25**), 6.93 (d, *J* = 2.2 Hz, 1H, **15**), 6.87 (d, *J* = 8.3 Hz, 2H, **10**), 6.68 (dd, *J* = 6.1, 3.5 Hz, 4H, ***b***), 6.59 (dd, J = 6.0, 3.6 Hz, 4H, ***a***), 5.70 (s, 2H, **22**), 5.03 - 4.97 (m, 2H, **6**), 4.90 (t, *J* = 4.8 Hz, 2H, **13**), 4.50 - 4.46 (m, 2H, **7**), 4.40 (t, *J* = 4.8 Hz, 2H, **12**), 4.03 (ddd, *J* = 11.3, 7.9, 1.5 Hz, 4H, ***d***), 3.87 - 3.79 (m, 8H, ***d***+***e***), 3.74 - 3.65 (m, 12H, ***e***+***f***), 2.50 (s, 3H, **21**), 2.28 (s, 6H, **27**). $^{13}$C NMR (126 MHz, 298 K, Acetonitrile-$d_3$) δ 159.85 (**11**), 151.97 (**17**), 149.28 (**20**), 147.55 (***c***), 144.86 (**16**), 139.84 (**24**), 136.14 (**5**), 132.34 (**9**), 131.70 (**19**), 131.21 (q, *J* = 32.8 Hz, **2**), 131.04 (**4**), 130.99 (**26**), 130.01 (**25**), 129.67 (**23**), 125.60 (**14**), 125.54 (**18**), 125.04 (**8**), 124.09 (q, *J* = 273.4 Hz, **1**), 122.54 (m, **3**), 122.06 (**15**), 122.01 (***b***), 115.97 (**10**), 112.76 (***a***), 71.62 (***f***), 71.03 (***e***), 68.48 (***d***), 67.01 (**12**), 53.30 (**7**), 51.68 (**6**), 49.93 (**13**), 49.26 (**22**), 22.12 (**21**), 19.85 (**27**). $^{19}$F NMR (470 MHz, 298 K, Acetonitrile-$d_3$) δ –63.00 (s, 6F, **1**), –72.89 (d, *J* = 706.5 Hz, 12F, $PF_6$). HRMS-ESI (m/z): calcd for [$C_{61}H_{69}N_5O_9PF_{12}$], 1274.4641; found 1274.4641 [(**1**H)($PF_6$)]$^+$.

**Procedure for the deprotonation of compound** *E*-1H$^{2+}$

A solution of *E*-**1**H$^{2+}$ (15 mg, 0.013 mmol) in Acetonitrile-$d_3$ (500 µL) was reacted with **B1** (20 mg) inside an NMR tube. The suspension was agitated for fixed time intervals and the reaction progress monitored by $^1$H NMR spectroscopy. Upon complete disappearance of the peaks of *E*-**1**H$^{2+}$, the mixture was filtered through a pad of Celite to isolate the solution containing *E*-**1**$^+$.

***E*-1**+

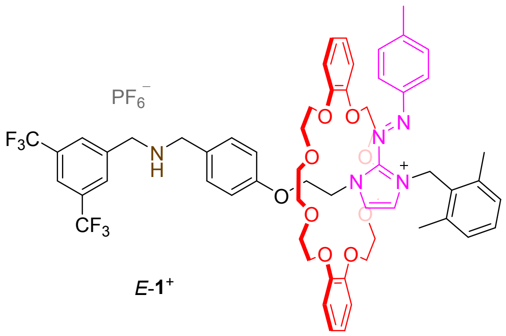


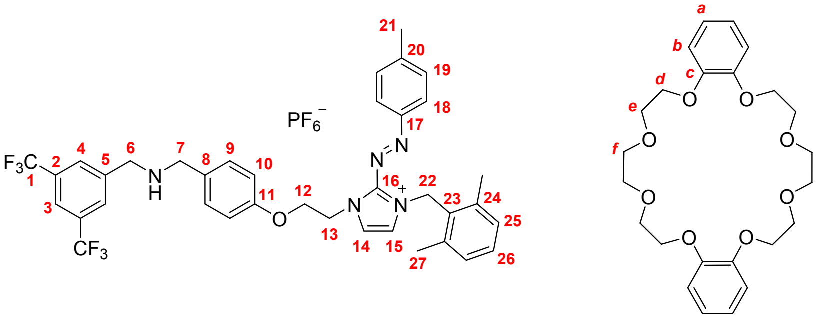

Synthesised according to the general deprotonation procedure. $^{1}$H NMR (500 MHz, 298 K, Acetonitrile-*d*$_3$) δ 8.57 (d, *J* = 2.2 Hz, 1H, **14**), 8.25 (d, *J* = 8.1 Hz, 2H, **18**), 7.94 (s, 2H, **4**), 7.87 (s, 1H, **3**), 7.43 (d, *J* = 8.0 Hz, 2H, **19**), 7.26 (t, *J* = 7.6 Hz, 1H, **26**), 7.13 (d, *J* = 7.6 Hz, 2H, **25**), 7.03 (d, *J* = 8.1 Hz, 2H, **9**), 6.80 (ddt, *J* = 22.3, 6.3, 3.7 Hz, 8H, ***b***+***a***), 6.71 (d, *J* = 2.2 Hz, 1H, **15**), 6.61 (d, *J* = 8.1 Hz, 2H, **10**), 5.60 (t, *J* = 7.9 Hz, 2H, **13**), 5.36 (s, 2H, **22**), 4.84 (t, *J* = 7.9 Hz, 2H, **12**), 4.05 - 3.92 (m, 8H, ***d***), 3.86 (s, 2H, **6**), 3.66 (m, 10H, **7**+***e***), 3.43 (ddd, J = 87.5, 10.9, 6.5 Hz, 8H, ***f***), 2.47 (s, 3H, **21**), 2.08 (s, 6H, **27**). $^{13}$C NMR (126 MHz, 298 K, Acetonitrile-*d*$_3$) δ 158.85 (**11**), 152.02 (**17**), 148.86 (***c***), 148.34 (**20**), 145.70 (**5**), 144.36 (**16**), 139.49 (**24**), 133.03 (**8**), 131.71 (q, *J* = 32.8 Hz, **2**), 131.37 (**19**), 130.71 (**26**), 130.33 (**23**), 129.93 (**9**), 129.85 (**25**), 129.52 (**4**), 126.99 (**14**), 126.06 (**18**), 124.84 (q, *J* = 273.4 Hz, **1**), 121.83 (***b***), 121.47 (**3**), 120.93 (**15**), 115.20 (**10**), 112.91 (***a***), 71.59 (***f***), 70.92 (***e***), 69.09 (***d***), 65.57 (**12**), 53.00 (**7**), 52.29 (**6**), 49.44 (**22**), 47.77 (**13**), 22.03 (**21**), 19.92 (**27**). $^{19}$F NMR (470 MHz, 298 K, Acetonitrile-*d*$_3$) δ –63.25 (s, 6F, **1**), –72.97 (d, *J* = 706.3 Hz, 6F, $PF_6$). HRMS-ESI (m/z): calcd for [$C_{61}H_{68}N_5O_9$], 1128.4921; found 1128.4921 [**1**]$^{+}$.

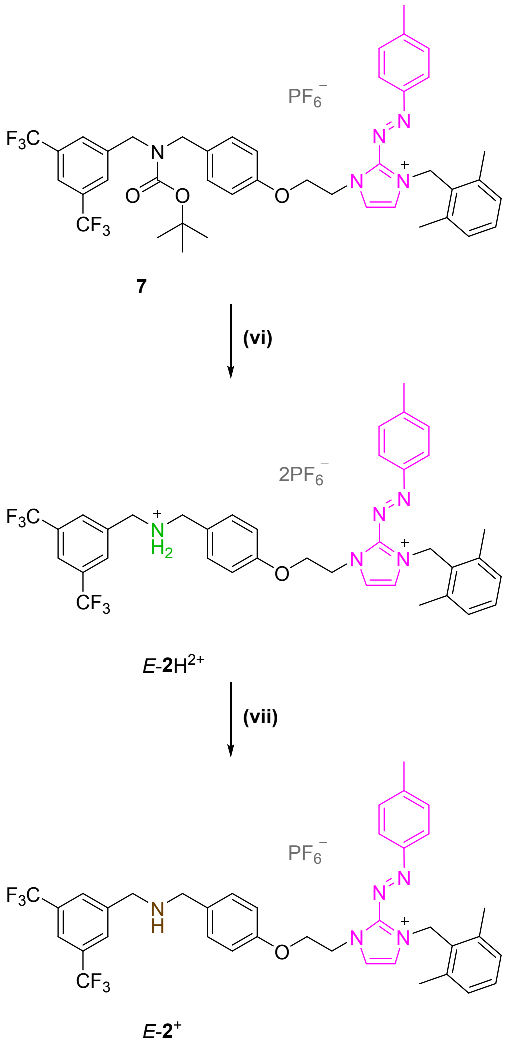


**Figure S2.** Synthesis of *E*-**2**$H^{2+}$ and *E*-**2**$^{+}$. (vi) $HPF_6$, THF; (vii) Base, MeCN.

**(*E*)-1-(2-(4-(((3,5-bis(trifluoromethyl)benzyl)(tert-butoxycarbonyl)amino)methyl)phenoxy)ethyl)-3-(2,6-dimethylbenzyl)-2-(p-tolyldiazenyl)-1H-imidazol-3-ium hexafluorophosphate, 7**

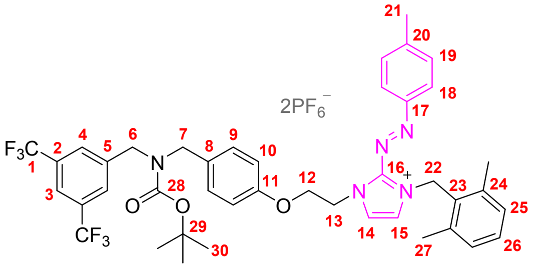


**7**

A solution of intermediate **5** (500 mg, 0.76 mmol) and 2-(Bromomethyl)-1,3-dimethylbenzene (756 mg, 3.8 mmol) in Acetonitrile (20 mL) was stirred at 80 °C for 30 min. The solvent was removed under reduced pressure and the solid residue dissolved in Chloroform (50 mL) and washed with a saturated aqueous solution of ammonium hexafluorophosphate (3×50 mL) and water (3×50 mL). The organic phases were dried over anhydrous MgSO4, filtered and the solvent removed under reduced pressure to give a crude product that was purified by flash chromatography (Chloroform, then Chloroform:Methanol gradient) to provide product **7** as an orange solid (660 mg, 93%). $^1$H NMR (500 MHz, 298 K, Chloroform-*d*) δ 7.96 (d, *J* = 8.0 Hz, 2H, **18**), 7.69 (s, 1H, **3**), 7.67 (d, *J* = 2.2 Hz, 1H, **14**), 7.50 (s, 2H, **4**), 7.41 (d, *J* = 8.2 Hz, 2H, **19**), 7.27 (t, *J* = 7.6 Hz, 1H, **26**), 7.14 (d, *J* = 7.6 Hz, 2H, **25**), 7.06 (s, 2H, **9**), 6.77 (d, *J* = 2.2 Hz, 1H, **15**), 6.72 (d, *J* = 8.1 Hz, 2H, **10**), 5.70 (s, 2H, **22**), 4.99 (t, *J* = 4.8 Hz, 2H, **13**), 4.47 - 4.25 (m, 6H, **6**+**7**+**12**), 2.49 (s, 3H, **21**), 2.28 (s, 6H, **27**), 1.58 - 1.38 (m, 9H, **30**). $^{13}$C NMR (126 MHz, 298 K, Chloroform-*d*) δ 157.28, 155.68, 151.20, 148.44, 143.92, 141.47, 138.65, 131.68 (q, *J* = 34.0 Hz), 130.87, 130.56, 130.44, 129.72, 129.45, 127.92, 127.66, 124.95, 124.53, 123.36 (q, *J* = 273.4 Hz), 121.09, 120.85, 114.74, 81.03, 65.96, 50.12, 49.48, 49.20, 48.54, 28.42, 22.23. $^{19}$F NMR (470 MHz, 298 K, Chloroform-*d*) δ –62.91 (s, 6f, **1**), –73.57 (d, *J* = 712.4 Hz, 6F, $PF_6$).

***E*-2H$^{2+}$**

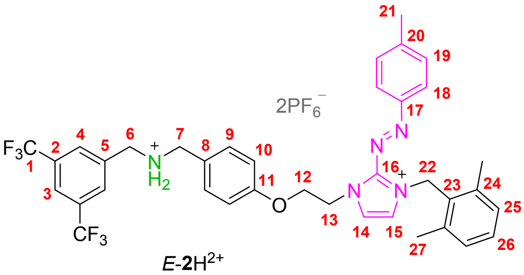


*E*-**2**H$^{2+}$

A solution of **7** (550 mg, 0.59 mmol) and $HPF_6$ (60%$_{w/w}$ in $H_2O$, 522 µL, 5.9 mmol) in Tetrahydrofuran (50 mL) was stirred at room temperature for 3 h. The solvent was removed under reduced pressure and the residue dissolved in Dichloromethane (50 mL) and washed with a saturated aqueous solution of ammonium hexafluorophosphate (3×50 mL). The organic phases were dried over anhydrous $MgSO_4$, filtration and removal of the solvent under reduced pressure provided product *E*-**2**H$^{2+}$ as an orange solid (235 mg, 41%). $^1$H NMR (500 MHz, 298 K, Acetonitrile-*d$_3$*) δ 8.07 - 7.98 (m, 5H, **18**+**3**+**4**), 7.65 (d, *J* = 2.2 Hz, 1H, **14**), 7.53 - 7.49 (m, 2H, **19**), 7.31 (dt, *J* = 8.0, 3.2 Hz, 3H, **26**+**9**), 7.20 (d, *J* = 7.6 Hz, 2H, **25**), 6.92 - 6.87 (m, 3H, **10**+**15**), 5.69 (s, 2H, **22**), 4.90 (t, *J* = 4.7 Hz, 2H, **13**), 4.43 (t, *J* = 4.7 Hz, 2H, **12**), 4.22 (s, 2H, **6**), 4.03 (s, 2H, **7**), 2.50 (s, 3H, **21**), 2.28 (s, 6H, **27**). $^{13}$C NMR (126 MHz, 298 K, Acetonitrile-*d$_3$*) δ 159.44 (**11**), 151.97 (**17**), 149.24 (**20**), 144.82 (**16**), 139.86 (**24**), 137.27 (**5**), 132.32 (q, *J* = 32.8 Hz, **2**), 132.27 (**9**), 131.69 (**19**), 131.46 (**4**), 131.00 (**26**), 130.00 (**25**), 129.64 (**23**), 126.79 (**8**), 125.62 (**14**), 125.51 (**18**), 124.38 (q, *J* = 273.42 Hz, **1**), 123.82 (**3**), 121.97 (**15**), 115.73 (**10**), 66.98 (**12**), 52.47 (**7**), 51.35 (**6**), 49.98 (**13**), 49.26 (**22**), 22.10 (**21**), 19.83 (**27**). $^{19}$F NMR (470 MHz, 298 K, Acetonitrile-*d$_3$*) δ –63.51 (s, 6F, **1**), –72.76 (d, *J* = 707.1 Hz, 12F, $PF_6$). HRMS-ESI (m/z): calcd for [$C_{37}H_{37}N_5OPF_{12}$], 826.2544; found 826.2544 [(**2**H)($PF_6$)]$^+$.

***E*-2⁺**

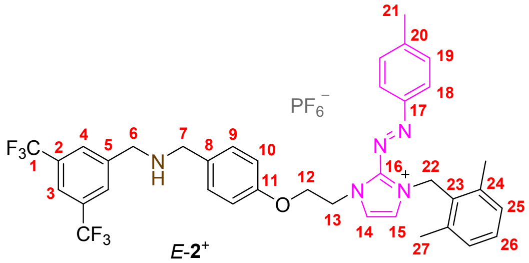


A solution of *E*-**2**H$^{2+}$ (25 mg, 0.026 mmol) in Acetonitrile (5 mL) was loaded on a silica pad and eluted with 20 mL of solvent. Removal of the solvent under reduced pressure provided the product as an orange solid (21 mg, quantitative). $^{1}$H NMR (500 MHz, 298 K, Acetonitrile-$d_3$) δ 8.01 (d, $J$ = 8.3 Hz, 2H, **18**), 7.91 (d, $J$ = 1.6 Hz, 2H, **4**), 7.85 (s, 1H, **3**), 7.64 (d, $J$ = 2.2 Hz, 1H, **14**), 7.49 (d, $J$ = 8.3 Hz, 2H, **19**), 7.31 (dd, $J$ = 8.1, 7.1 Hz, 1H, **26**), 7.22 - 7.17 (m, 4H, **25**+**9**), 6.90 (d, $J$ = 2.2 Hz, 1H, **15**), 6.80 (d, $J$ = 8.3 Hz, 2H, **10**), 5.68 (s, 2H, **22**), 4.88 (t, $J$ = 6.3 Hz, 2H, **13**), 4.41 (t, $J$ = 6.3 Hz, 2H, **12**), 3.85 (s, 2H, **6**), 3.64 (s, 2H, **7**), 2.49 (s, 3H, **21**), 2.28 (s, 6H, **27**). $^{13}$C NMR (126 MHz, 298 K, Acetonitrile-$d_3$) δ 157.85, 151.96, 149.19, 145.62, 144.83, 139.85, 134.64, 131.86, 131.66, 131.60, 130.98, 130.44, 129.99, 129.65, 129.46, 125.57, 125.49, 121.96, 121.49, 115.25, 68.30, 66.87, 52.95, 52.40, 50.05, 49.25, 26.26, 22.08, 19.82. $^{19}$F NMR (470 MHz, 298 K, Acetonitrile-$d_3$) δ –63.31 (s, 6F, **1**), –72.96 (d, $J$ = 706.3 Hz, 6F, $PF_6$). HRMS-ESI (m/z): calcd for [$C_{37}H_{36}N_5OF_6$], 680.2824; found 680.2824 [**2**]$^+$.

## 3. NMR data

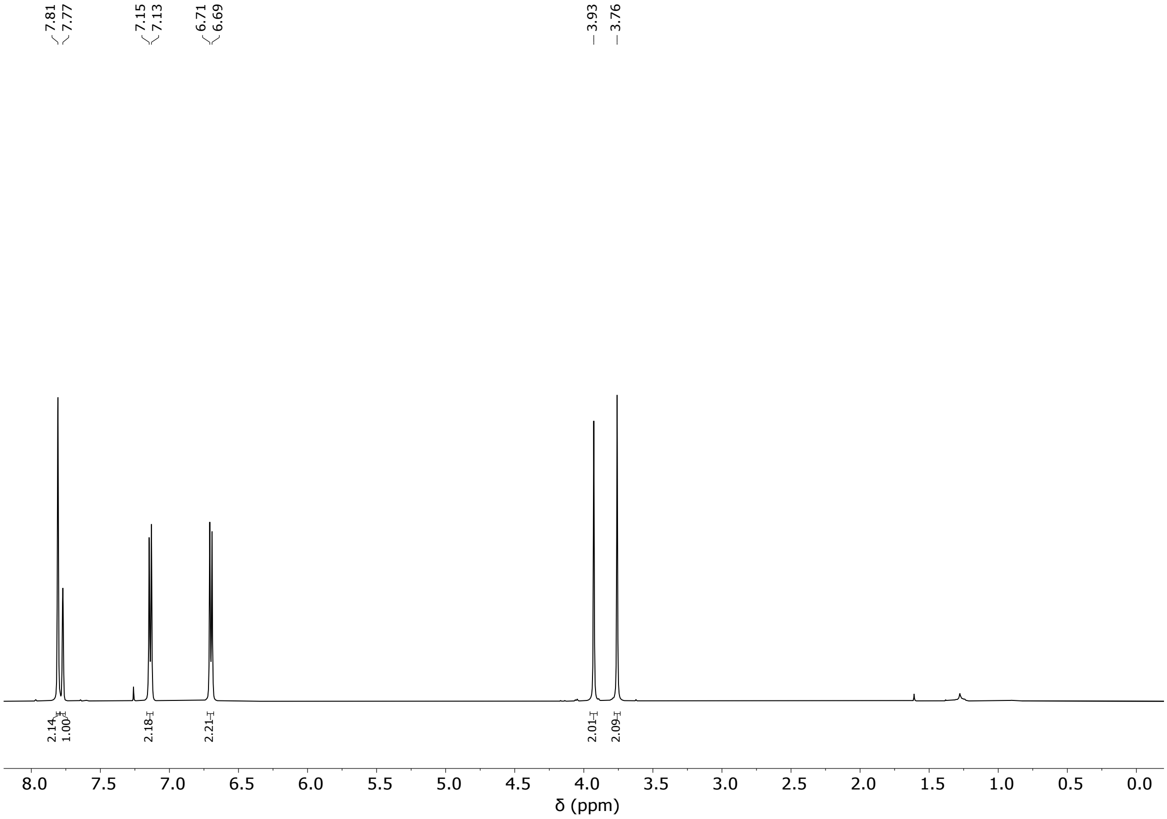


**Figure S3.** $^{1}H$ NMR spectrum of **1** (Chloroform-*d*, 298 K, 400 MHz).

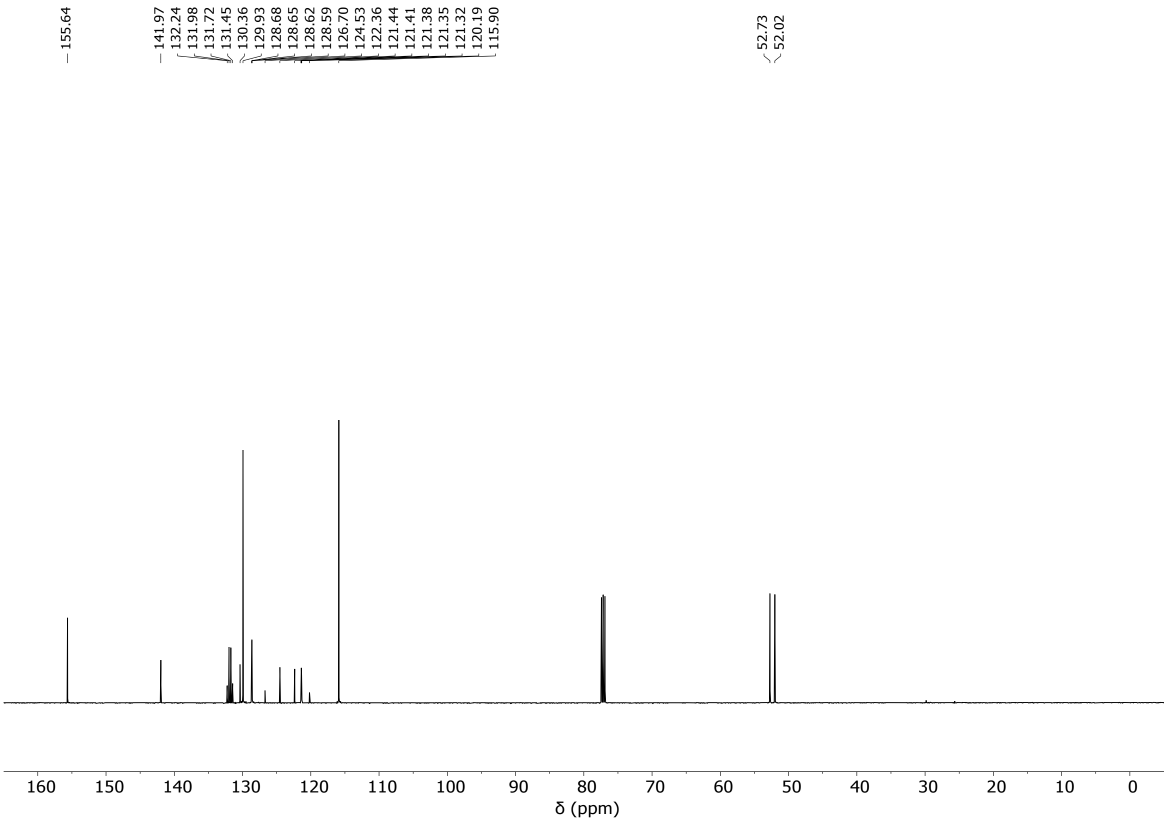


**Figure S4.** $^{13}C$ NMR spectrum of **1** (Chloroform-*d*, 298 K, 101 MHz).

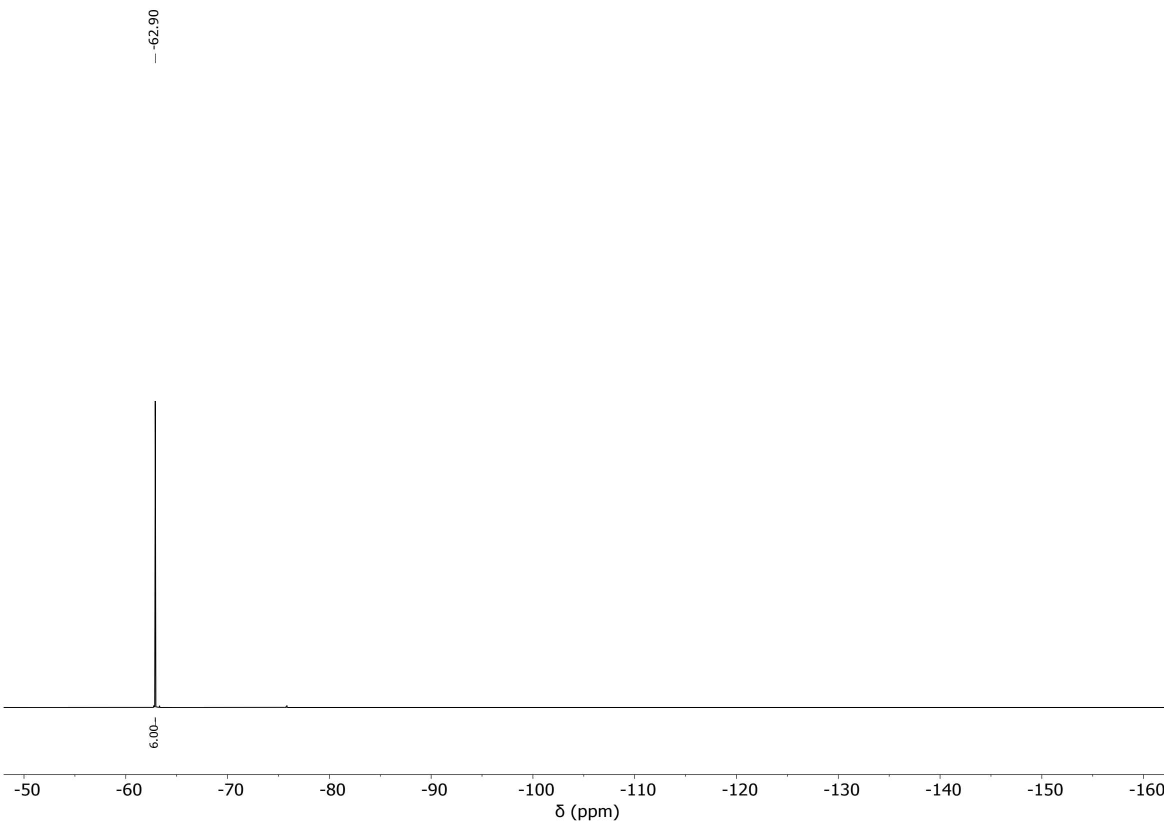


**Figure S5.** $^{19}$F NMR spectrum of **1** (Chloroform-*d*, 298 K, 470 MHz).

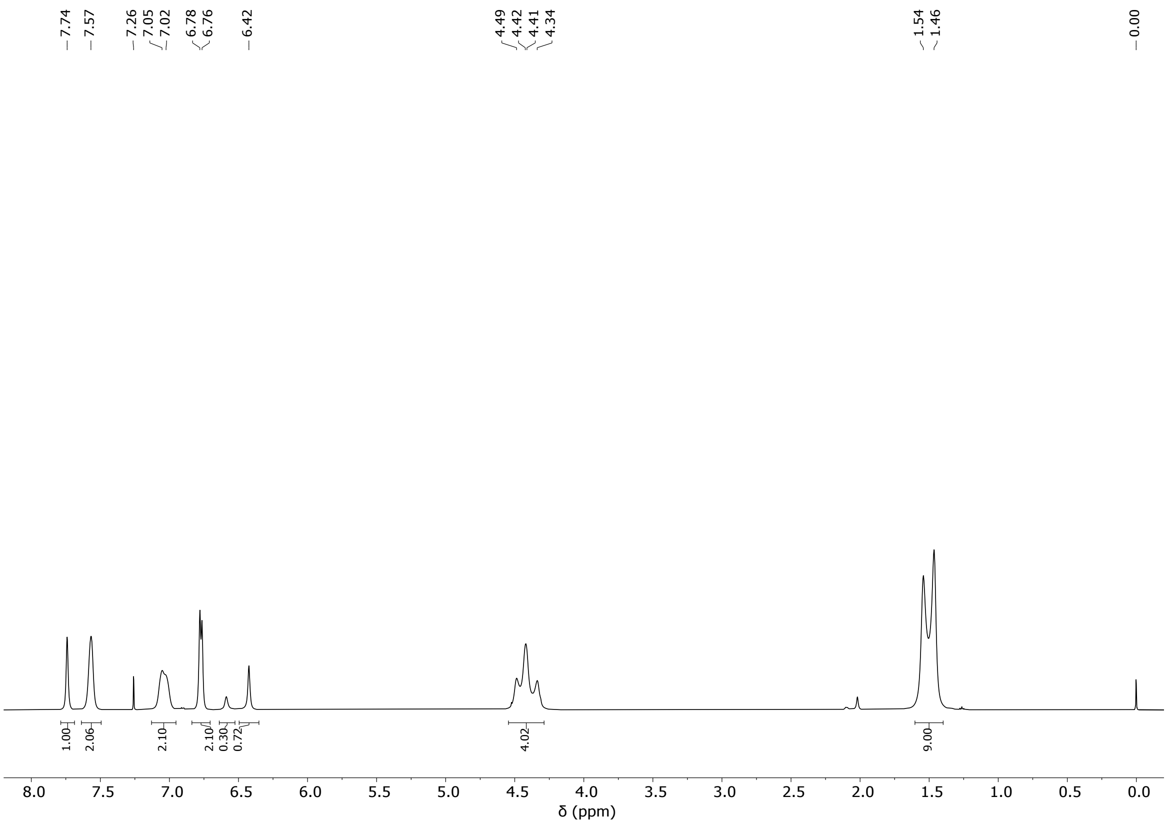


**Figure S6.** $^{1}$H NMR spectrum of **2** (Chloroform-*d*, 298 K, 400 MHz).

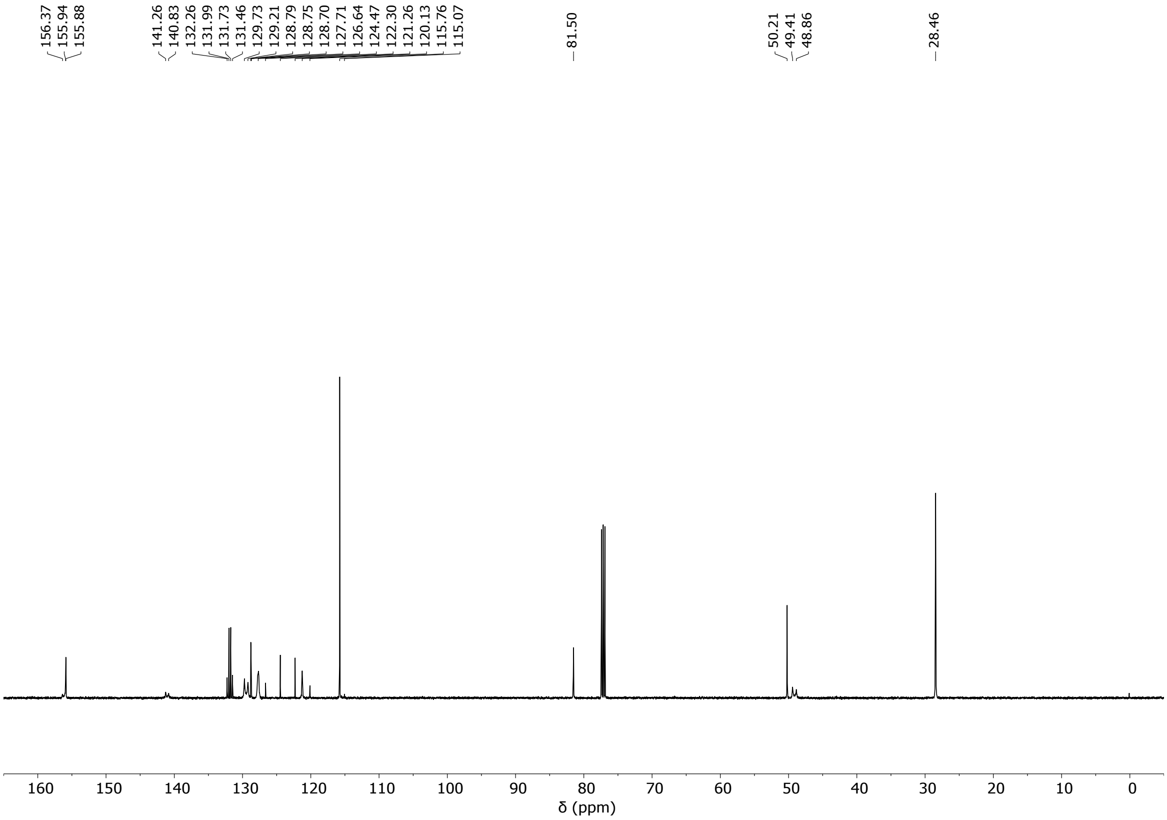


**Figure S7.** $^{13}$C NMR spectrum of **2** (Chloroform-*d*, 298 K, 101 MHz).

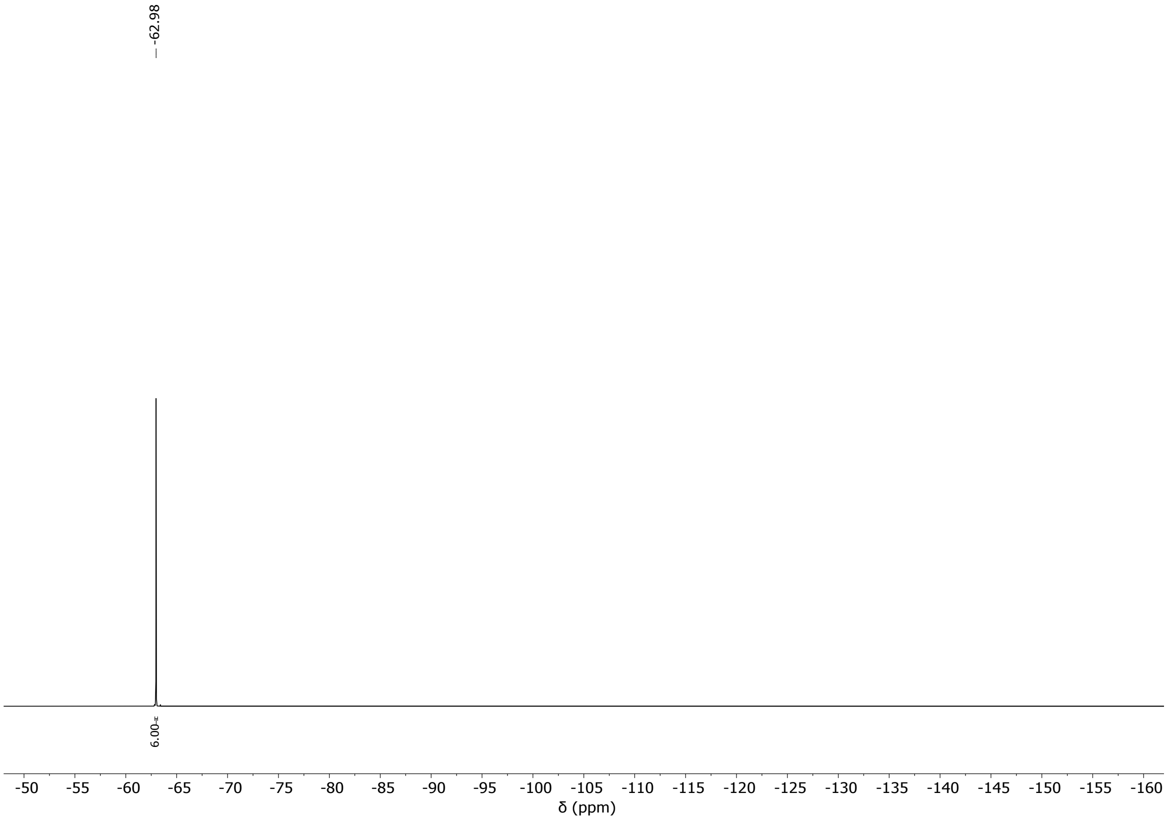


**Figure S8.** $^{19}$F NMR spectrum of **2** (Chloroform-*d*, 298 K, 470 MHz).

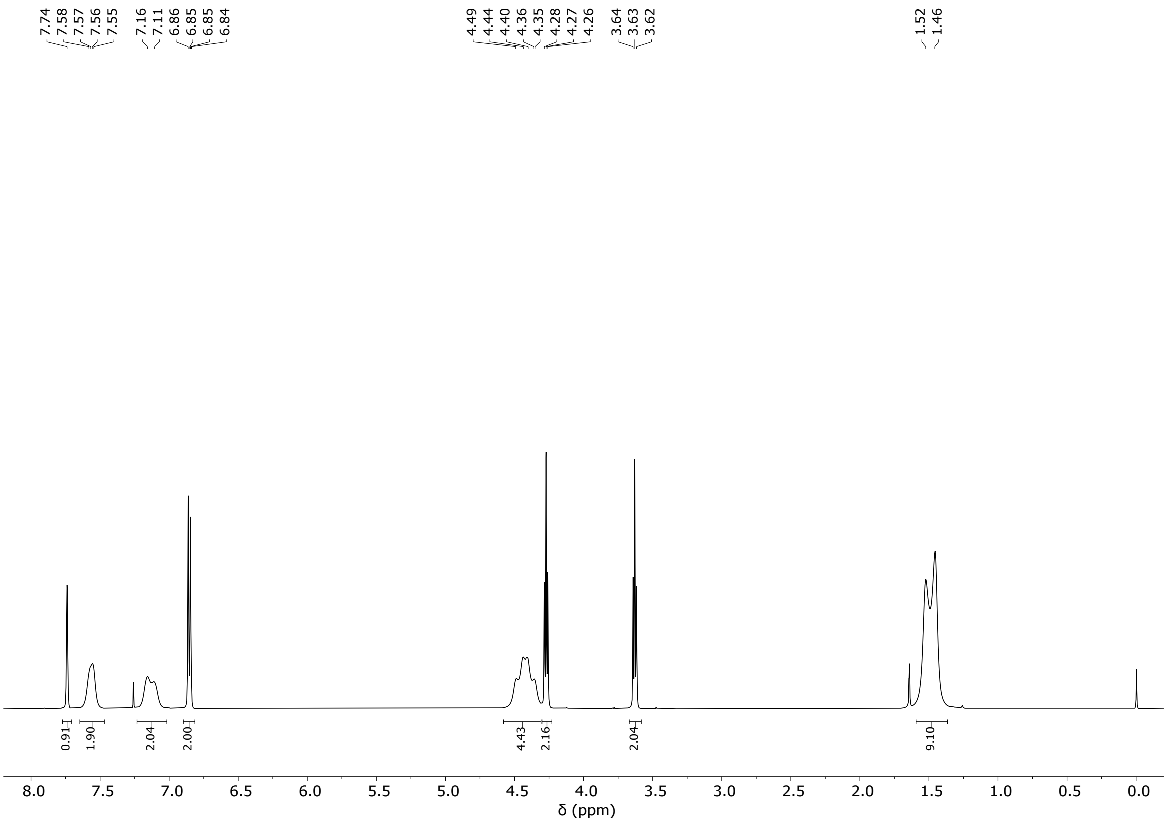


**Figure S9.** $^{1}H$ NMR spectrum of **3** (Chloroform-*d*, 298 K, 400 MHz).

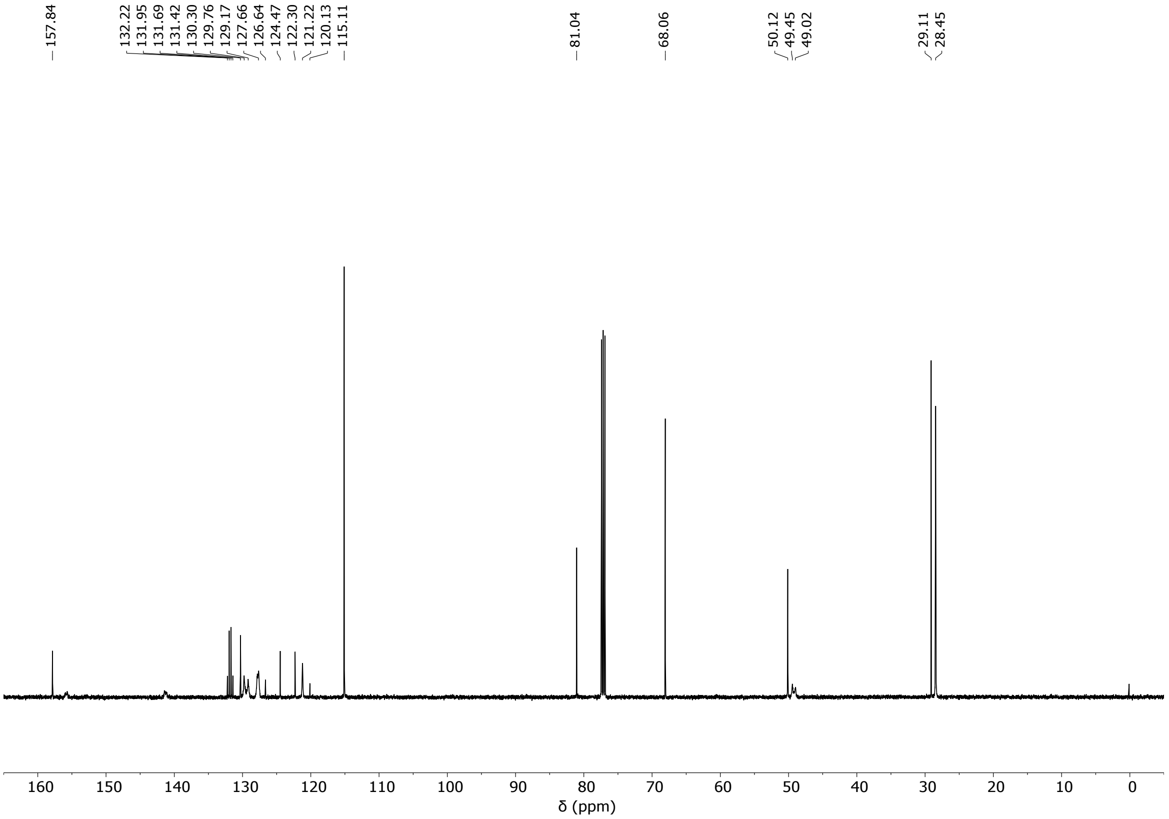


**Figure S10.** $^{13}C$ NMR spectrum of **3** (Chloroform-*d*, 298 K, 126 MHz).

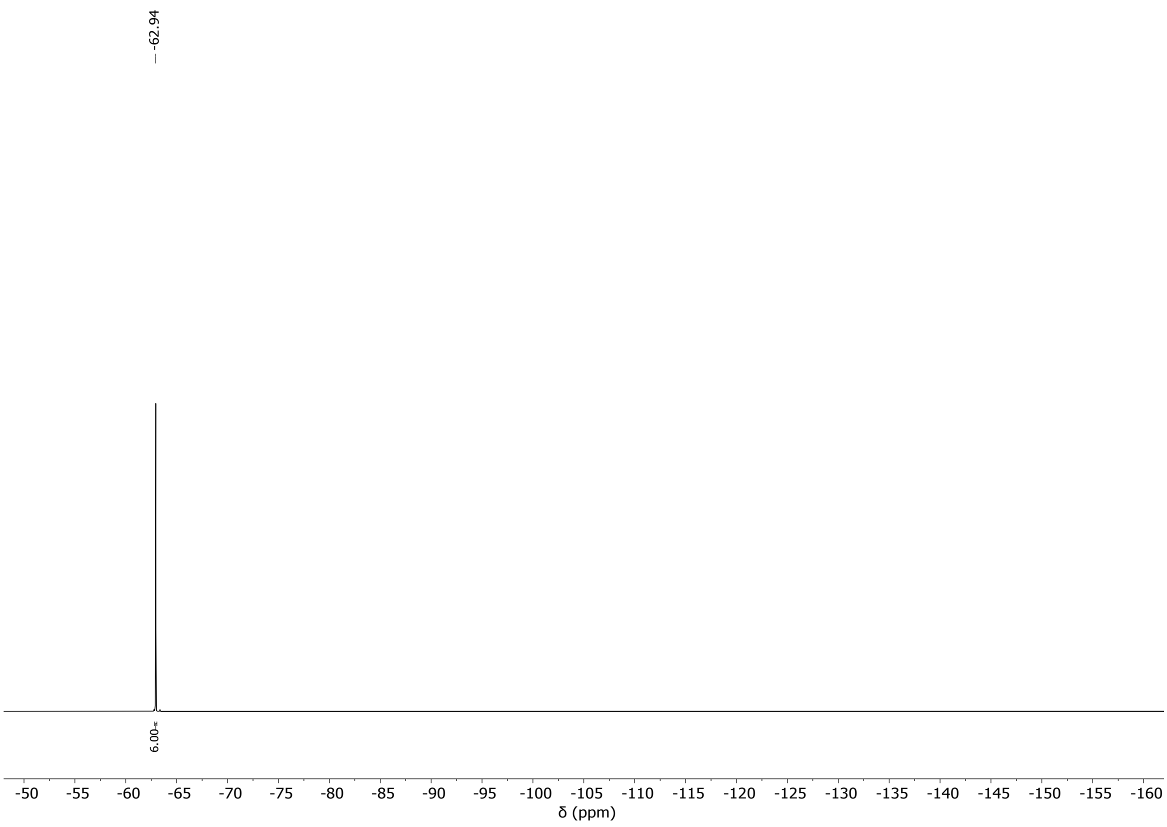


**Figure S11.** $^{19}$F NMR spectrum of **3** (Chloroform-*d*, 298 K, 470 MHz).

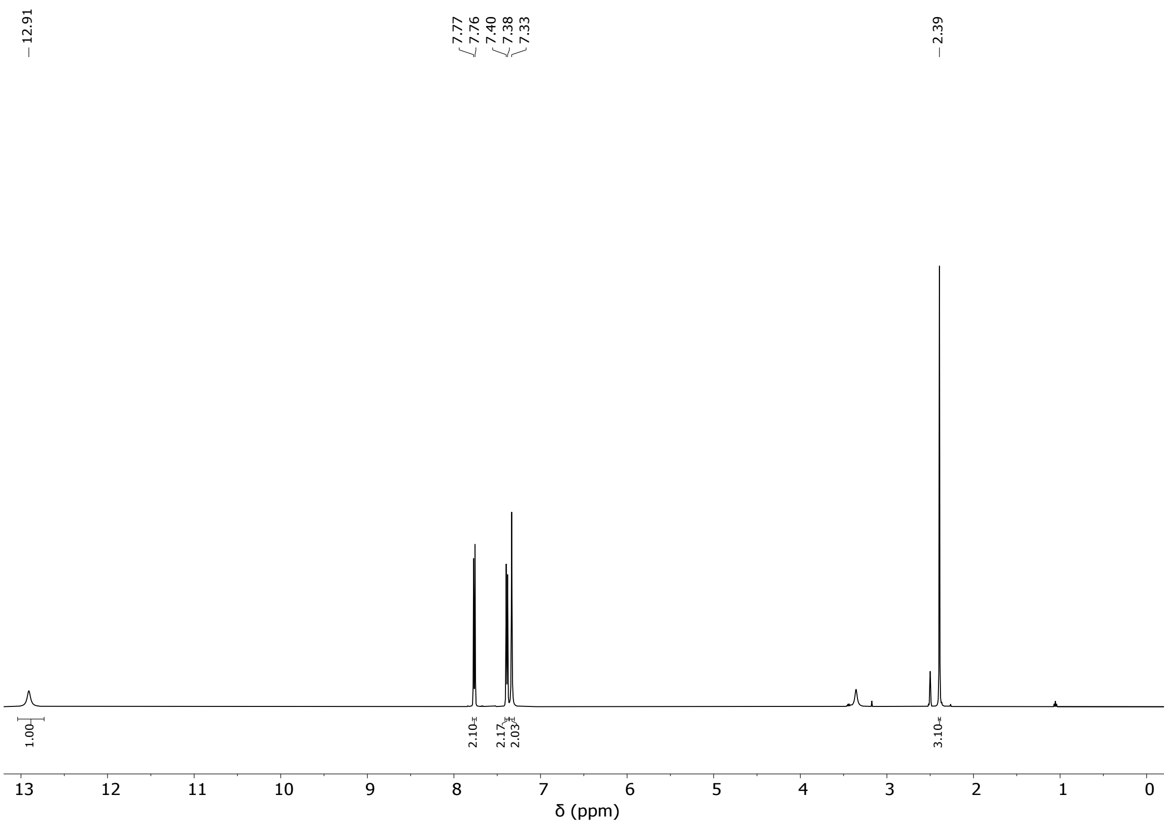


**Figure S12.** $^{1}$H NMR spectrum of **4** (Chloroform-*d*, 298 K, 400 MHz).

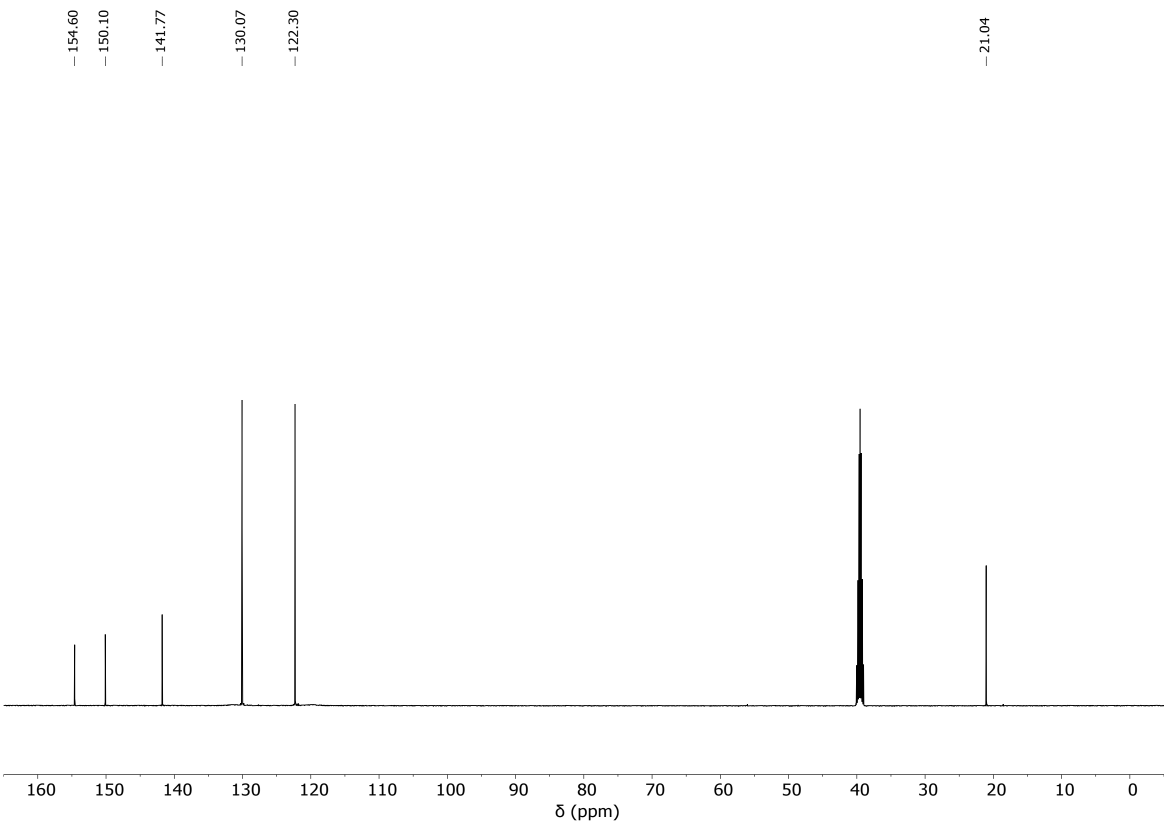


**Figure S13.** $^{13}$C NMR spectrum of **4** (Chloroform-*d*, 298 K, 126 MHz).

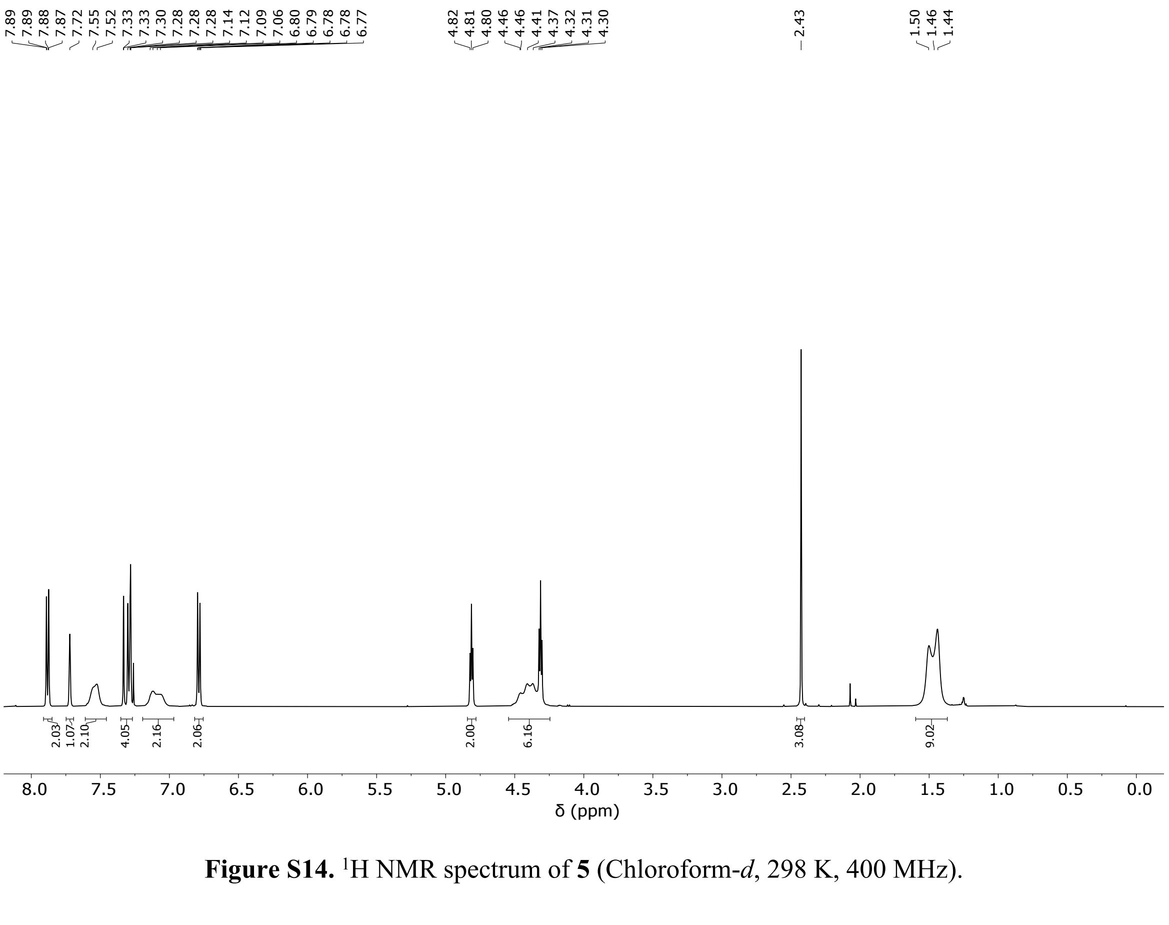


**Figure S14.** $^{1}$H NMR spectrum of **5** (Chloroform-*d*, 298 K, 400 MHz).

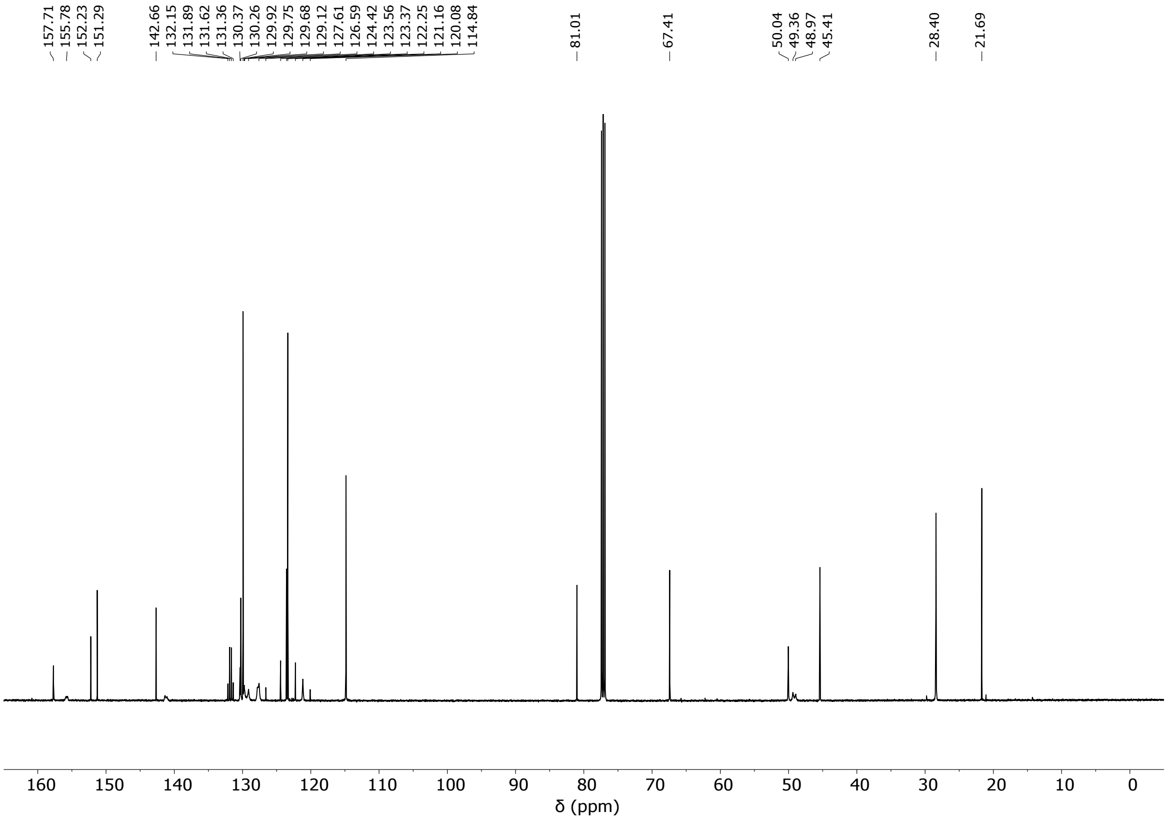


**Figure S15.** $^{13}C$ NMR spectrum of **5** (Chloroform-*d*, 298 K, 126 MHz).

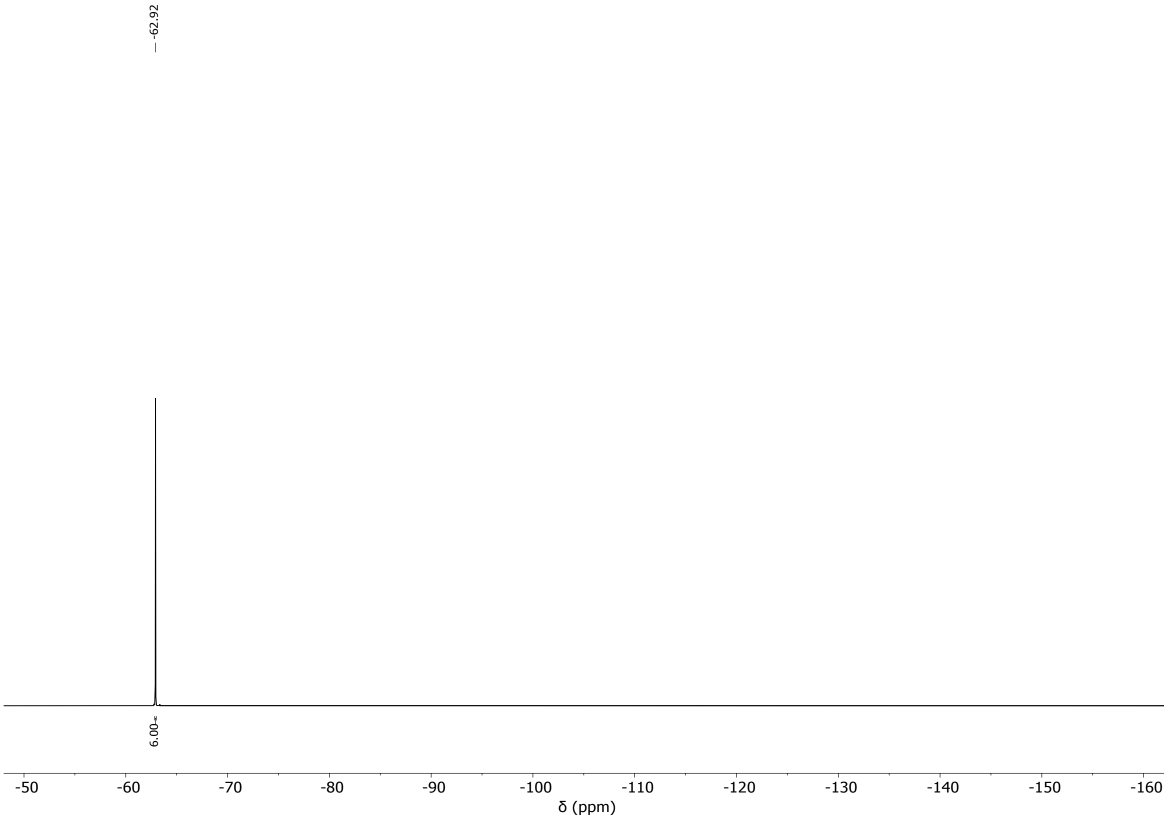


**Figure S16.** $^{19}F$ NMR spectrum of **5** (Chloroform-*d*, 298 K, 470 MHz).

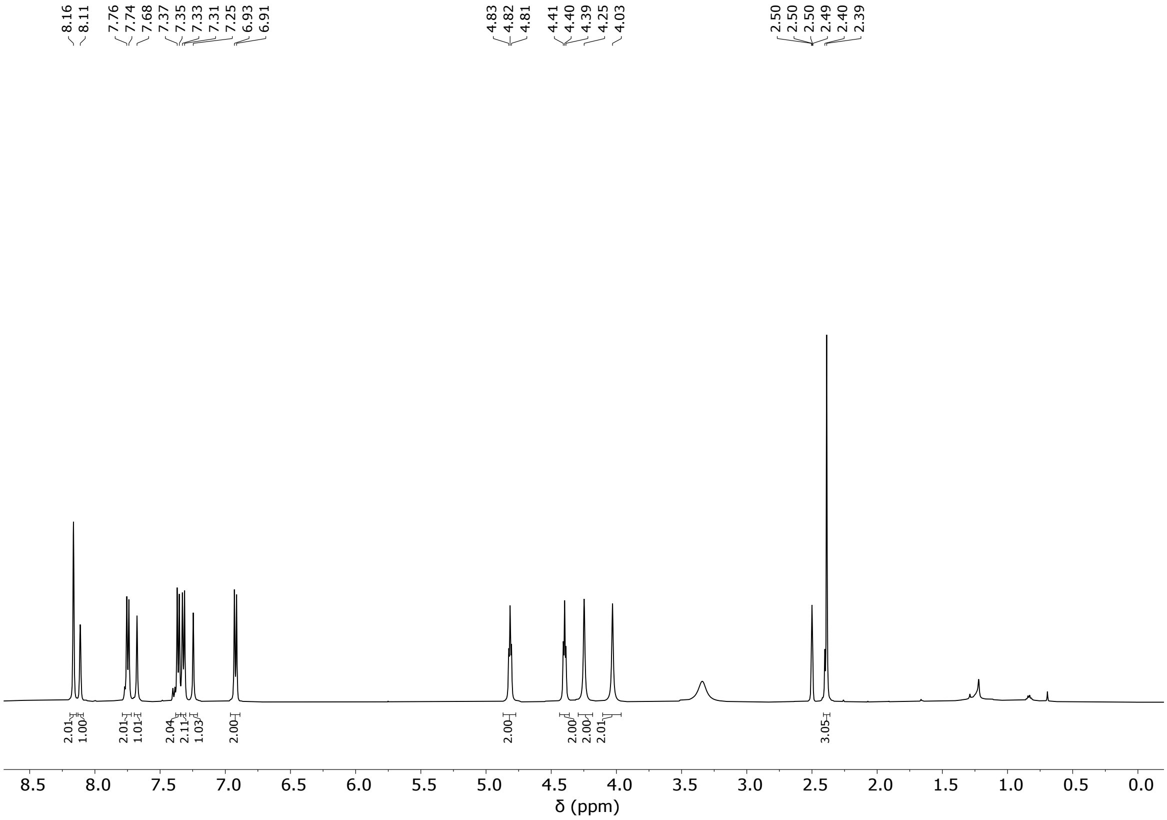


**Figure S17.** $^{1}H$ NMR spectrum of **6** (Chloroform-*d*, 298 K, 400 MHz).

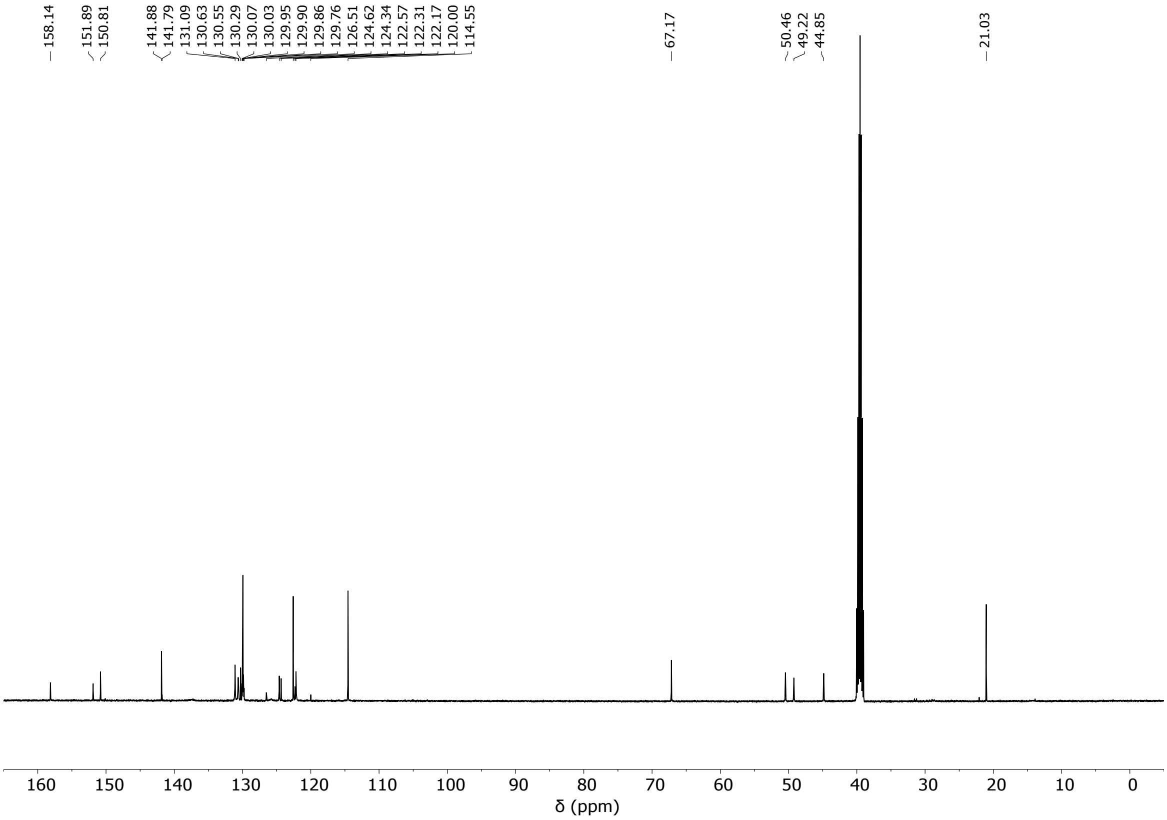


**Figure S18.** $^{13}C$ NMR spectrum of **6** (Chloroform-*d*, 298 K, 126 MHz).

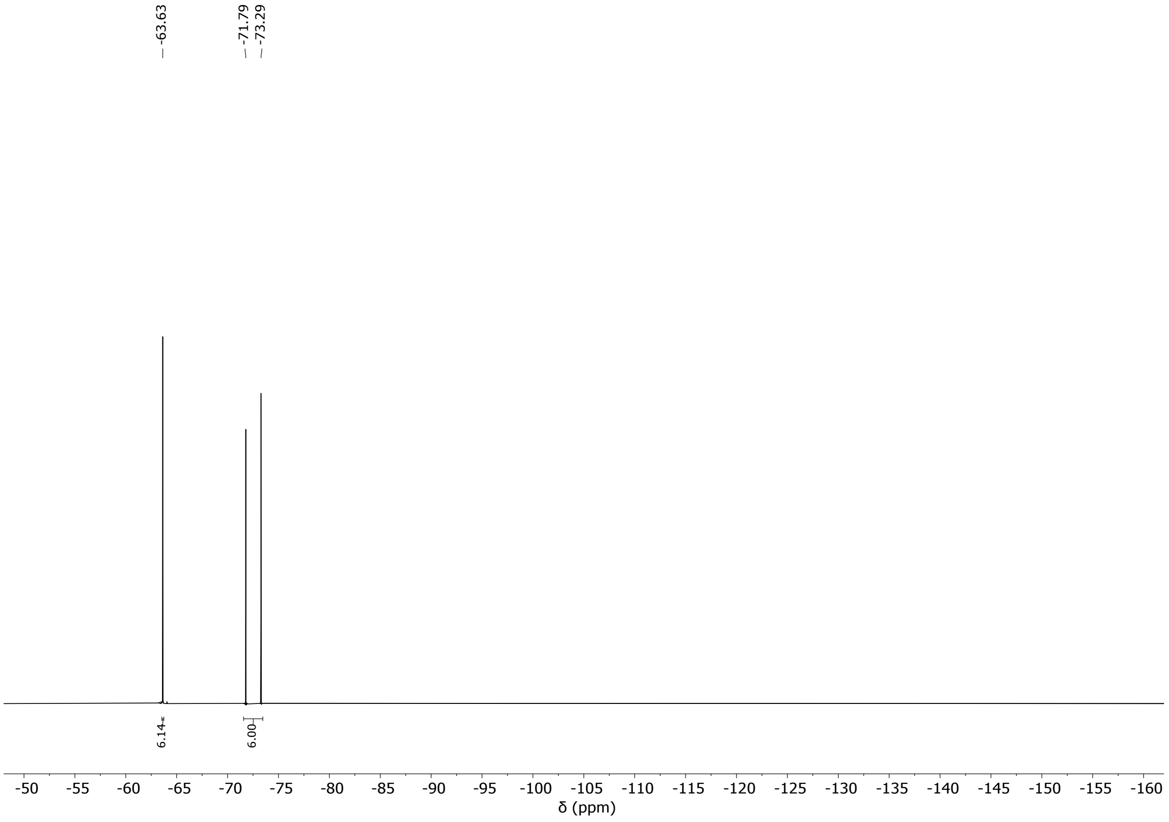

**Figure S19.** $^{19}F$ NMR spectrum of **6** (Chloroform-*d*, 298 K, 470 MHz).

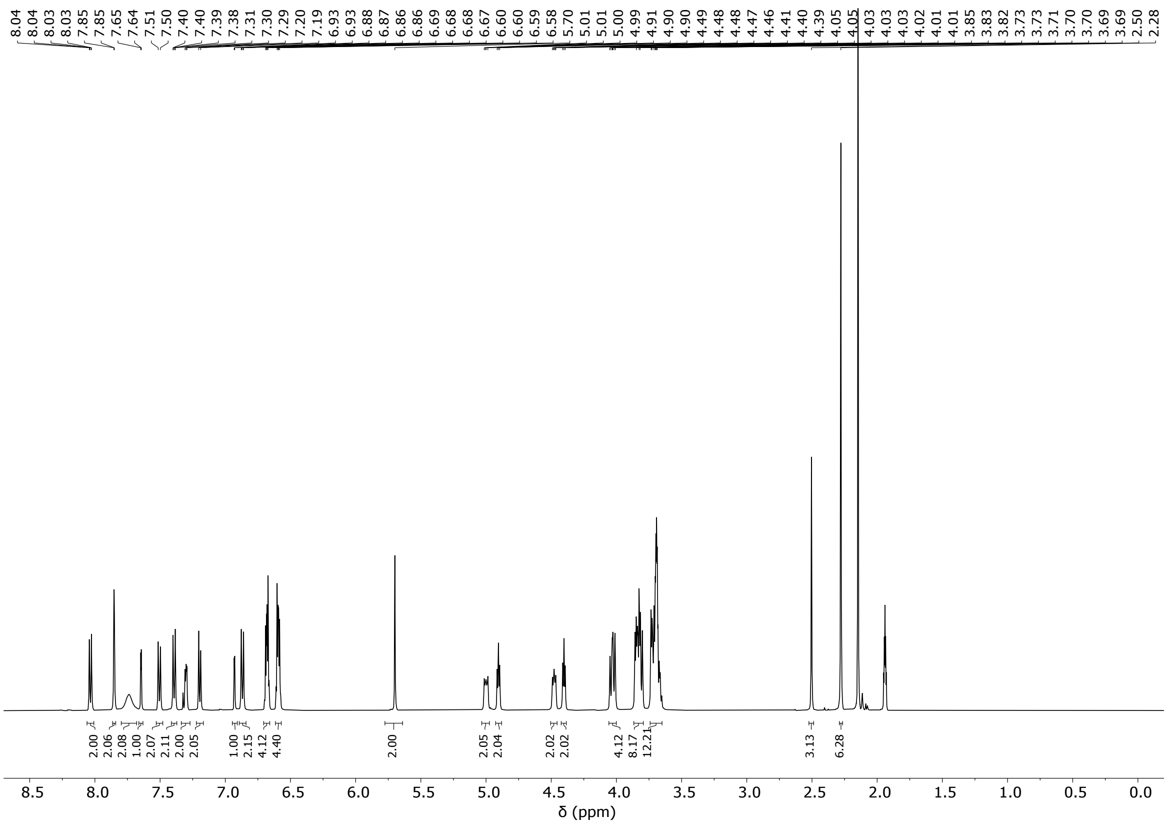

**Figure S20.** $^{1}H$ NMR spectrum of *E*-**1**$H^{2+}$ (Acetonitrile-$d_3$, 298 K, 500 MHz).

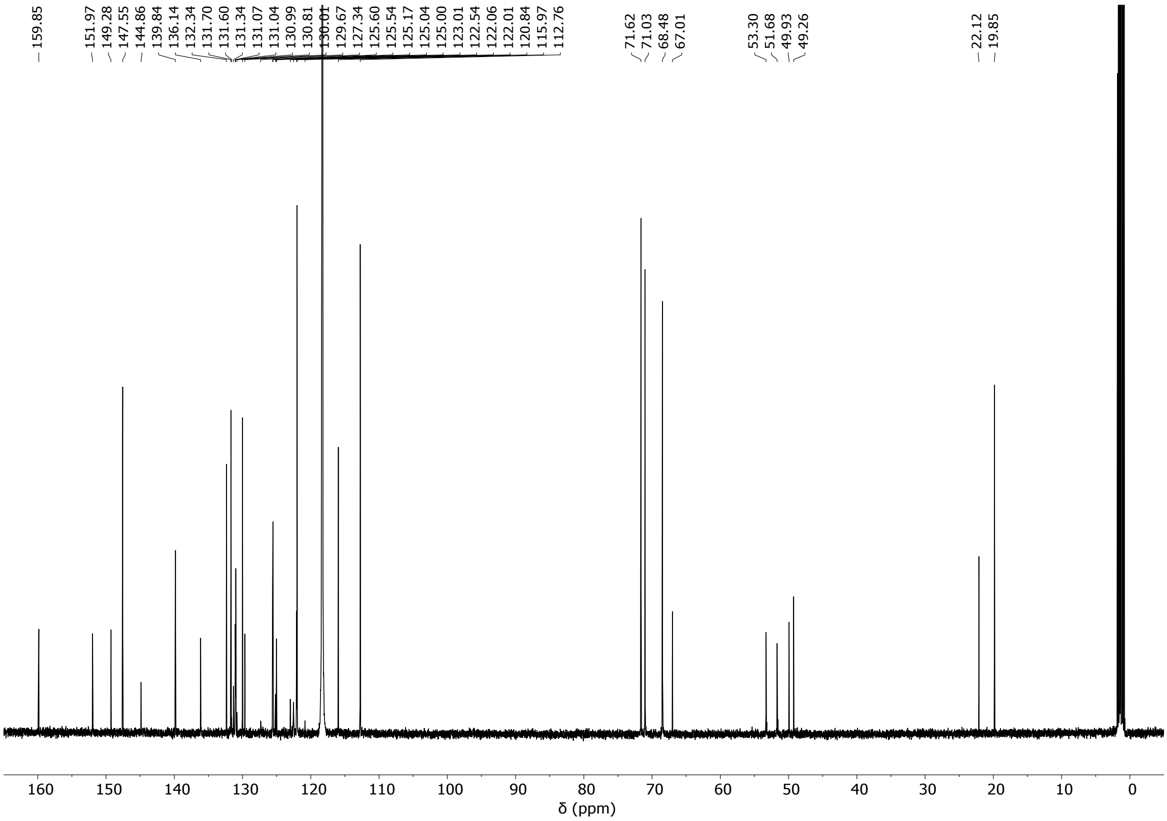


**Figure S21.** $^{13}$C NMR spectrum of *E*-**1**H$^{2+}$ (Acetonitrile-*d*$_3$, 298 K, 126 MHz).

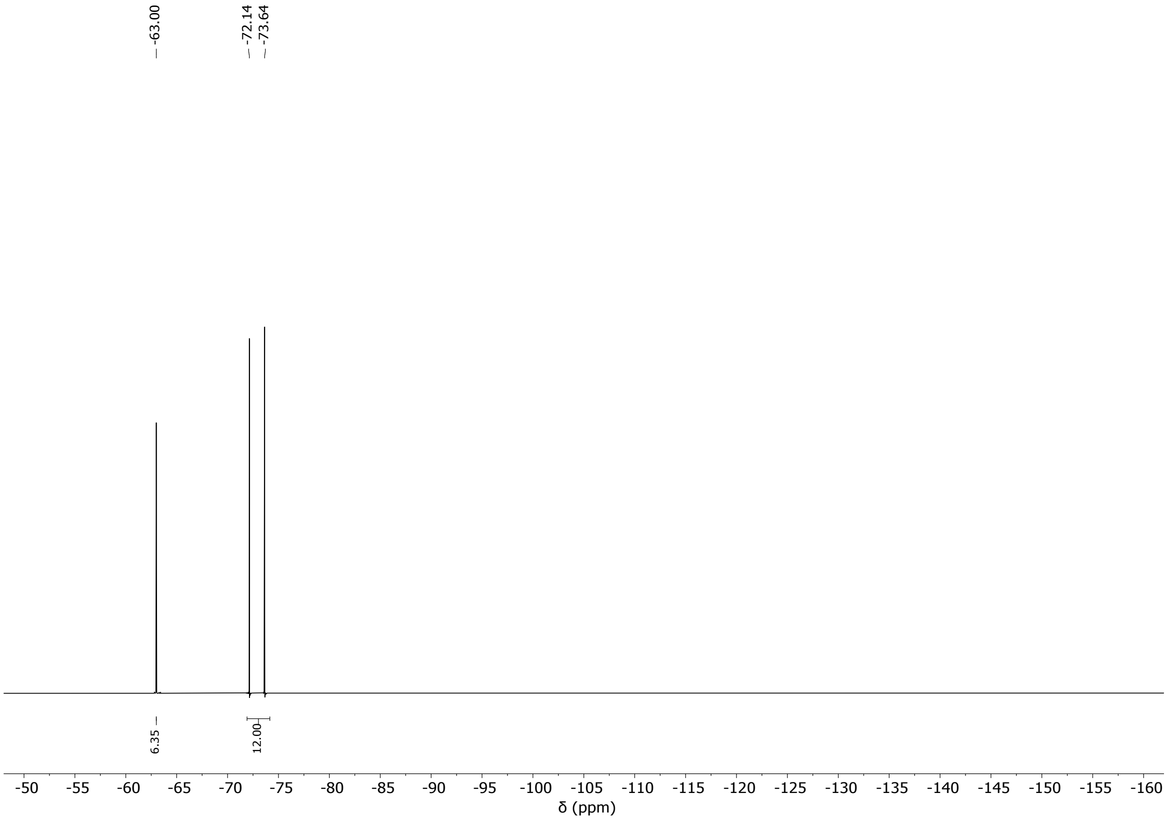


**Figure S22.** $^{19}$F NMR spectrum of *E*-**1**H$^{2+}$ (Acetonitrile-*d*$_3$, 298 K, 470 MHz).

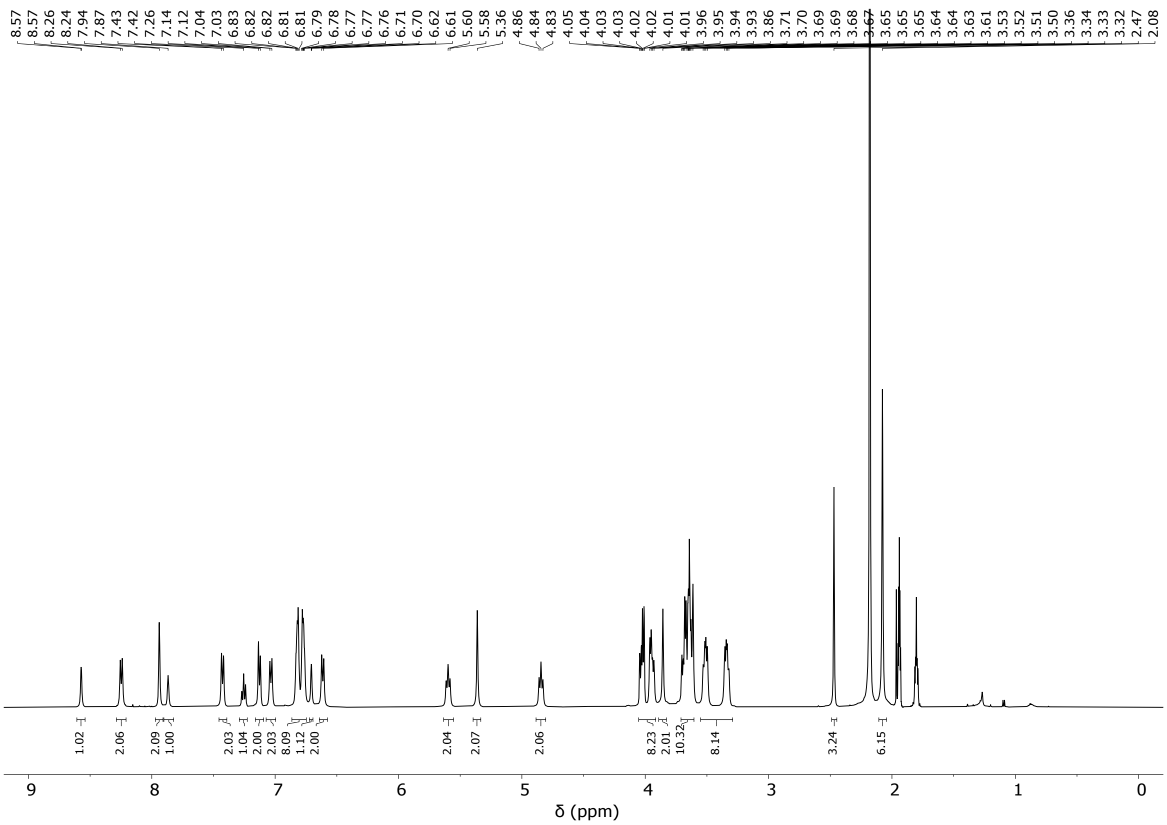


**Figure S23.** $^{1}$H NMR spectrum of *E*-**1**$^{+}$ (Acetonitrile-$d_3$, 298 K, 500 MHz).

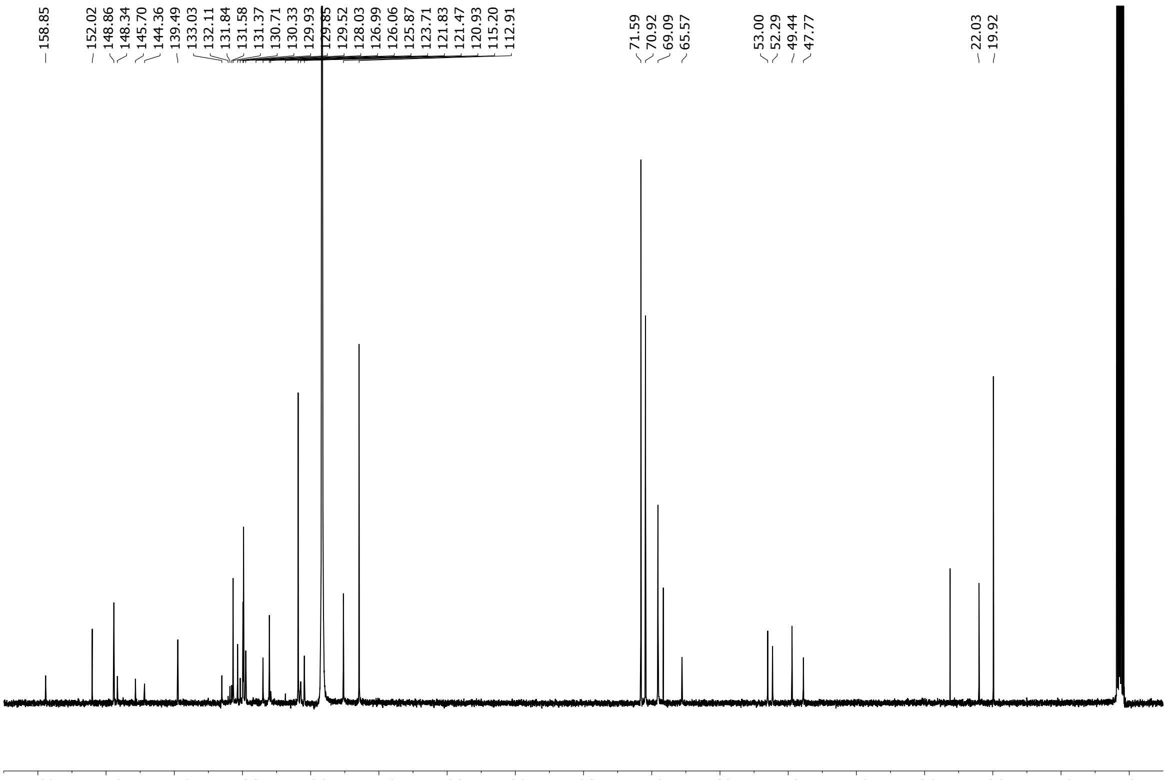


**Figure S24.** $^{13}$C NMR spectrum of *E*-**1**$^{+}$ (Acetonitrile-$d_3$, 298 K, 126 MHz).

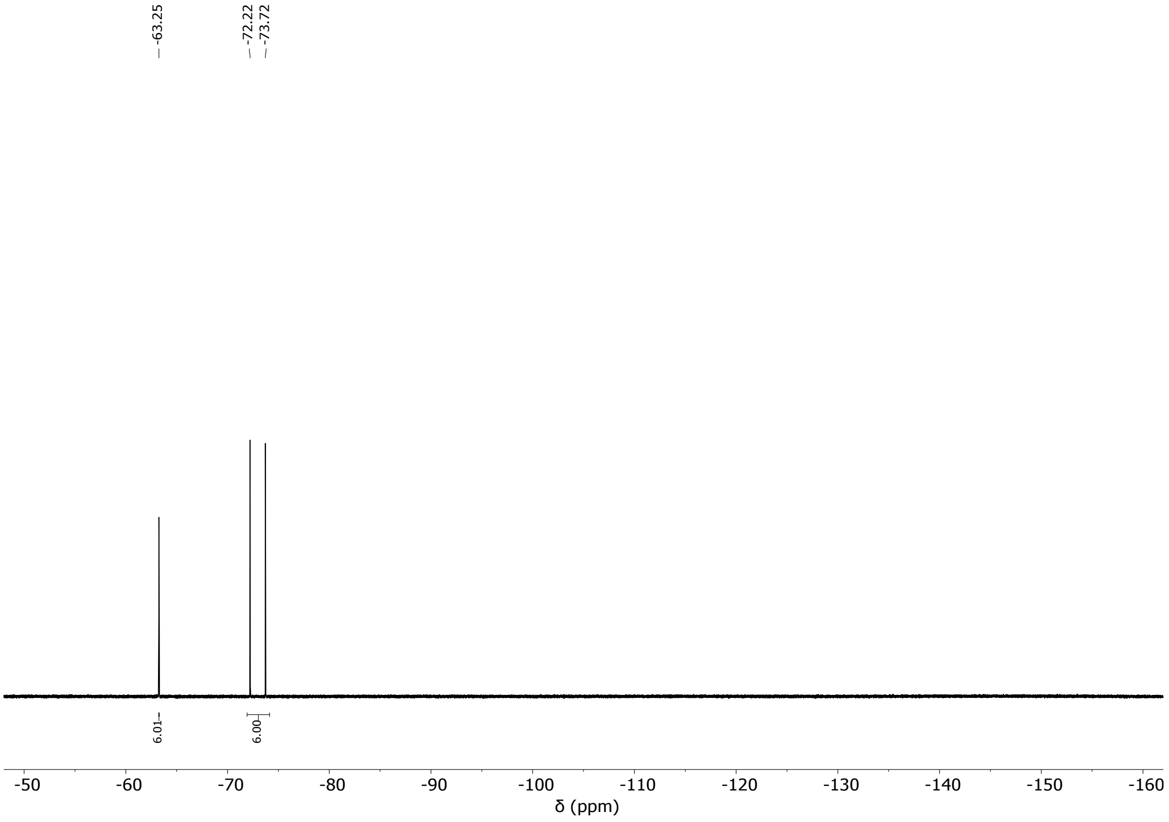


**Figure S25.** $^{19}$F NMR spectrum of *E*-**1**$^{+}$ (Acetonitrile-$d_3$, 298 K, 470 MHz).

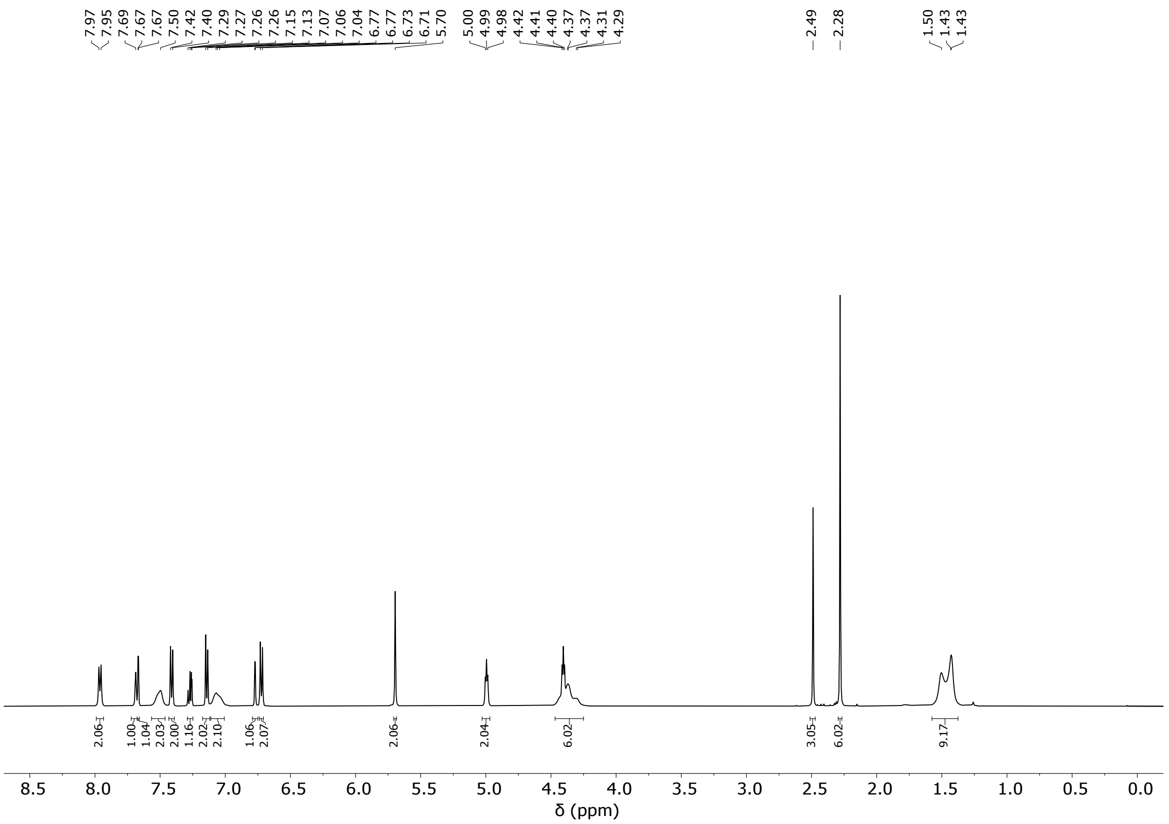


**Figure S26.** $^{1}$H NMR spectrum of **7** (Chloroform-*d*, 298 K, 400 MHz).

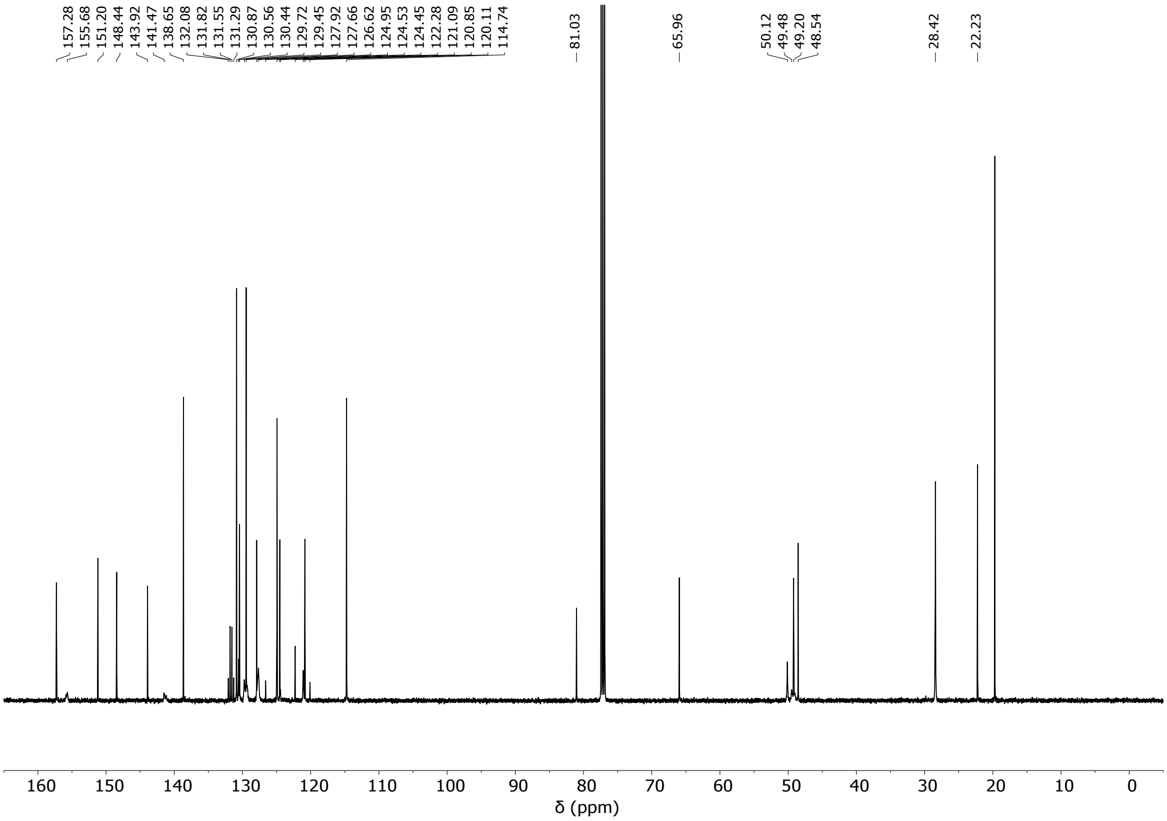


**Figure S27.** $^{13}$C NMR spectrum of **7** (Chloroform-*d*, 298 K, 126 MHz).

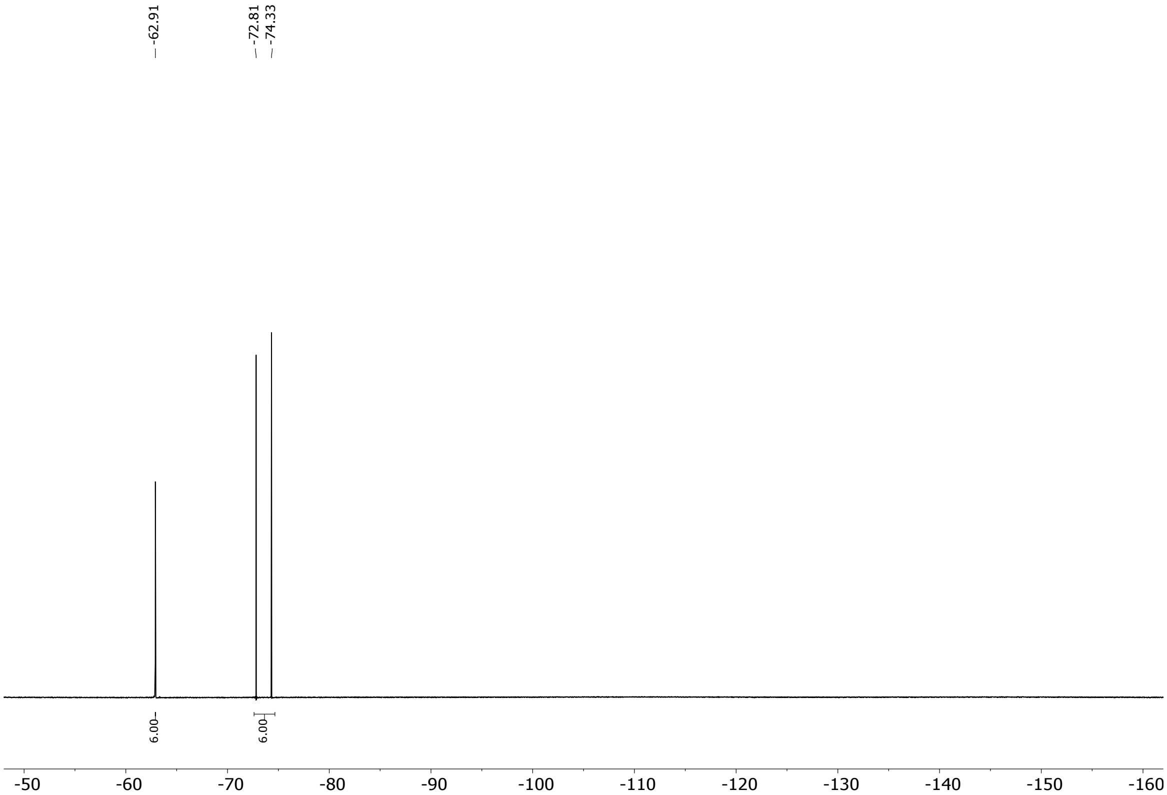


**Figure S28.** $^{19}$F NMR spectrum of **7** (Chloroform-*d*, 298 K, 470 MHz).

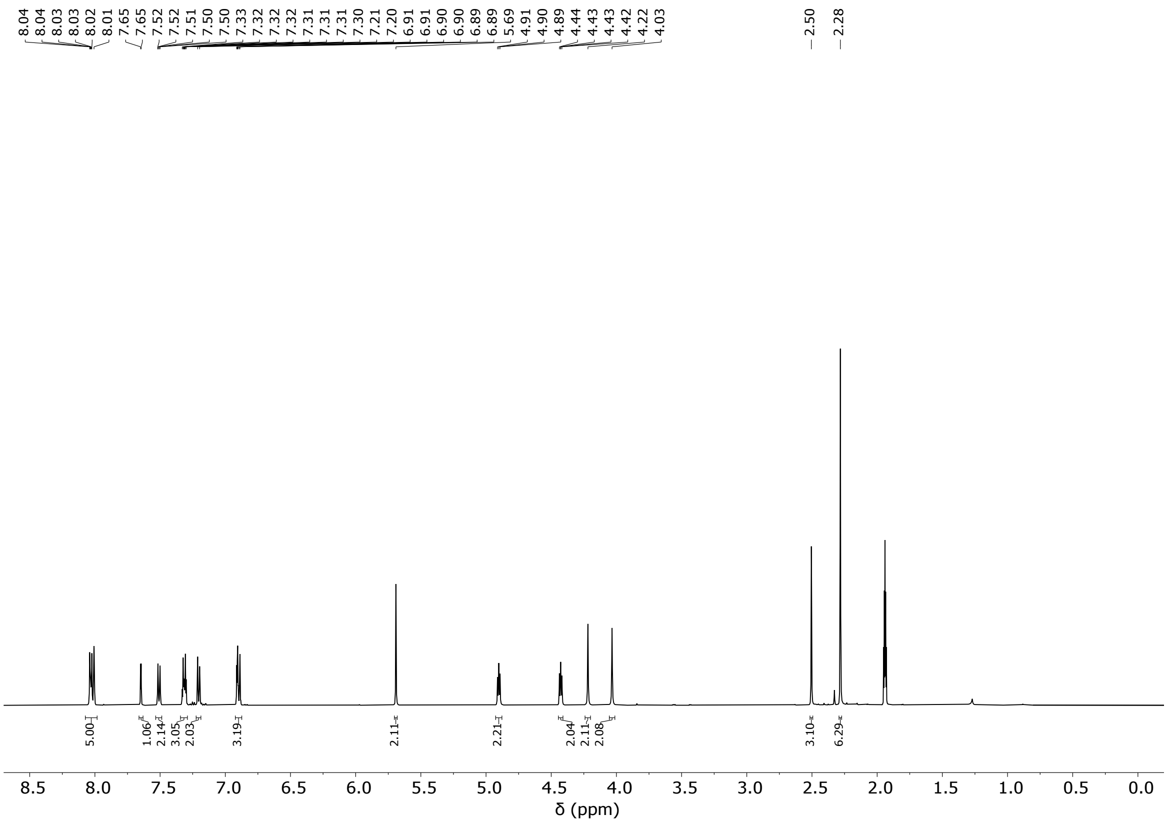


**Figure S29.** $^{1}$H NMR spectrum of *E*-**2**H$^{2+}$ (Acetonitrile-$d_3$, 298 K, 500 MHz).

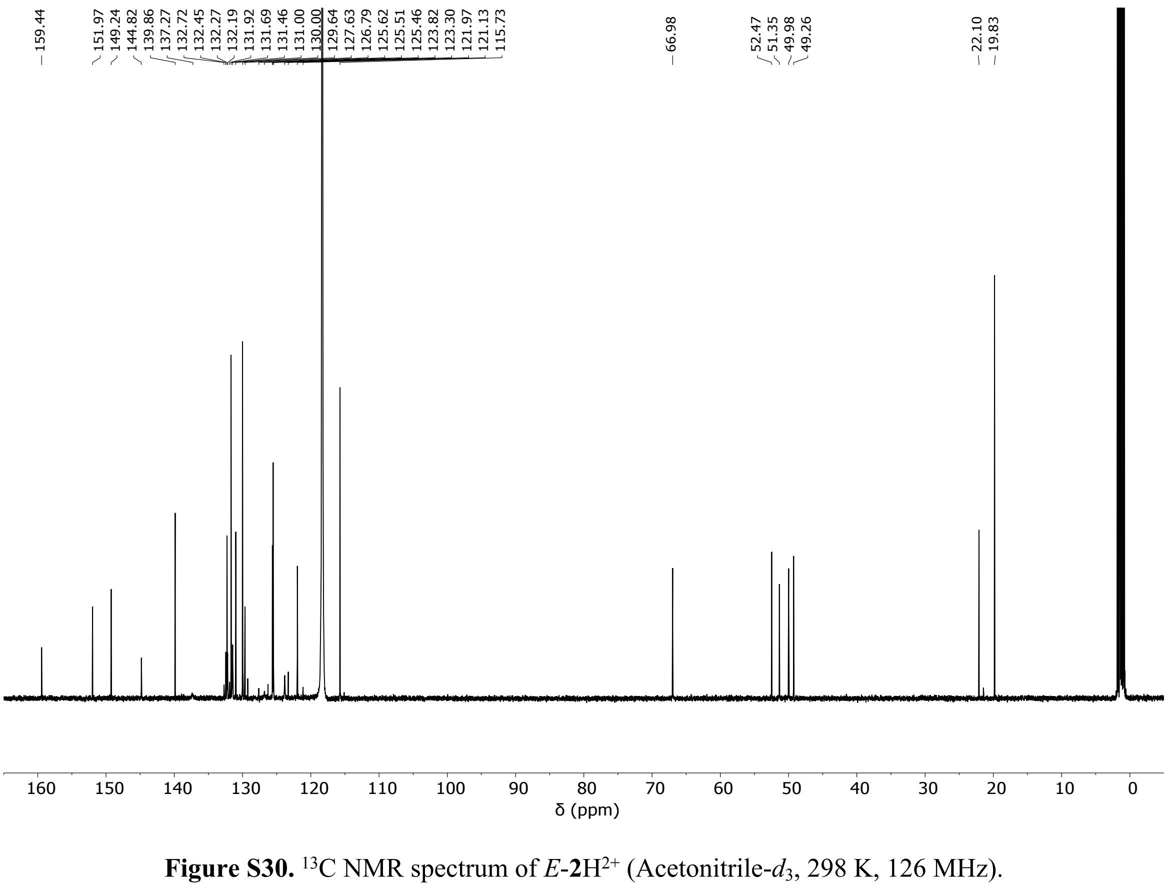


**Figure S30.** $^{13}$C NMR spectrum of *E*-**2**H$^{2+}$ (Acetonitrile-$d_3$, 298 K, 126 MHz).

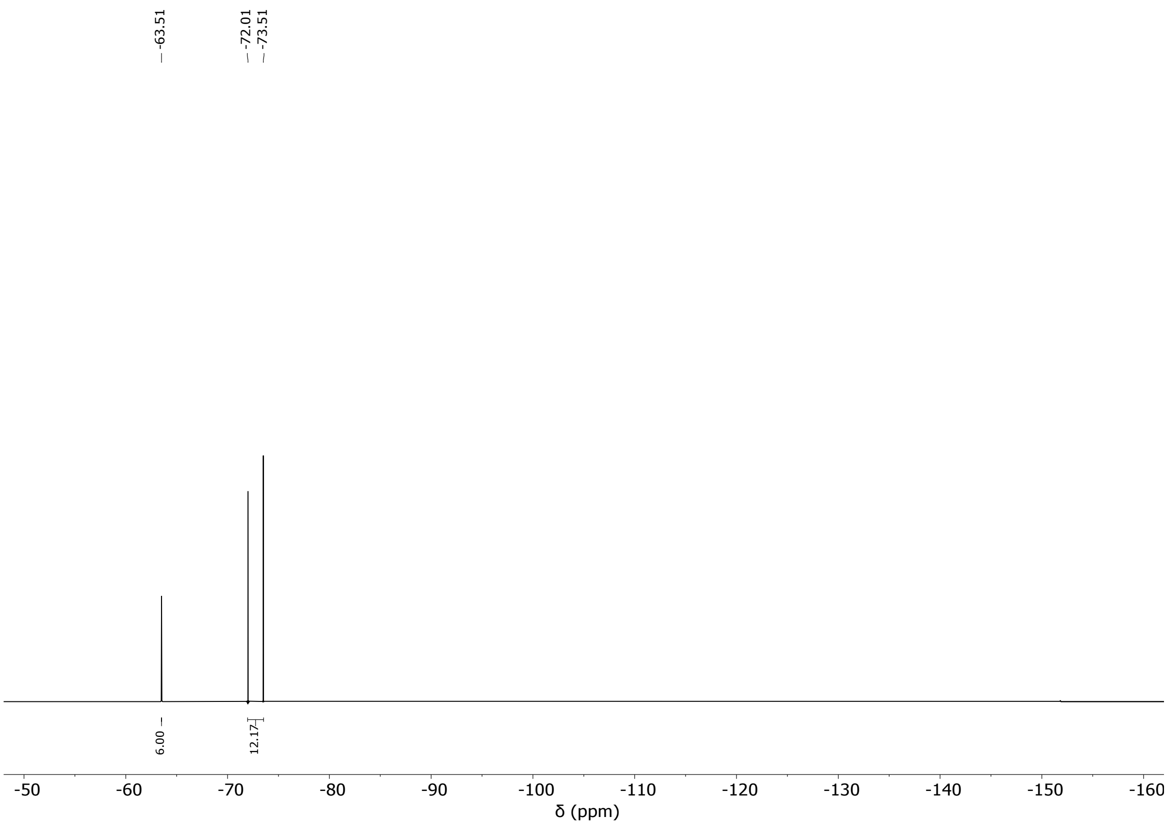

**Figure S31.** $^{19}F$ NMR spectrum of *E*-**2**$H^{2+}$ (Acetonitrile-$d_3$, 298 K, 470 MHz).

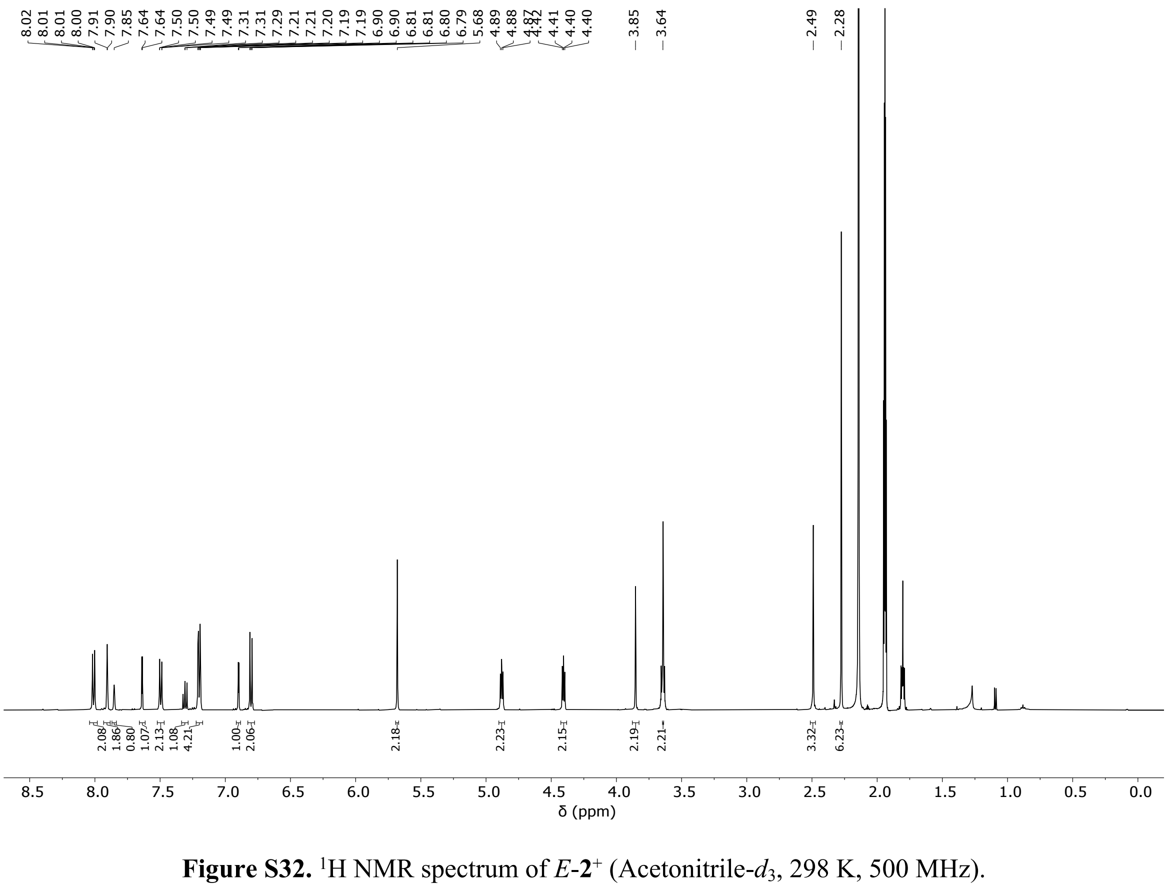

**Figure S32.** $^{1}H$ NMR spectrum of *E*-**2**$^{+}$ (Acetonitrile-$d_3$, 298 K, 500 MHz).

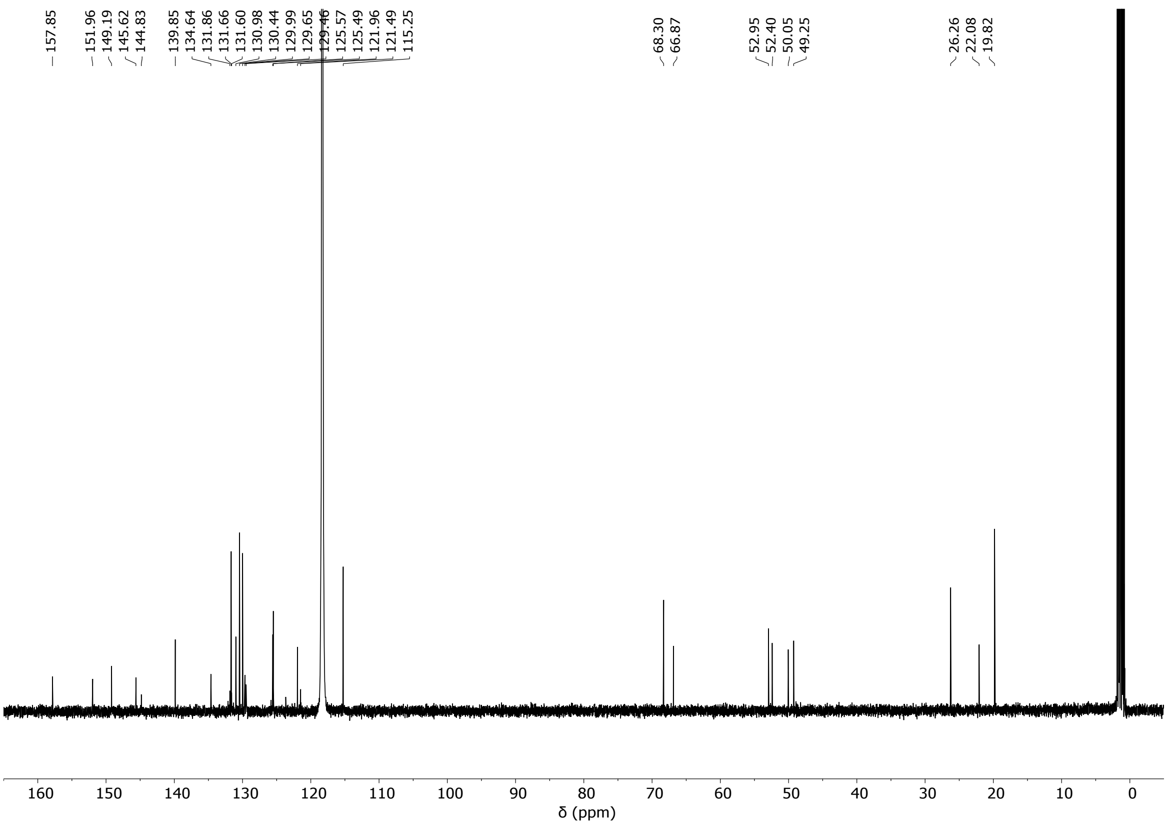


**Figure S33.** $^{13}$C NMR spectrum of *E*-$\mathbf{2}^{+}$ (Acetonitrile-$d_3$, 298 K, 126 MHz).

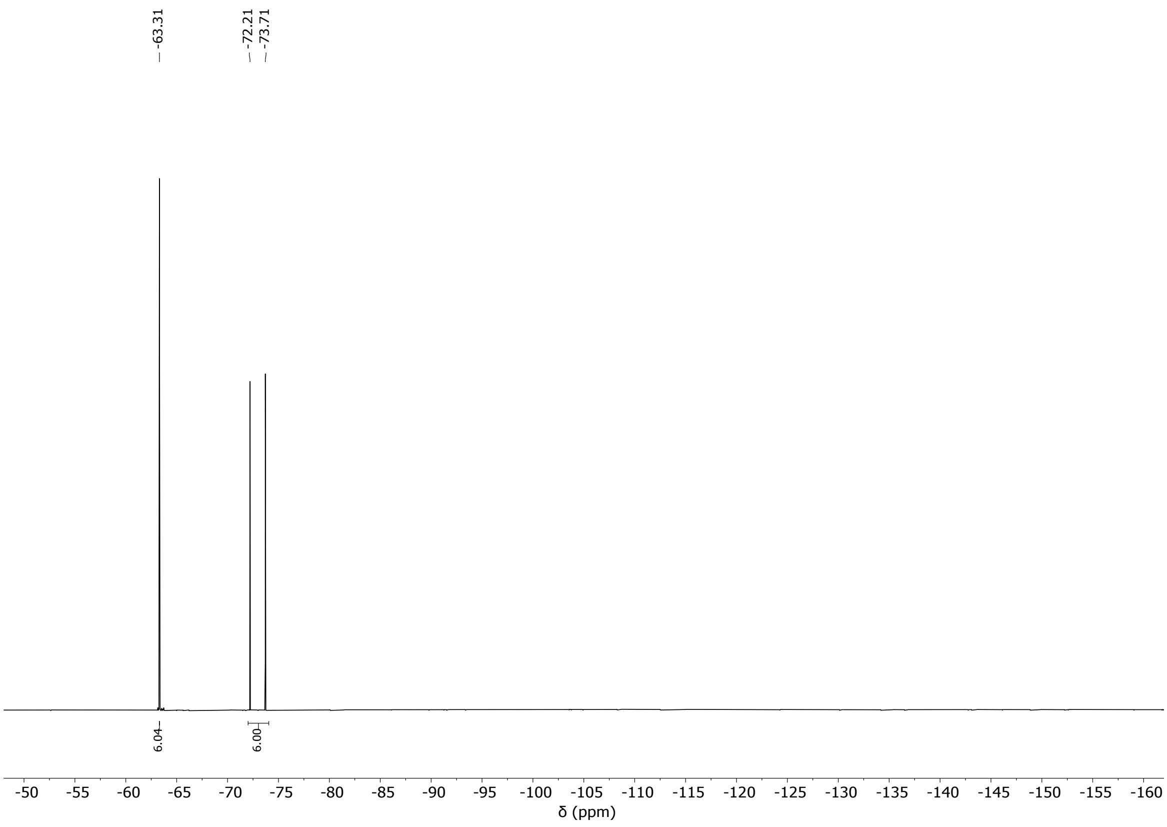


**Figure S34.** $^{19}$F NMR spectrum of *E*-$\mathbf{2}^{+}$ (Acetonitrile-$d_3$, 298 K, 470 MHz).

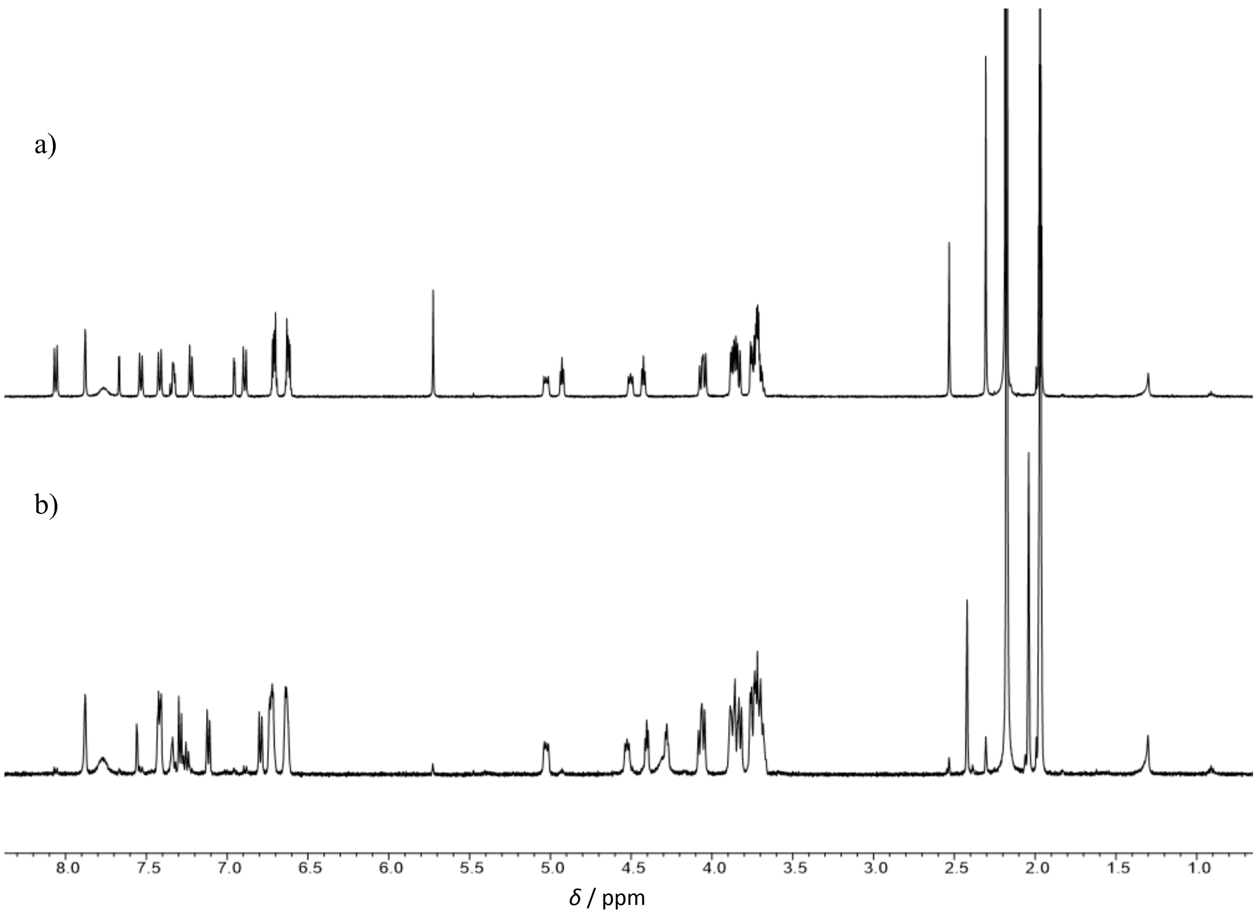


**Figure S35.** $^{1}$H NMR spectrum of *E*-**1**H$^{2+}$ a) before and b) upon reaching the photostationary state; $c = 5.0 \times 10^{-3}$ mol dm$^{-3}$, $\lambda_{irr}$ = 365 nm, Acetonitrile-$d_3$, 298 K, 500 MHz.

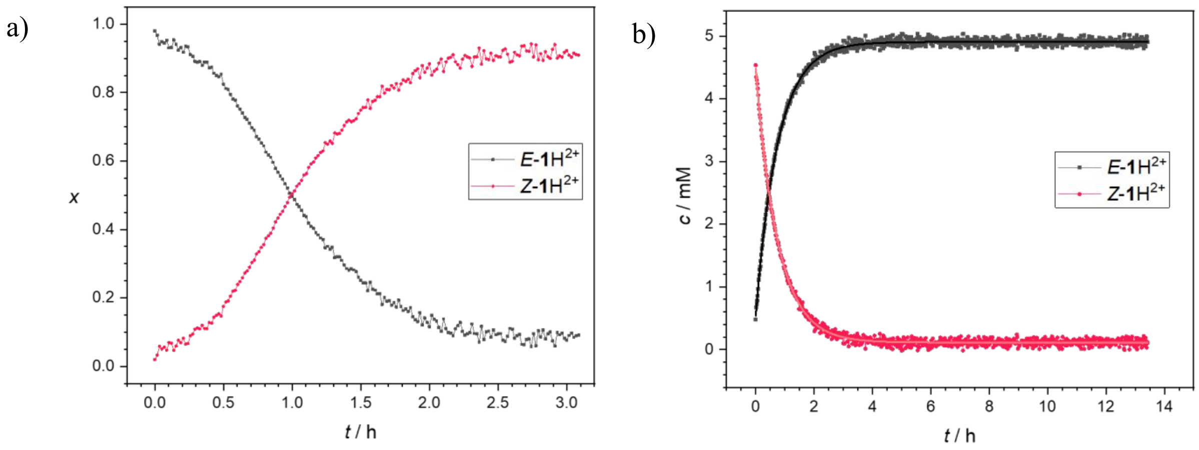


**Figure S36.** Kinetic profile of a) the isomerization process of *E*-**1**H$^{2+}$ and b) the back-isomerization process of *Z*-**1**H$^{2+}$ ( ■ experimental data, – calculated data); $c = 5.0 \times 10^{-3}$ mol dm$^{-3}$, $\lambda_{irr}$ = 365 nm, Acetonitrile-$d_3$, 298 K, 500 MHz.

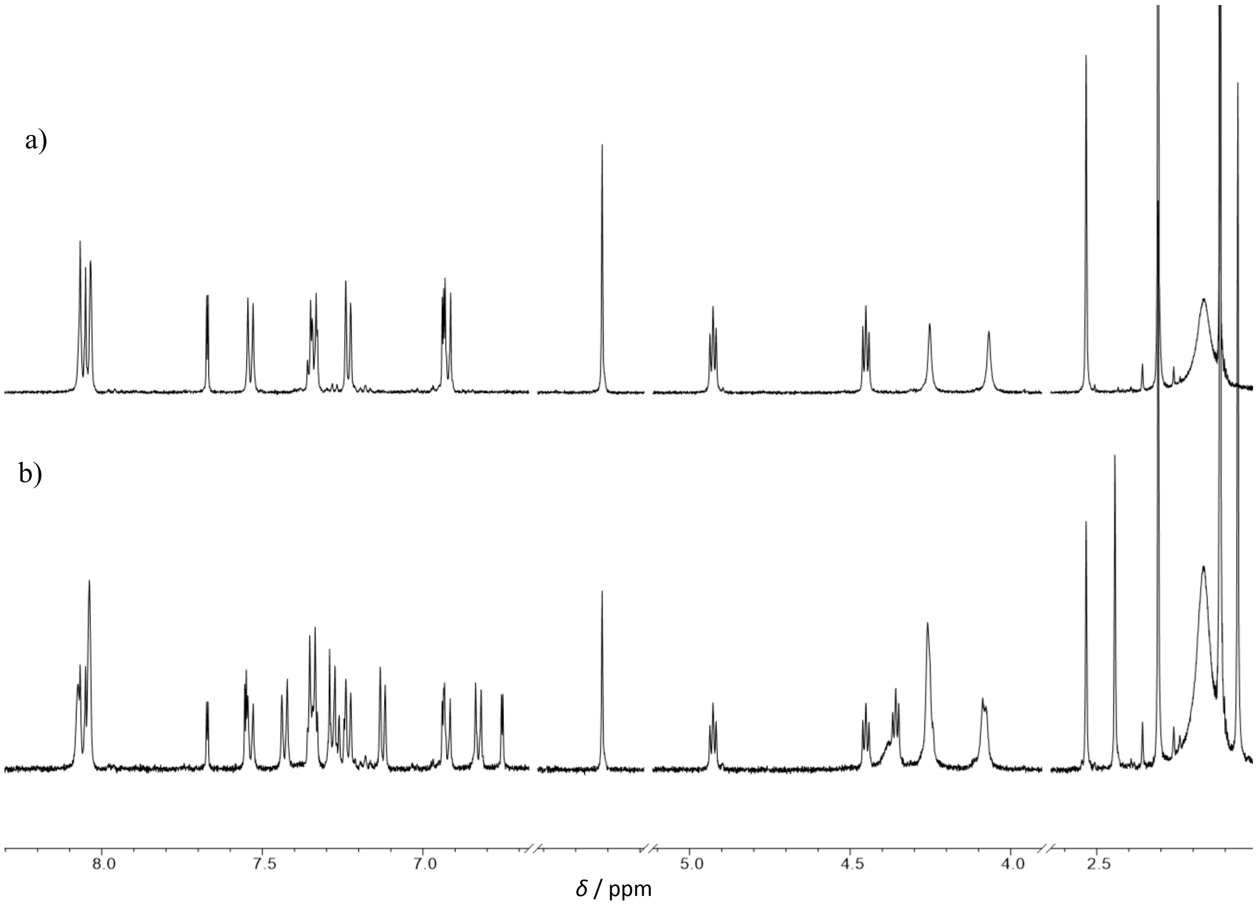


**Figure S37.** $^{1}$H NMR spectrum of $E$-**2**H$^{2+}$ a) before and b) upon reaching the photostationary state; $c = 5.0 \times 10^{-3}$ mol dm$^{-3}$, $\lambda_{irr}$ = 365 nm, Acetonitrile-$d_3$, 298 K, 500 MHz.

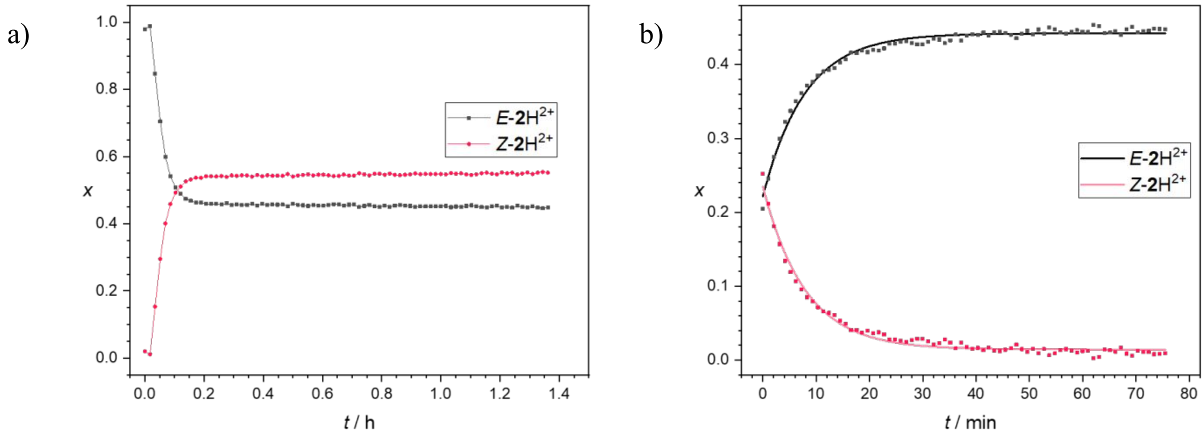


**Figure S38.** Kinetic profile of a) the isomerization process of $E$-**2**H$^{2+}$ and b) the back-isomerization process of $Z$-**2**H$^{2+}$ ( ■ experimental data, – calculated data); $c = 5.0 \times 10^{-3}$ mol dm$^{-3}$, $\lambda_{irr}$ = 365 nm, Acetonitrile-$d_3$, 298 K, 500 MHz.

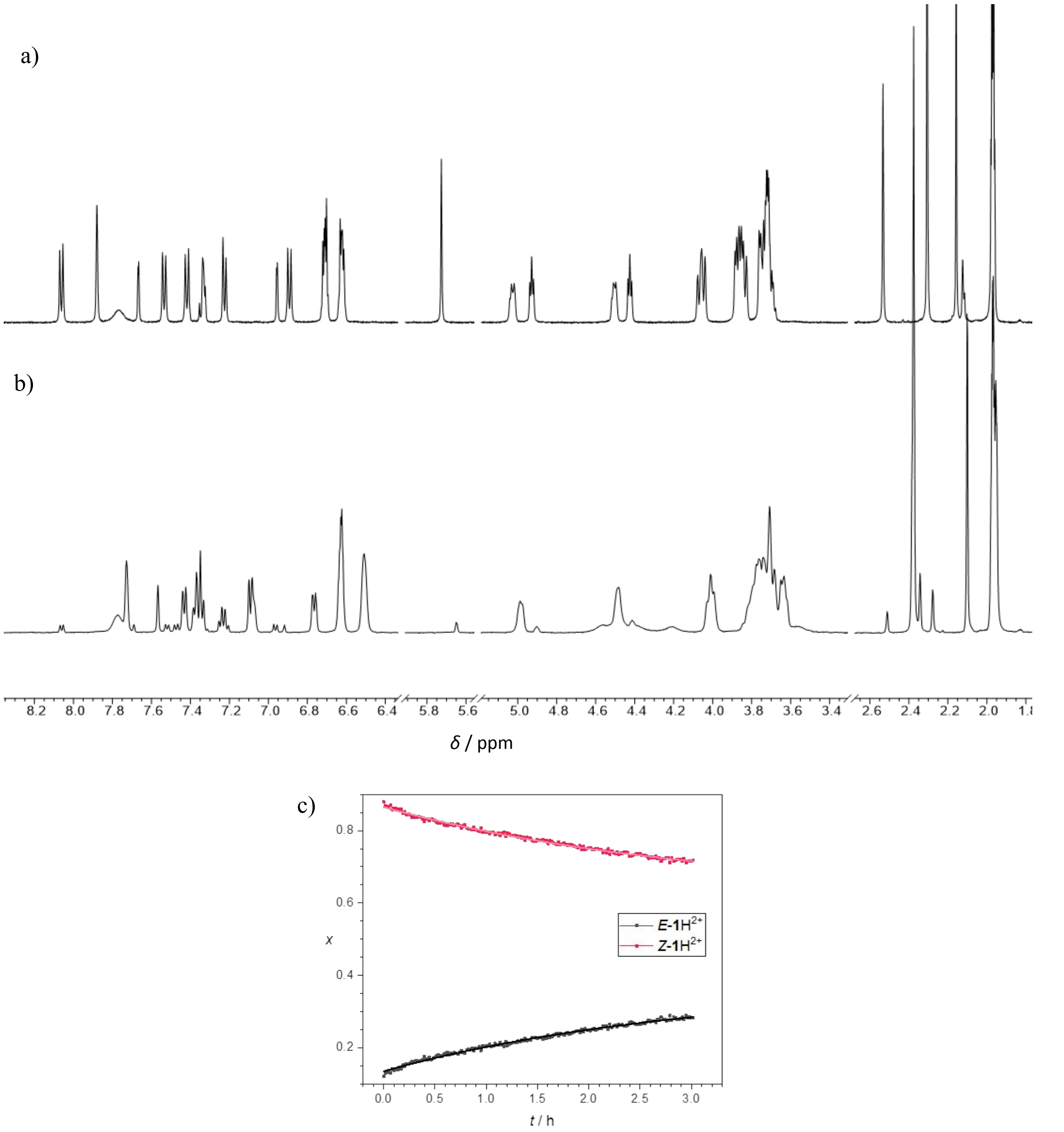


**Figure S39.** $^{1}$H NMR spectrum of *E*-**1**$H^{2+}$ a) before and b) upon reaching the photostationary state and c) kinetic profile of the back-isomerization process of *Z*-**1**$H^{2+}$ ( ■ experimental data, – calculated data); $c = 5.0 \times 10^{-3}$ mol dm$^{-3}$, $\lambda_{irr} = 365$ nm, Acetonitrile-$d_3$, 238 K, 500 MHz.

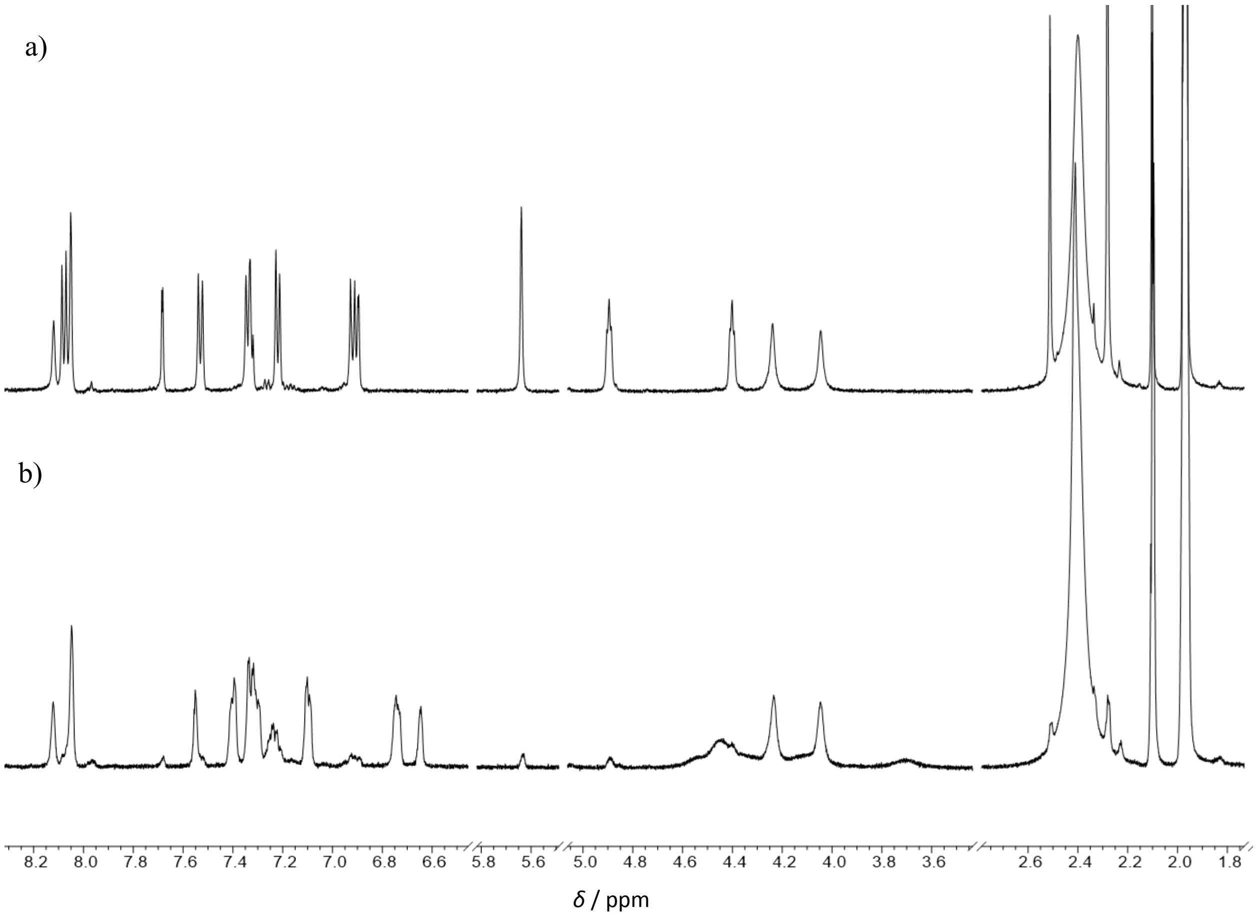


**Figure S40.** $^1$H NMR spectrum of *E*-**2**$H^{2+}$ a) before and b) upon reaching the photostationary state; $c = 5.0 \times 10^{-3}$ mol dm$^{-3}$, $\lambda_{irr}$ = 365 nm, Acetonitrile-$d_3$, 238 K, 500 MHz.

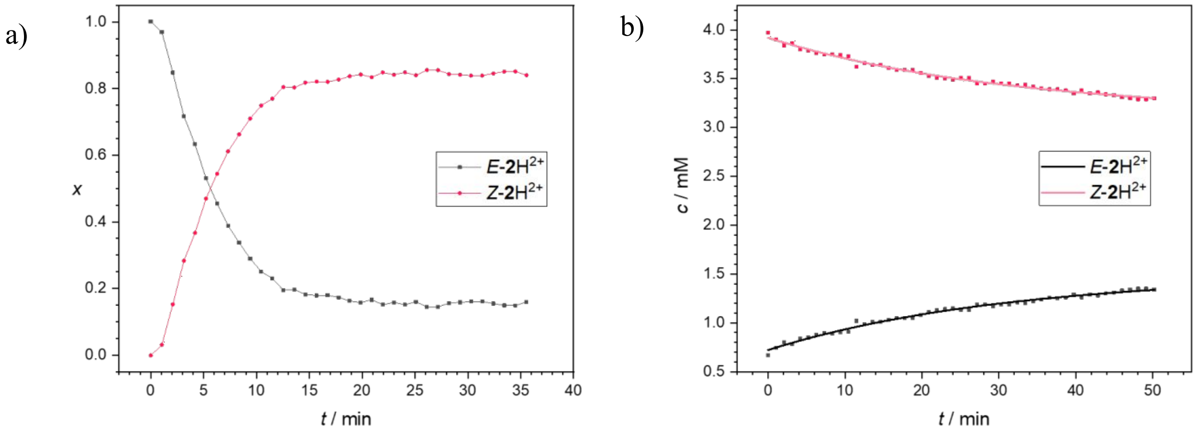


**Figure S41.** Kinetic profile of a) the isomerization process of *E*-**2**$H^{2+}$ and b) the back-isomerization process of *Z*-**2**$H^{2+}$; $c = 5.0 \times 10^{-3}$ mol dm$^{-3}$, $\lambda_{irr}$ = 365 nm, Acetonitrile-$d_3$, 238 K, 500 MHz.

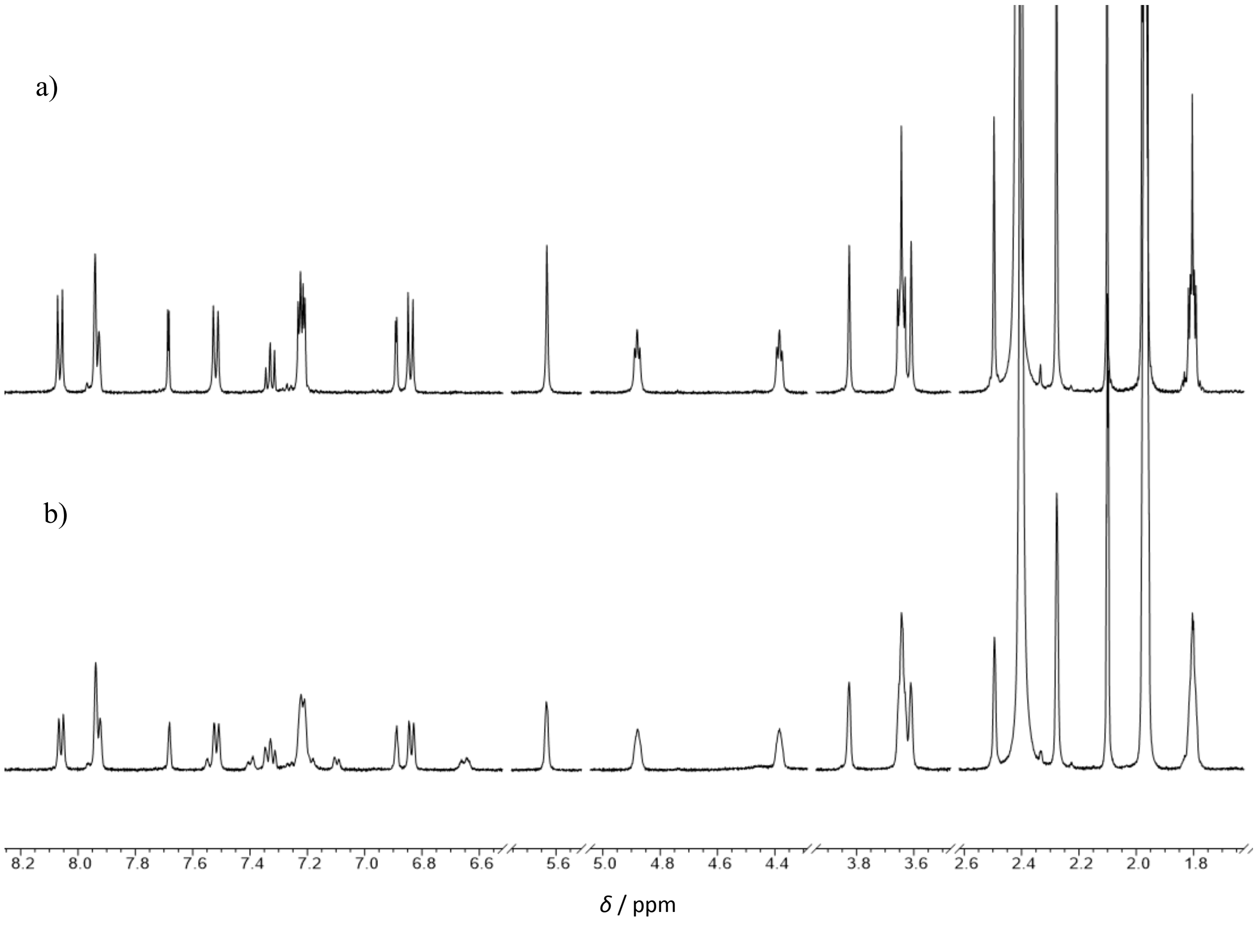


**Figure S42.** $^{1}$H NMR spectrum of $E$-**2**$^{+}$ a) before and b) upon reaching the photostationary state; $c = 5.0 \times 10^{-3}$ mol dm$^{-3}$, $\lambda_{irr}$ = 365 nm, Acetonitrile-$d_3$, 238 K, 500 MHz.

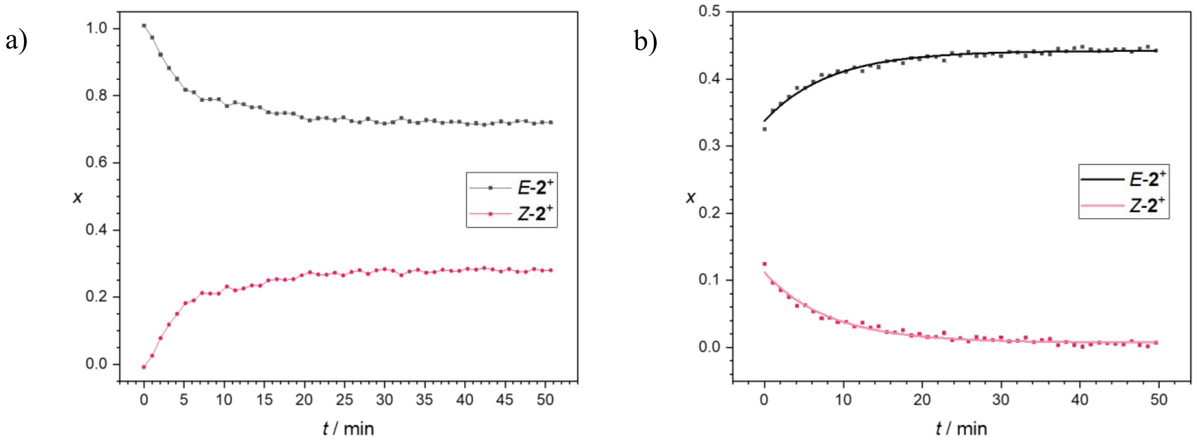


**Figure S43.** Kinetic profile of a) the isomerization process of $E$-**2**$^{+}$ and b) the back-isomerization process of $Z$-**2**$^{+}$; $c = 5.0 \times 10^{-3}$ mol dm$^{-3}$, $\lambda_{irr}$ = 365 nm, Acetonitrile-$d_3$, 238 K, 500 MHz. Experimental data for this compound suggest that the back-isomerization process is influenced by a concentration-dependent phenomenon, such as aggregation.

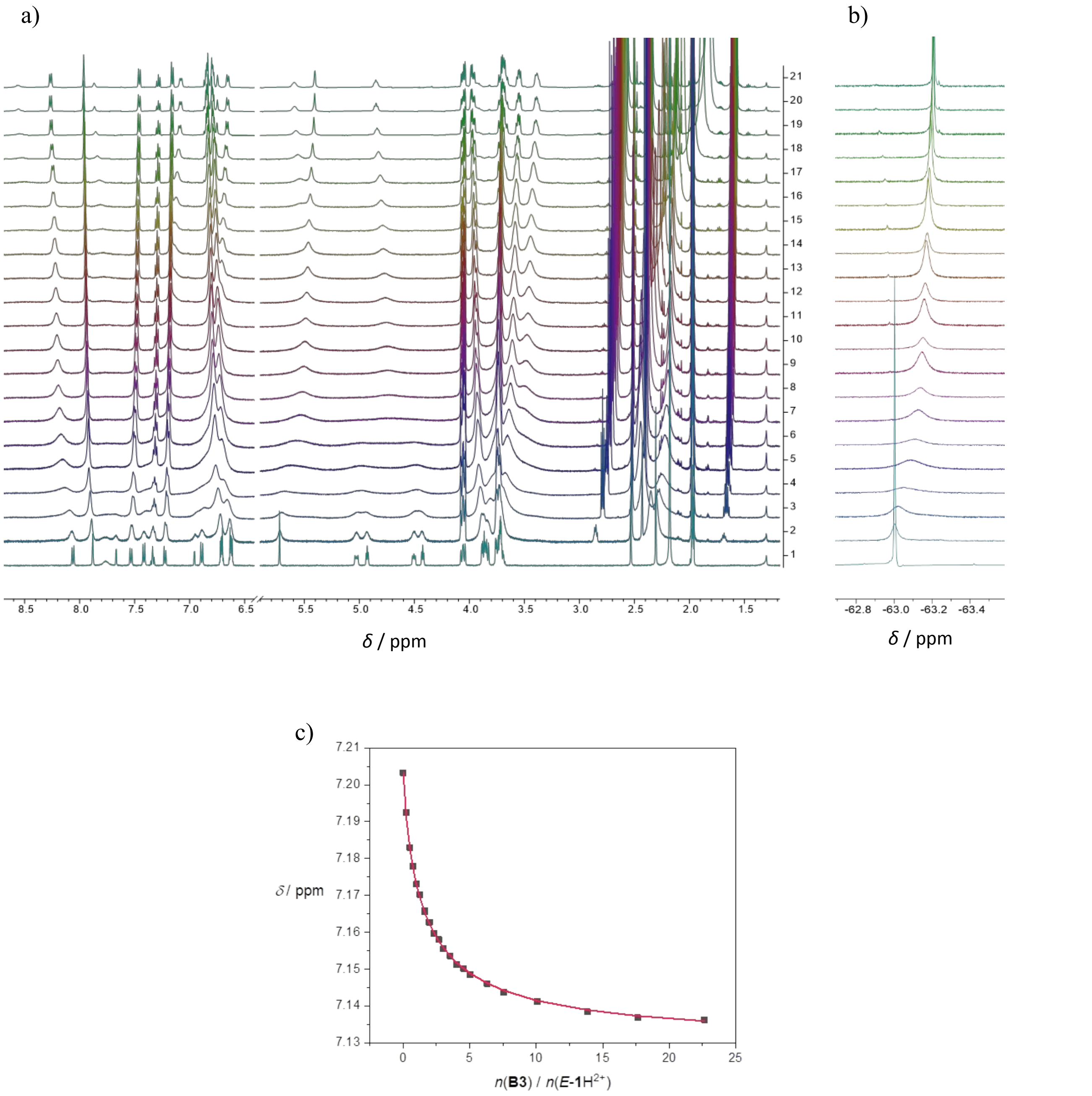


**Figure S44.** a) $^{1}$H and b) $^{19}$F NMR spectral changes of *E*-**1**H$^{2+}$ upon titration with base **B3** (*n*(**B3**/ *E*-**1**H$^{2+}$) = 0.00 (1), 0.25 (2), 0.50 (3), 0.76 (4), 1.01 (5), 1.26 (6), 1.61 (7), 1.96 (8), 2.32 (9), 2.67 (10), 3.02 (11), 3.52 (12), 4.03 (13), 4.53 (14), 5.04 (15), 6.29 (16), 7.55 (17), 10.07 (18), 13.85 (19), 17.62 (20), 22.66 (21)) c) titration curve obtained by integration of peaks in the $^{19}$F NMR spectra; Acetonitrile-$d_3$, 298 K, 500 MHz ($^{1}$H) or 470 MHz ($^{19}$F).

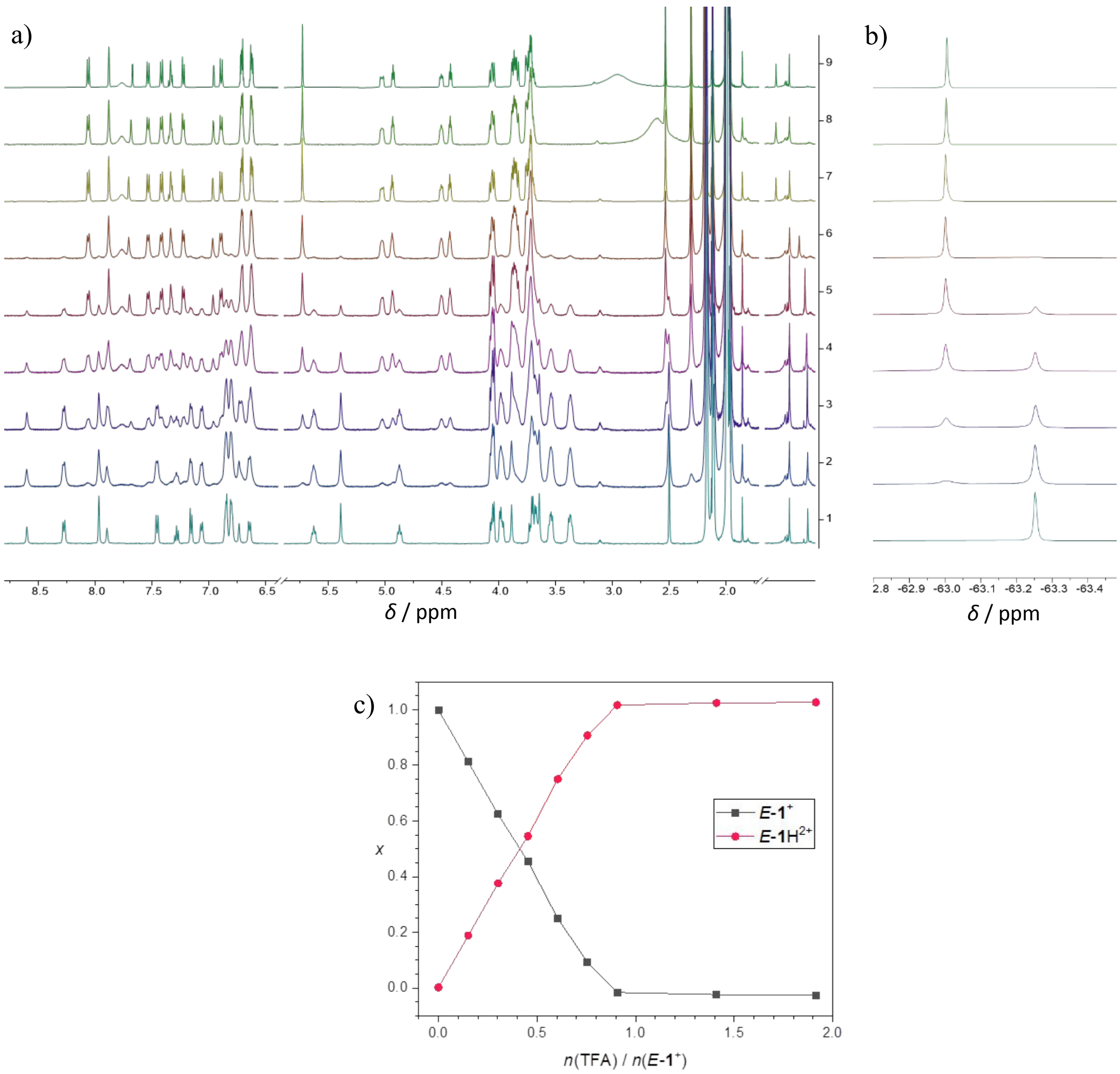


**Figure S45.** a) $^{1}$H and b) $^{19}$F NMR spectral changes of *E*-**1**$^{+}$ upon titration with trifluoroacetic acid (*n*(TFA/ *E*-**1**$^{+}$) = 0.00 (1), 0.15 (2),.0.30 (3), 0.45 (4), 0.60 (5), 0.76 (6), 0.91 (7), 1.41 (8), 1.91 (9)) c) titration curve obtained by integration of peaks in $^{19}$F NMR spectra; Acetonitrile-$d_3$, 298 K, 500 MHz ($^{1}$H) or 470 MHz ($^{19}$F).

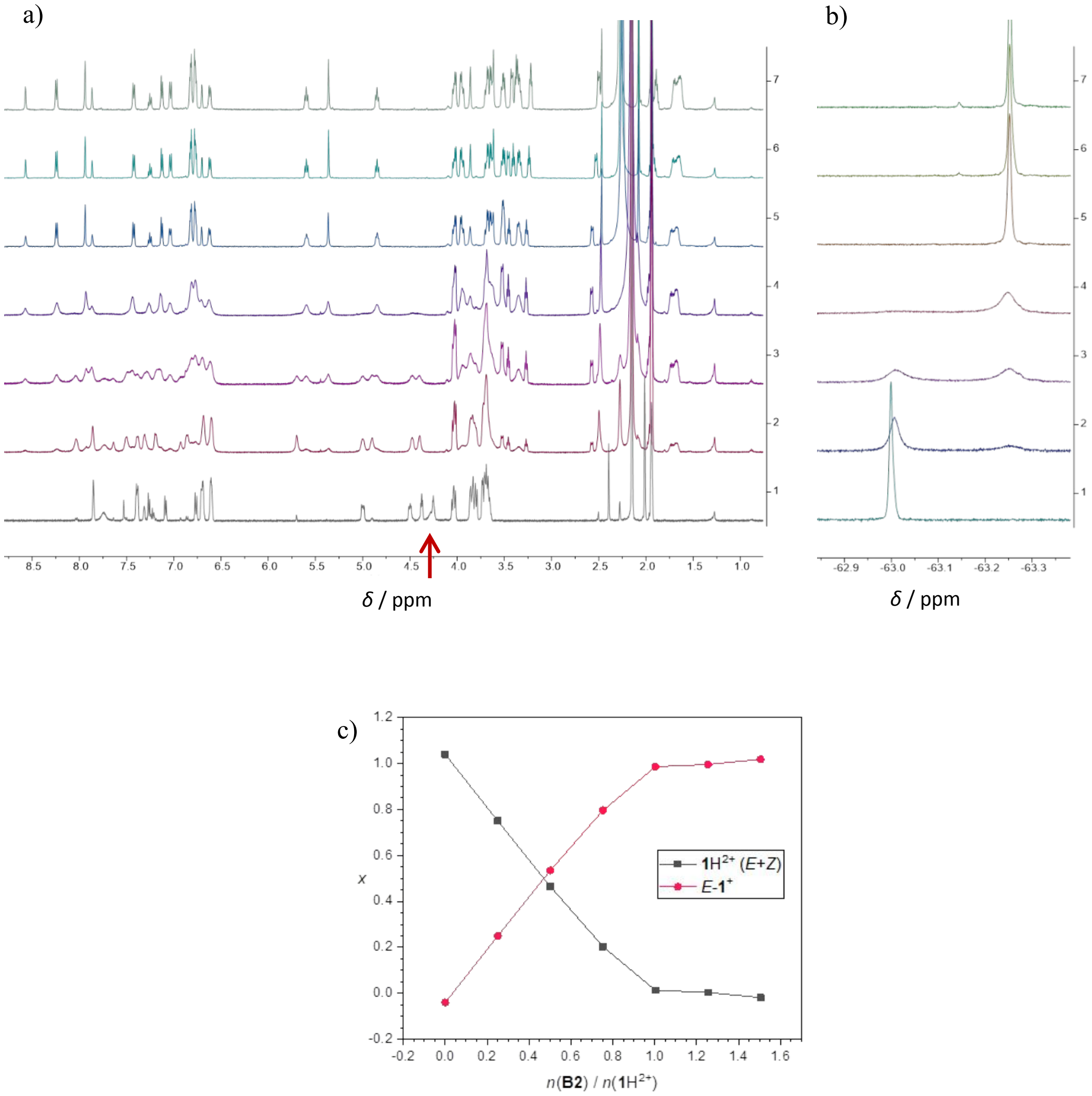


**Figure S46.** a) $^{1}$H and b) $^{19}$F NMR spectral changes of *Z*-**1**H$^{2+}$ upon titration with base **B2** under continuous irradiation ($n$(**B2**/*Z*-**1**H$^{2+}$) = 0.00 (1), 0.25 (2),.0.50 (3), 0.75 (4), 1.00 (5), 1.25 (6), 1.50 (7)) c) titration curve obtained by integration of peaks in $^{19}$F NMR spectra; Acetonitrile-$d_3$, 298 K, 500 MHz ($^{1}$H) or 470 MHz ($^{19}$F). During the $^{1}$H NMR titration (a) the signal corresponding to *Z*-**1**H$^{2+}$ (4.25 ppm, red arrow) disappears completely after the first addition. The peaks in the first and last $^{19}$F NMR spectra (b) refer to the complexation state of the ammonium station: complexed station resonance at –63.0 ppm, free station resonance at –63.3 ppm.

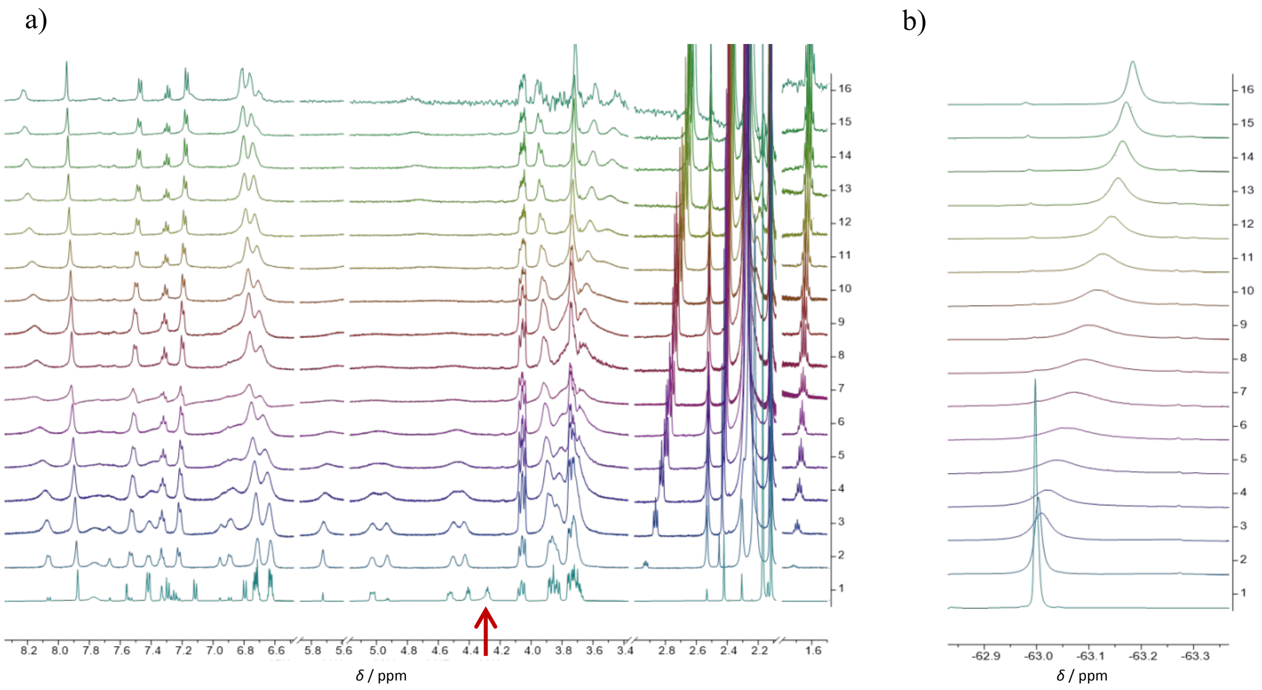


**Figure S47.** a) $^1$H and b) $^{19}$F NMR spectral variations of $Z$-**1**H$^{2+}$ upon titration with base **B3** under continuous irradiation ($n$(**B3**/$Z$-**1**H$^{2+}$) = 0.00 (1), 0.14 (2), 0.28 (3), 0.42 (4), 0.55 (5), 0.69 (6), 0.83 (7), 0.97 (8), 1.11 (9), 1.38 (10), 1.66 (11), 2.22 (12), 2.77 (13), 3.32 (14), 3.88 (15), 4.99 (16)); Acetonitrile-$d_3$, 298 K, 500 MHz ($^1$H) or 470 MHz ($^{19}$F). During the $^1$H NMR titration (a) the signal corresponding to $Z$-**1**H$^{2+}$ (4.25 ppm, red arrow) disappears completely after the first addition. The peaks in the first and last $^{19}$F NMR spectra (b) refer to the complexation state of the ammonium station: complexed station resonance at –63.0 ppm, free station resonance at –63.3 ppm.

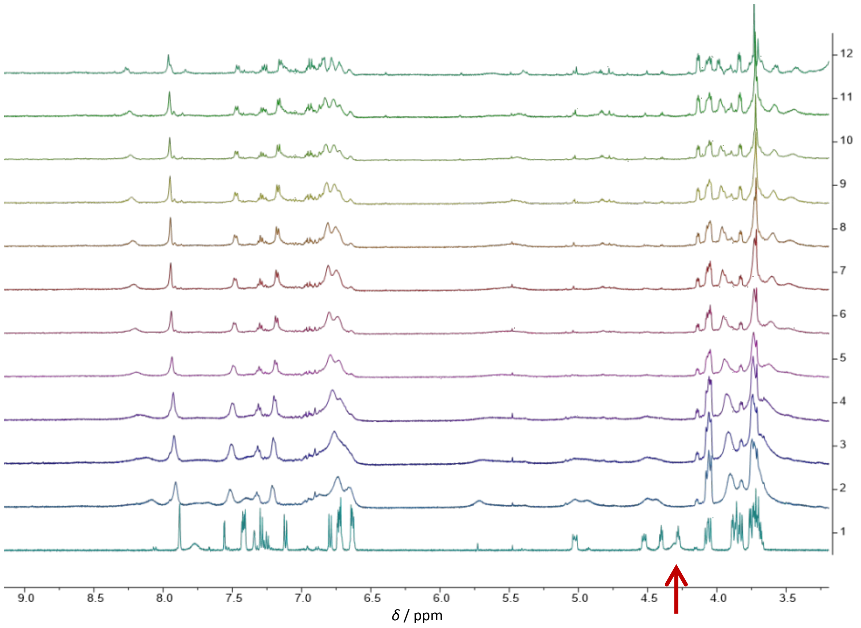


**Figure S48.** $^{1}$H NMR spectral changes of *Z*-**1**H$^{2+}$ upon titration with base **B4** under continuous irradiation (*n*(**B4**/*Z*-**1**H$^{2+}$) = 0.00 (1), 12.0 (2), 23.8 (3), 35.7 (4), 59.6 (5), 83.4 (6), 107.2 (7), 131.0 (8), 166.8 (9), 202.5 (10), 238.2 (11), 476.5 (12)); Acetonitrile-$d_3$, 298 K, 500 MHz. During the titration the signal corresponding to *Z*-**1**H$^{2+}$ (4.25 ppm, red arrow) disappears completely after the first addition.

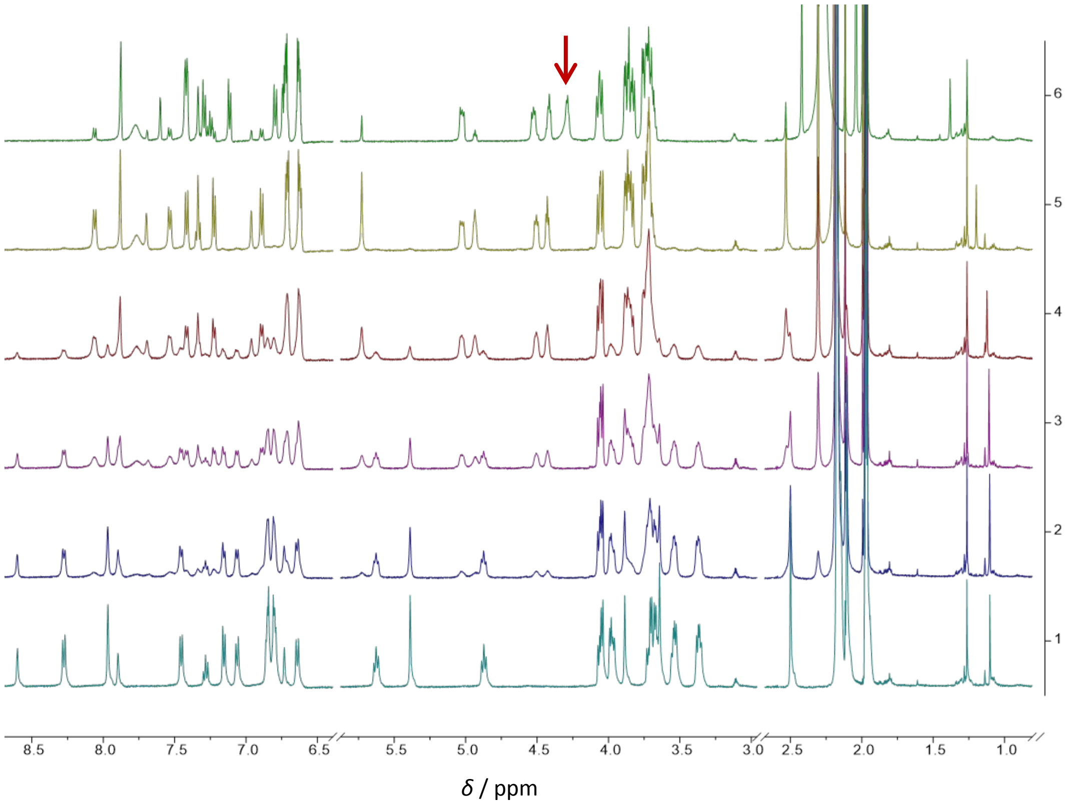


**Figure S491.** $^{1}H$ NMR spectral changes of $E$-**1**$^{+}$ upon titration with trifluoroacetic acid under continuous irradiation ($n$(TFA/$E$-**1**$^{+}$) = 0.00 (1), 0.17 (2), 0.35 (3), 0.52 (4), 0.70 (5), 1.00 (6)); Acetonitrile-$d_3$, 298 K, 500 MHz). At the end of the titration (spectrum 6) the signal corresponding to $Z$-**1**H$^{2+}$ (4.25 ppm, red arrow) becomes evident.

a)

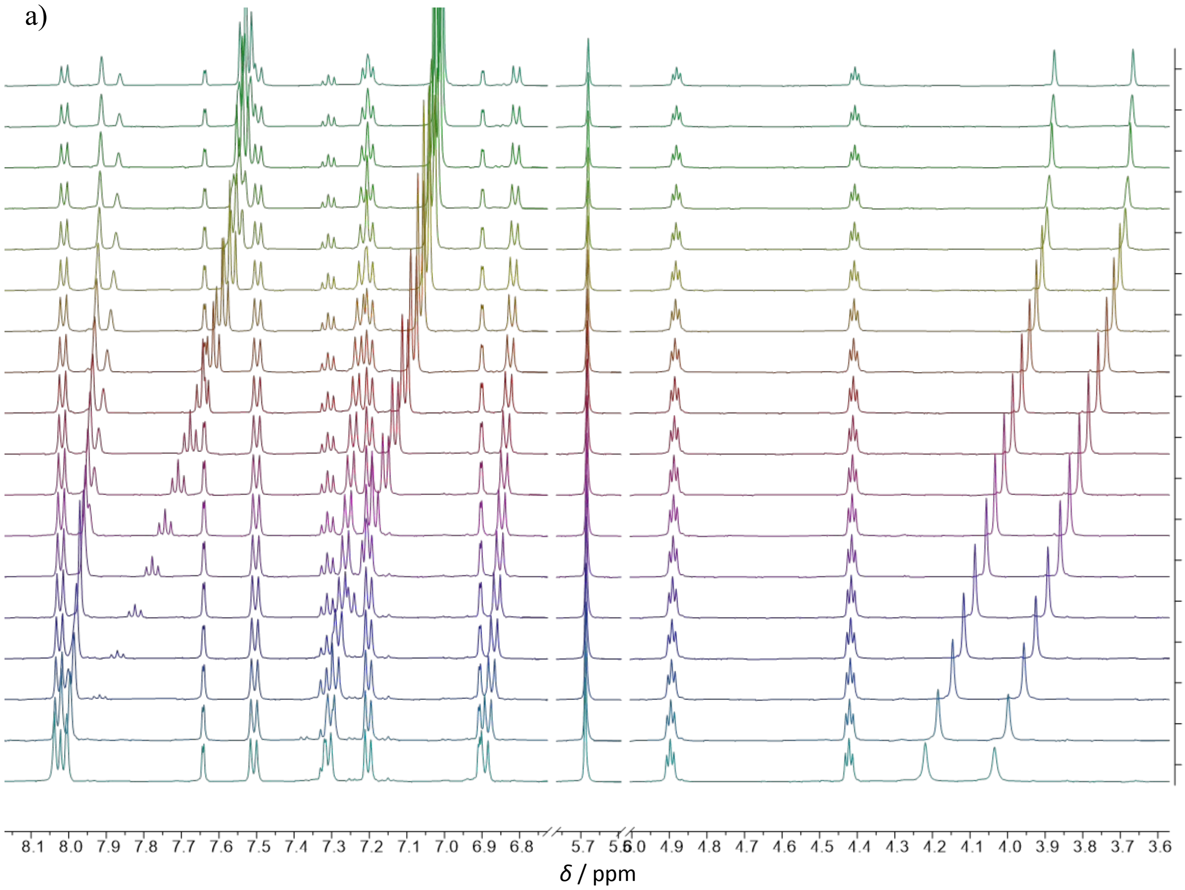


b)

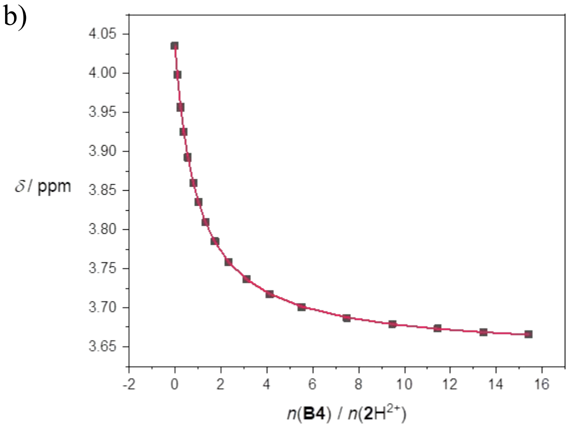


**Figure S50.** a) $^{1}$H NMR spectral changes of $E$-**2**H$^{2+}$ upon titration with base **B5** ($n$(**B5**/ $E$-**2**H$^{2+}$) = 0.00 (1), 0.10 (2), 0.24 (3), 0.38 (4), 0.55 (5), 0.79 (6), 1.03 (7), 1.35 (8), 1.74 (9), 2.34 (10), 3.13 (11), 4.12 (12), 5.51 (13), 7.49 (14), 9.47 (15), 11.46 (16), 13.44 (17), 15.42 (18)) b) titration curve obtained by fitting of the peak shifts in $^{1}$H NMR spectra ( ■ experimental data, – calculated data); Acetonitrile-$d_3$, 298 K, 500 MHz.

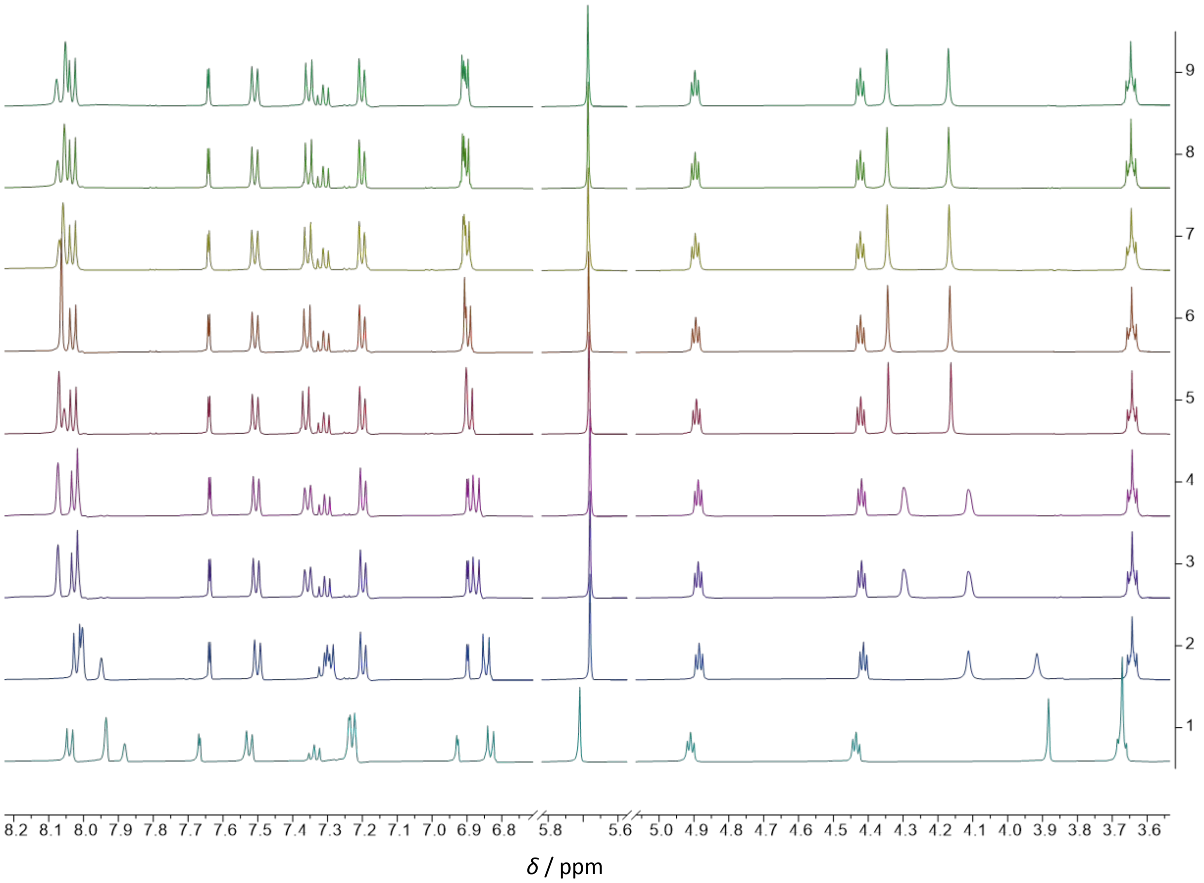


**Figure S51.** $^{1}$H NMR spectral changes of *E*-$\mathbf{2}^{+}$ upon titration with trifluoroacetic acid; Acetonitrile-$d_3$, 298 K, 500 MHz.

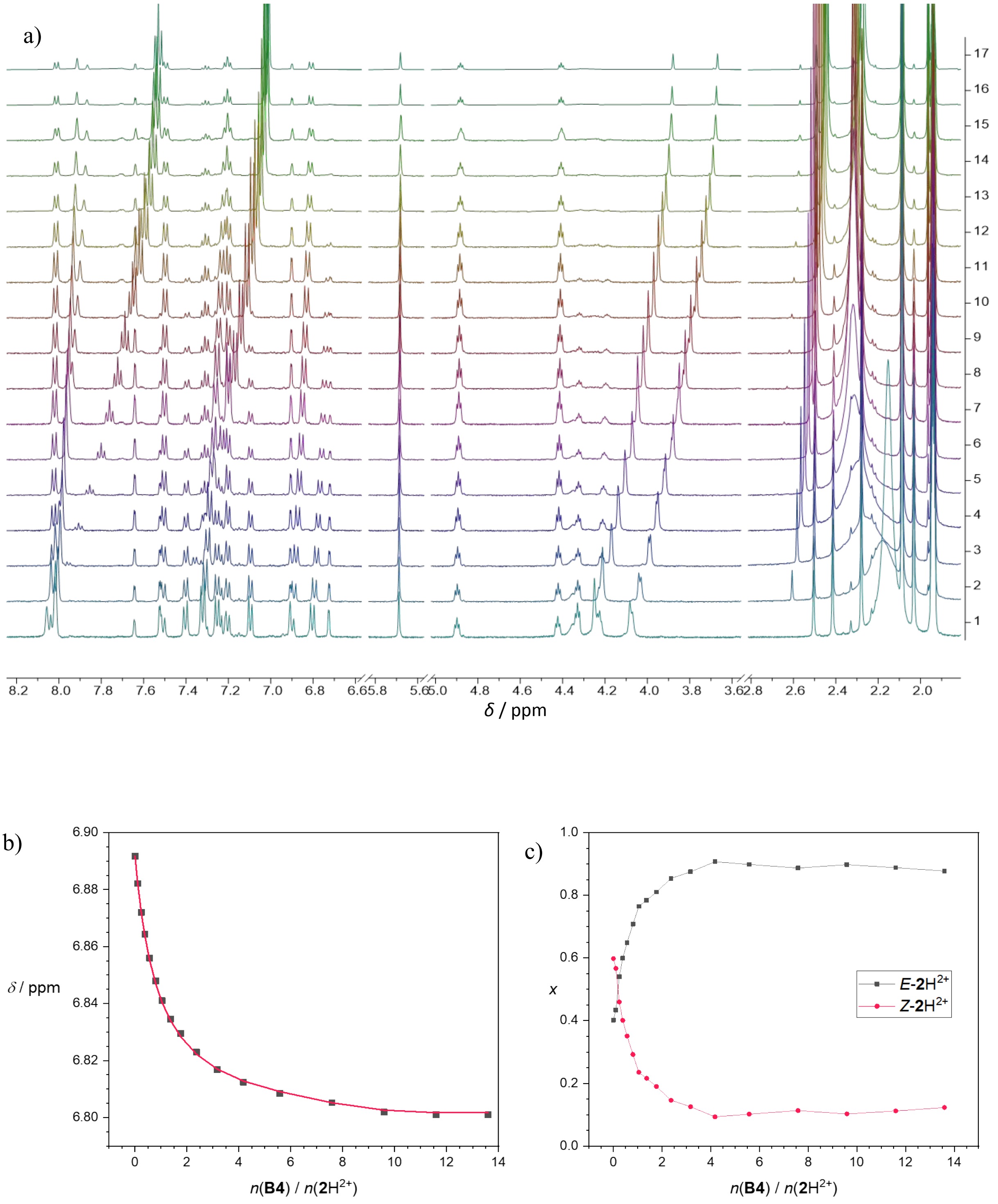


**Figure S52.** a) $^{1}$H NMR spectral changes of *Z*-**2**$H^{2+}$ upon titration with base **B5** under continuous irradiation (*n*(**B5**/*Z*-**2**$H^{2+}$) = 0.00 (1), 0.10 (2), 0.24 (3), 0.38 (4), 0.56 (5), 0.80 (6), 1.04 (7), 1.36 (8), 1.77 (9), 2.37 (10), 3.17 (11), 4.17 (12), 5.58 (13), 7.58 (14), 9.59 (15), 11.59 (16), 13.60 (17)) b) titration curve obtained by fitting of the peak shifts in $^{1}$H NMR spectra ( ■ experimental data, – calculated data). c) changes in the molar ratio of *E*- and *Z*- species (*E*-**2**$H^{2+}$ + *E*-**2**$^{+}$ *vs*. *Z*-**2**$H^{2+}$ + *Z*-**2**$^{+}$) during the titration; Acetonitrile-$d_3$, 298 K, 500 MHz.

# 4. Spectrophotometric data

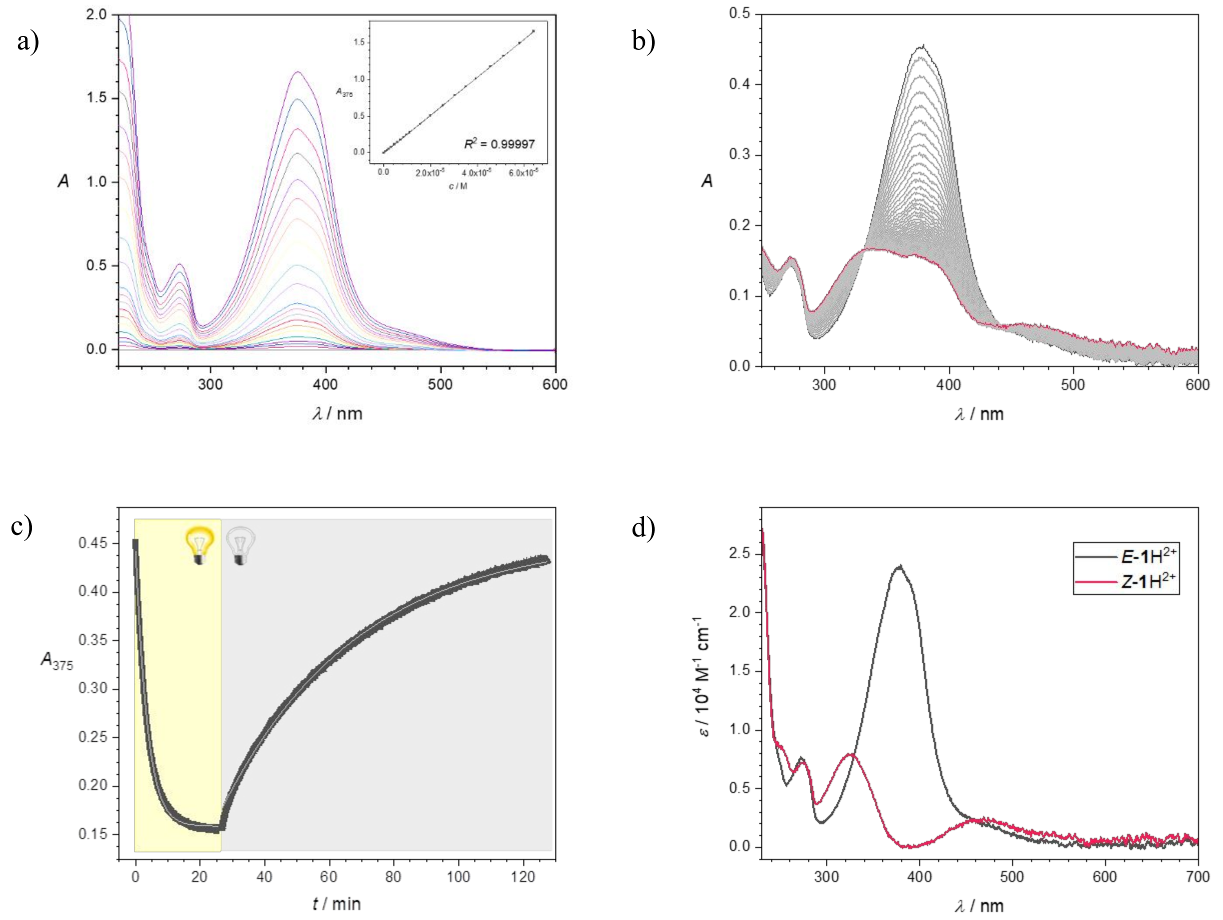


**Figure S53.** a) Changes in absorption spectra of $E$-**1**$H^{2+}$ in Acetonitrile at 298 K upon dilution; inset: linear dependence of absorbance at $\lambda_{max}$ = 375 nm on concentration of $E$-**1**$H^{2+}$; b) Absorption spectrum of $E$-**1**$H^{2+}$ in Acetonitrile at 298 K, $c = 1.9 \times 10^{-5}$ mol dm$^{-3}$ (black line) and spectral changes upon irradiation with 365 nm light at room temperature ($E \rightarrow Z$ photoisomerization, gray lines) until reaching the photostationary state (red line); c) Changes in the absorbance at $\lambda_{max}$ = 375 nm during isomerization (upon irradiation with 365 nm light) and back-isomerization (in the dark) ( ■ experimental data, – calculated data); d) Calculated molar absorption spectra of $E$-**1**$H^{2+}$ (black line) and $Z$-**1**$H^{2+}$ (red line).

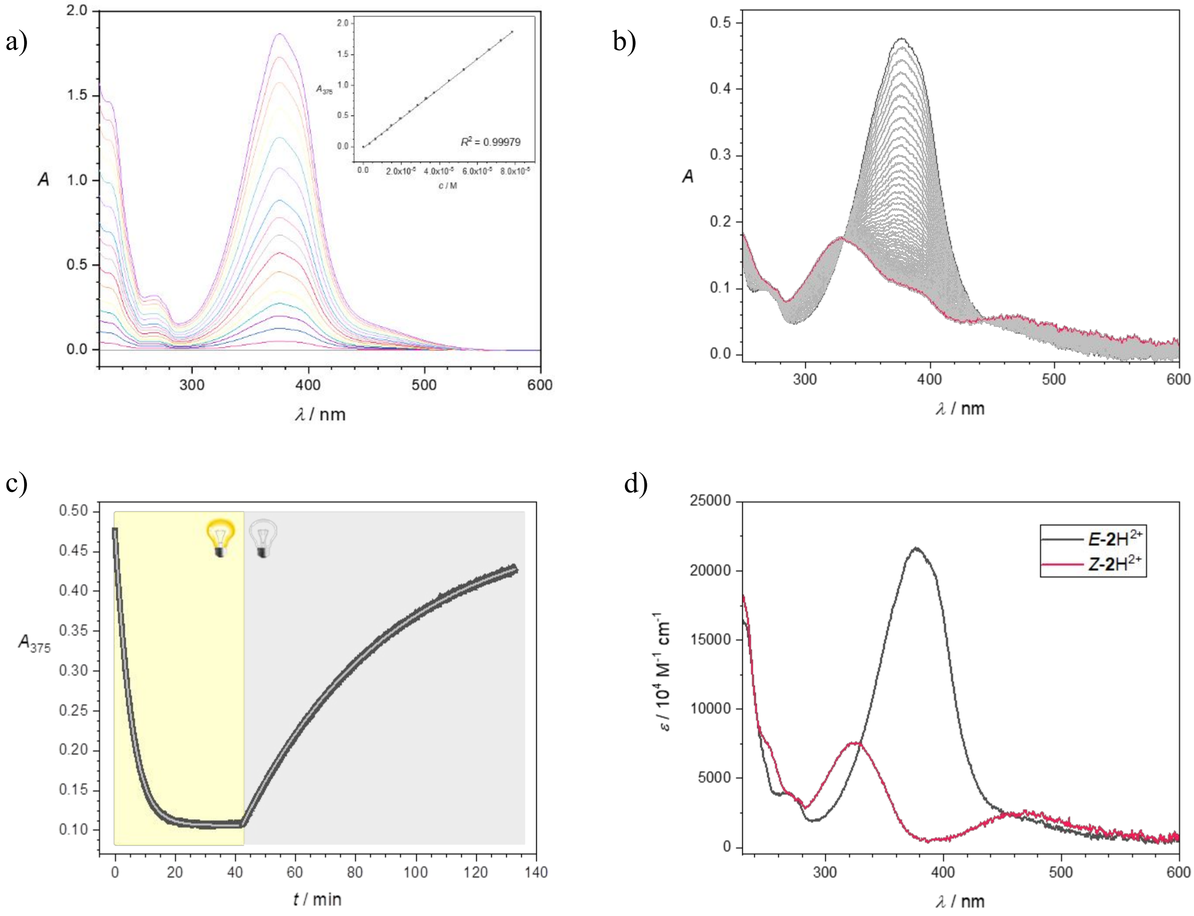


**Figure S54.** a) Changes in absorption spectra of *E*-**2**$H^{2+}$ in Acetonitrile at 298 K upon dilution; inset: linear dependence of absorbance at $\lambda_{max}$ = 375 nm on concentration of *E*-**2**$H^{2+}$; b) Absorption spectrum of *E*-**2**$H^{2+}$ in Acetonitrile at 298 K, $c = 1.6 \times 10^{-5}$ mol dm$^{-3}$ (black line) and spectral changes upon irradiation with 365 nm light at room temperature ($E \rightarrow Z$ photoisomerization, gray lines) until reaching the photostationary state (red line); c) Changes in the absorbance at $\lambda_{max}$ = 375 nm during isomerization (upon irradiation with 365 nm light) and back-isomerization (in the dark) ( ■ experimental data, – calculated data); d) Calculated molar absorption spectra of *E*-**2**$H^{2+}$ (black line) and *Z*-**2**$H^{2+}$ (red line).

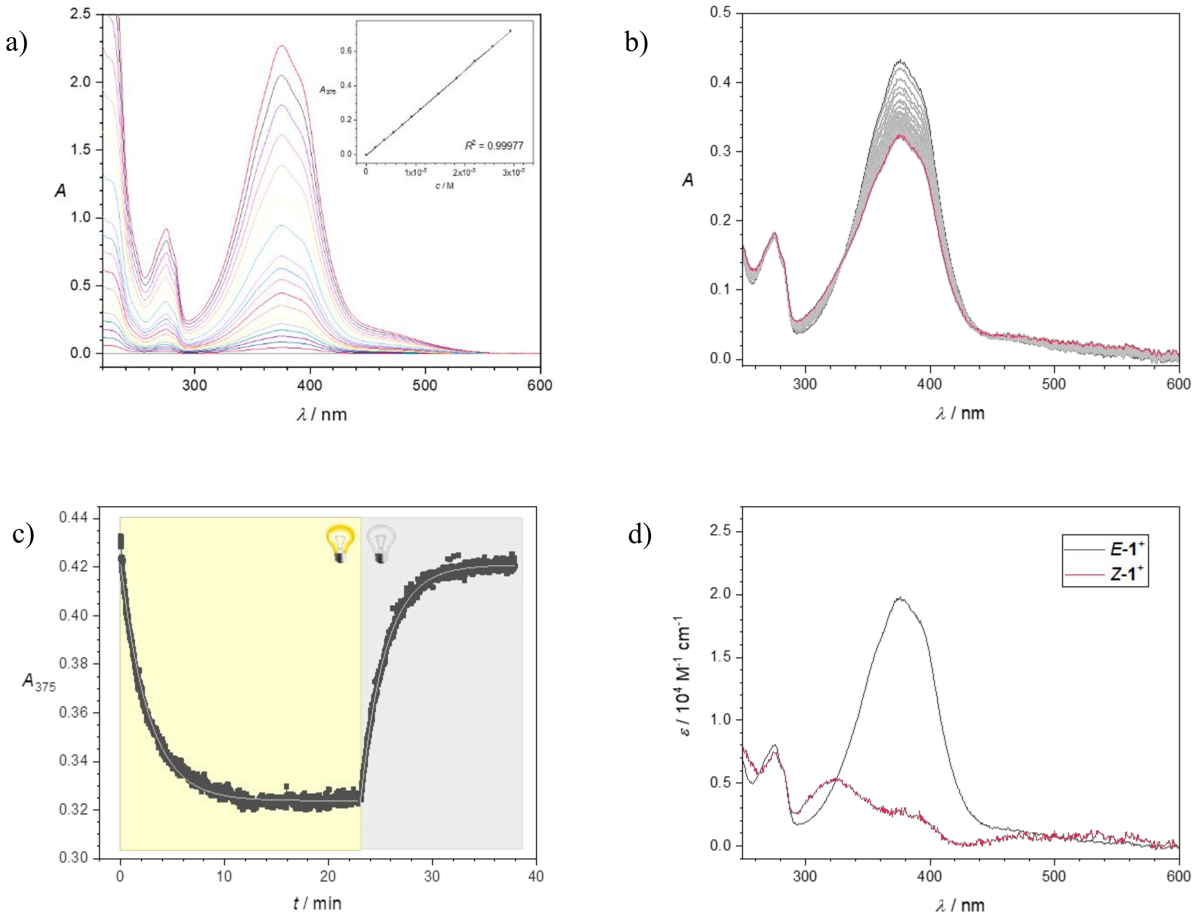


**Figure S55.** a) Changes in absorption spectra of $E$-**1**$^+$ in Acetonitrile at 298 K upon dilution; inset: linear dependence of absorbance at $\lambda_{max} = 375$ nm on concentration of $E$-**1**$^+$; b) Absorption spectrum of $E$-**1**$^+$ in Acetonitrile at 298 K, $c = 2.0 \times 10^{-5}$ mol dm$^{-3}$ (black line) and spectral changes upon irradiation with 365 nm light at room temperature ($E \rightarrow Z$ photoisomerization, gray lines) until reaching the photostationary state (red line); c) Changes in the absorbance at $\lambda_{max} = 375$ nm during isomerization (upon irradiation with 365 nm light) and back-isomerization (in the dark) ( ■ experimental data, – calculated data); d) Calculated molar absorption spectra of $E$-**1**$^+$ (black line) and $Z$-**1**$^+$ (red line).

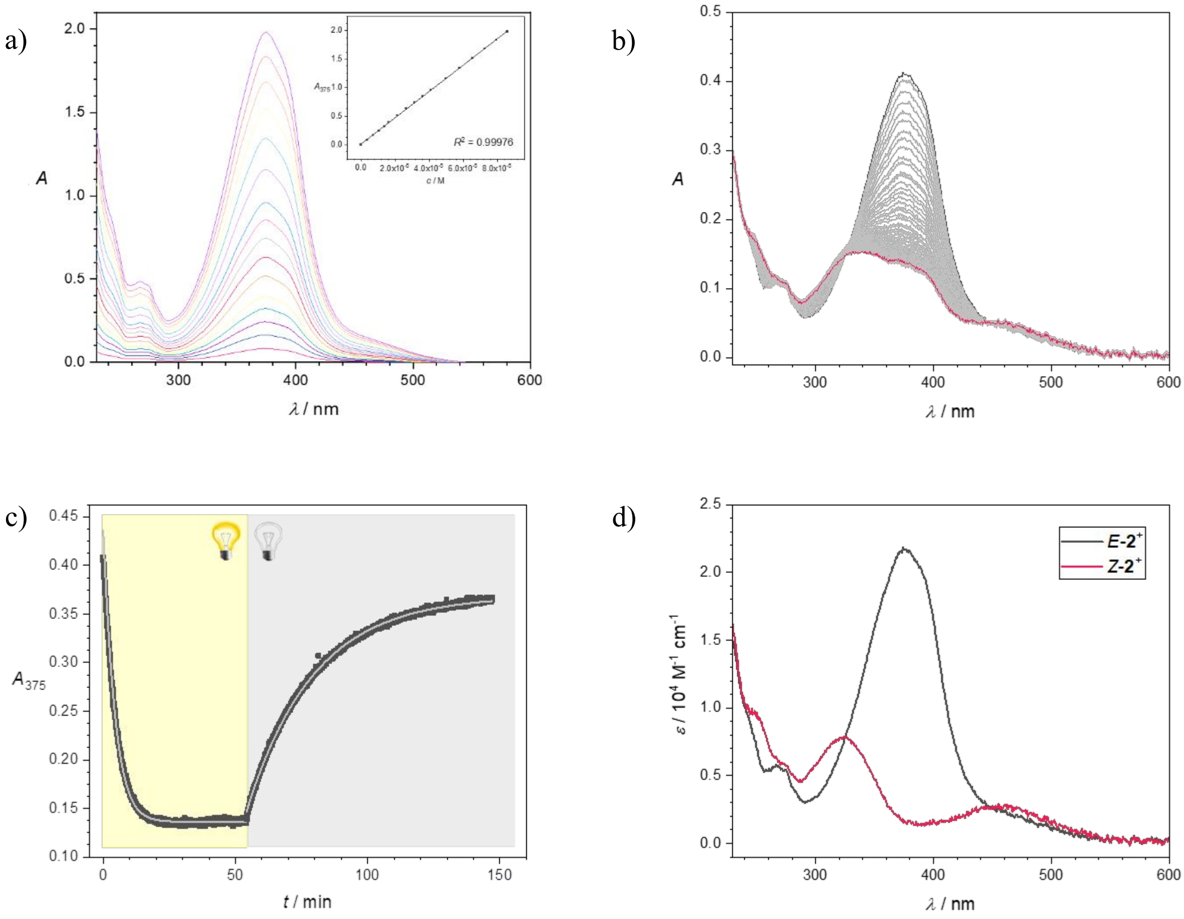


**Figure S56.** a) Changes in absorption spectra of *E*-**2**$^+$ in Acetonitrile at 298 K upon dilution; inset: linear dependence of absorbance at $\lambda_{max} = 375$ nm on concentration of *E*-**2**$^+$; b) Absorption spectrum of *E*-**2**$^+$ in Acetonitrile at 298 K, $c = 1.9 \times 10^{-5}$ mol dm$^{-3}$ (black line) and spectral changes upon irradiation with 365 nm light at room temperature ($E \rightarrow Z$ photoisomerization, gray lines) until reaching the photostationary state (red line); c) Changes in the absorbance at $\lambda_{max} = 375$ nm during isomerization (upon irradiation with 365 nm light) and back-isomerization (in the dark) ( ■ experimental data, – calculated data); d) Calculated molar absorption spectra of *E*-**2**$^+$ (black line) and *Z*-**2**$^+$ (red line).

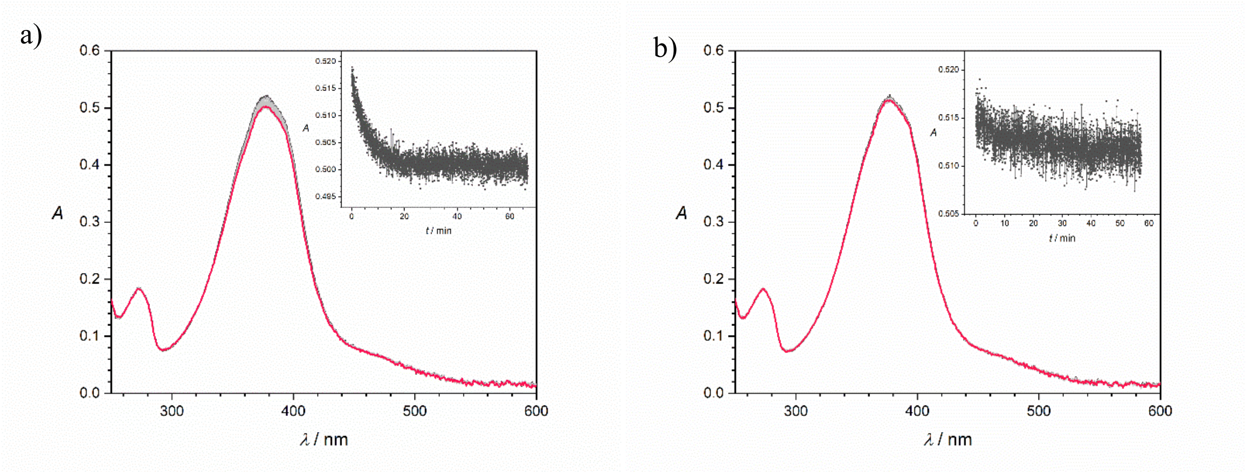


**Figure S57.** a) Absorption spectrum of $E$-**1**$H^{2+}$ in Acetonitrile at 298 K, $c = 1.8 \times 10^{-5}$ mol dm$^{-3}$ (black line) and spectral changes upon irradiation with 313 nm light at room temperature ($E \rightarrow Z$ photoisomerization) until reaching the photostationary state (red line). Inset: changes in the absorbance at $\lambda_{max} = 375$ nm during isomerization. b) Absorption spectrum of $E$-**1**$H^{2+}$ in Acetonitrile at 298 K, $c = 1.8 \times 10^{-5}$ mol dm$^{-3}$ (black line) and spectral changes upon irradiation with 546 nm light at room temperature ($E \rightarrow Z$ photoisomerization) until reaching the photostationary state (red line). Inset: changes in the absorbance at $\lambda_{max} = 375$ nm during isomerization.

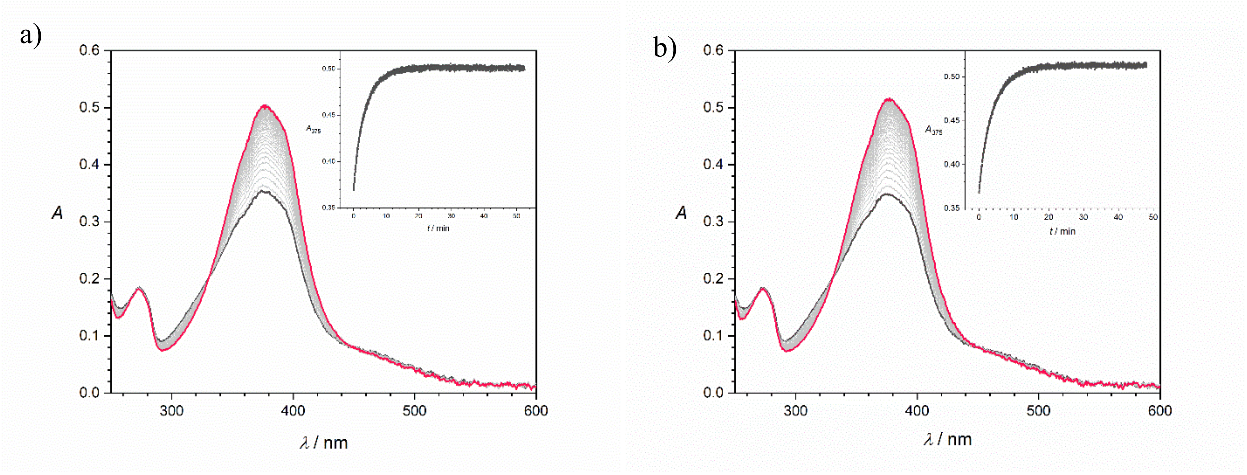


**Figure S58.** a) Absorption spectrum of the photostationary state obtained by irradiation of $E$-**1**$H^{2+}$ in Acetonitrile at 298 K, $c = 1.8 \times 10^{-5}$ mol dm$^{-3}$ (black line) and spectral changes upon irradiation with 313 nm light at room temperature ($Z \rightarrow E$ photo-backisomerization) until reaching the photostationary state (red line). Inset: changes in the absorbance at $\lambda_{max} = 375$ nm during isomerization. b) Absorption spectrum of the photostationary state obtained by irradiation of $E$-**1**$H^{2+}$ in Acetonitrile at 298 K, $c = 1.8 \times 10^{-5}$ mol dm$^{-3}$ (black line) and spectral changes upon irradiation with 546 nm light at room temperature ($Z \rightarrow E$ photo-backisomerization) until reaching the photostationary state (red line). Inset: changes in the absorbance at $\lambda_{max} = 375$ nm during isomerization.

**Table S1.** Rate constants obtained by fitting using a two contributions kinetic model for the back-isomerization in the dark after irradiation of $E$-1H$^{2+}$ and $E$-1$^{+}$ mixtures with different molar ratios in Acetonitrile at 298 K to the photostationary state. The errors reported for $k_A$ and $k_B$ are relative to the fitting of the experimental data.

| $E$-**1**$^{+}$ / $E$-**1**H$^{2+}$ | $k_A$ / s$^{-1}$ * | $k_B$ / s$^{-1}$ |
|---|---|---|
| 0.02 | $(5.31 \pm 0.20) \times 10^{-3}$ | $(3.03 \pm 0.02) \times 10^{-4}$ |
| 0.06 | $(6.85 \pm 0.07) \times 10^{-3}$ | $(5.20 \pm 0.06) \times 10^{-4}$ |
| 0.19 | $(7.16 \pm 0.20) \times 10^{-3}$ | $(2.84 \pm 0.10) \times 10^{-4}$ |
| 0.27 | $(1.10 \pm 0.01) \times 10^{-2}$ | $(1.43 \pm 0.02) \times 10^{-4}$ |
| 0.43 | $(1.17 \pm 0.01) \times 10^{-2}$ | $(5.20 \pm 0.02) \times 10^{-4}$ |
| 0.49 | $(1.47 \pm 0.02) \times 10^{-2}$ | $(2.46 \pm 0.40) \times 10^{-4}$ |
| $k_\Delta$($Z$-**1**H$^{2+}$) = $(3.97 \pm 0.06) \times 10^{-4}$ s$^{-1}$ | | |
| $k_\Delta$($Z$-**1**$^{+}$) = $(6.96 \pm 0.02) \times 10^{-3}$ s$^{-1}$ | | |

* $k_A$ is an apparent pseudo-first order rate constant

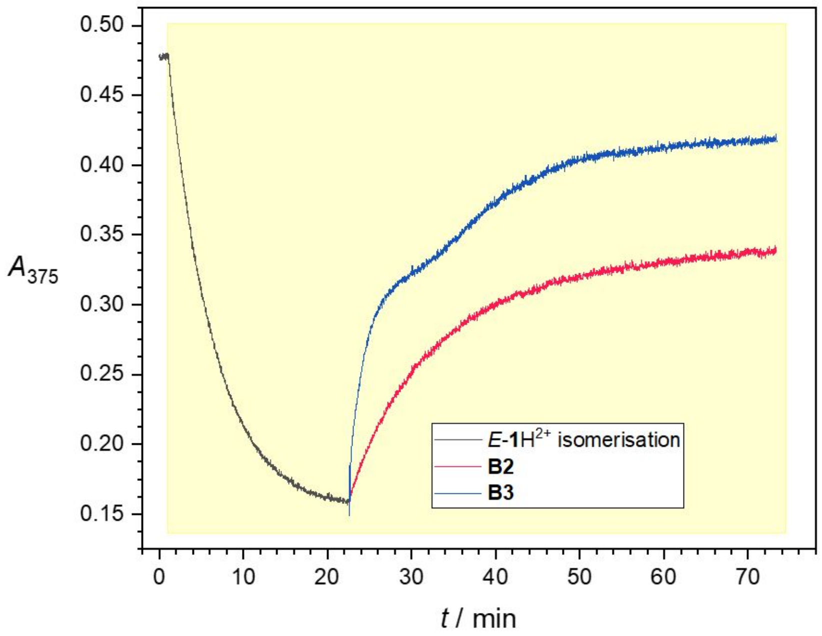


**Figure S59.** Changes in absorption at 375 nm for a *E*-**1**$H^{2+}$ solution upon irradiation to the photostationary state (black curve) and subsequent addition of bases **B2** (red curve) or **B3** (blue line) under continuous light irradiation; $c$(*E*-**1**$H^{2+}$) = 1.8 × $10^{-5}$ M, Acetonitrile, 298 K.

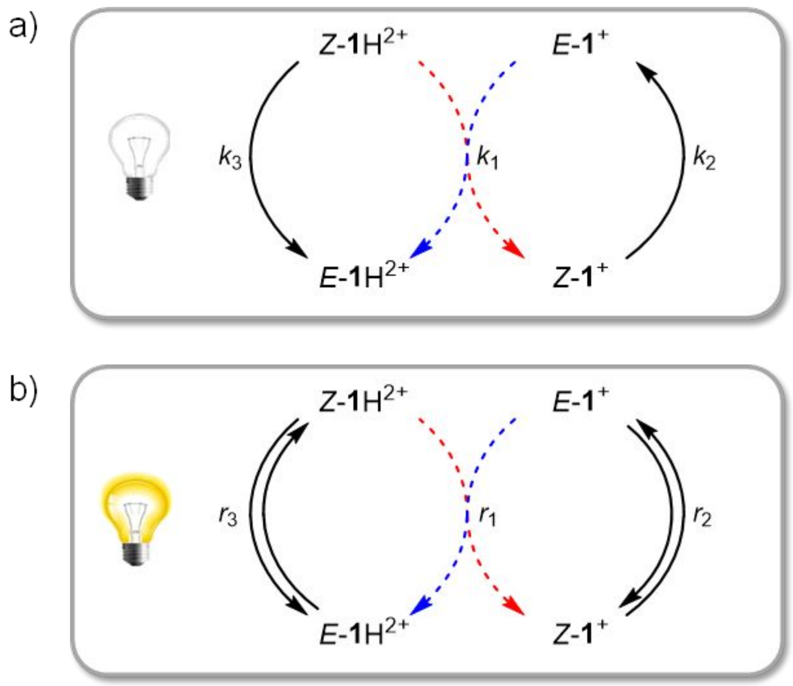


**Figure S60.** Reaction networks including the deprotonation of *Z*-**1**$H^{2+}$ by *E*-**1**$^{+}$ in the dark (a) or under continuous irradiation (b).

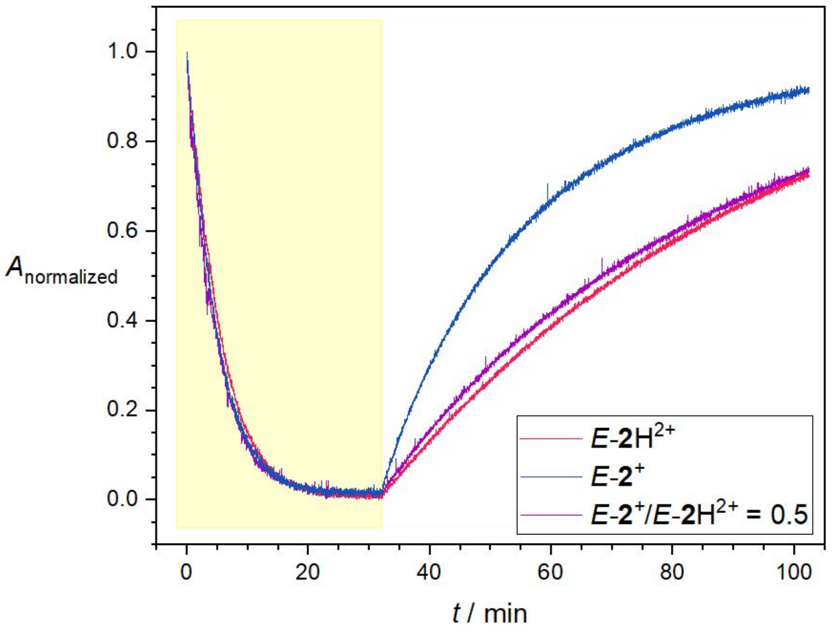


**Figure S61.** Changes in absorption at 375 nm upon addition of $E$-**2**$^+$ to $Z$-**2**H$^{2+}$. Acetonitrile, 298 K.

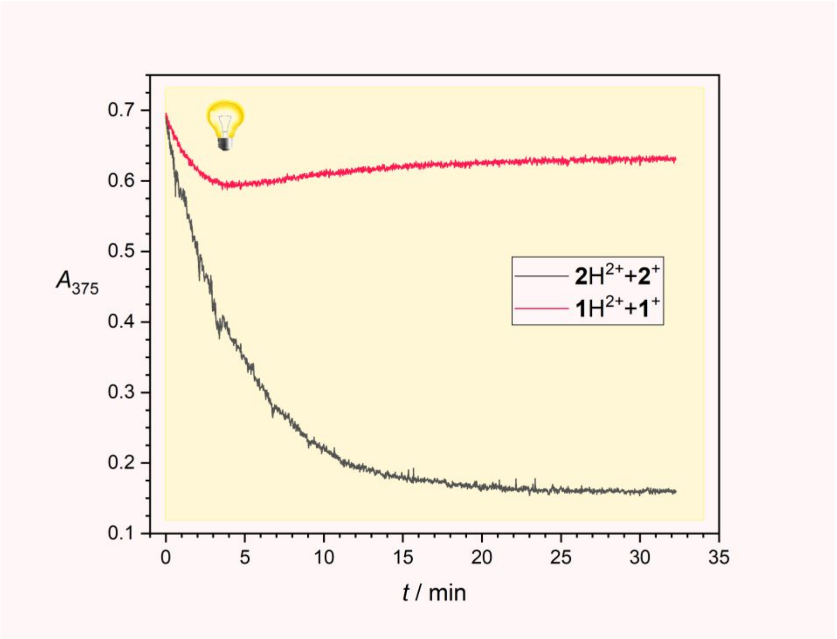


**Figure S62.** Changes in absorption at 375 nm for a solution containing $E$-**1**H$^{2+}$ and $E$-**1**$^+$ (red trace, $E$-**1**$^+$/$E$-**1**H$^{2+}$ = 0.49) and a solution containing $E$-**2**H$^{2+}$ and $E$-**2**$^+$ (black trace, $E$-**2**$^+$/$E$-**2**H$^{2+}$ = 0.49) upon irradiation at 365 nm. Conditions: $c_0$($E$-**1**$^+$) = $c_0$($E$-**1**H$^{2+}$) = $c_0$($E$-**2**$^+$) = $c_0$($E$-**2**H$^{2+}$) = 18 µM, acetonitrile, 298 K.

## References

[1] Montalti, M.; Credi, A.; Prodi, L.; Gandolfi, M. T. Handbook of Photochemistry, 3rd Edition, CRC Press, Boca Raton, 2006.

[2] Tshepelevitsh, S.; Kütt, A.; Lõkov, M.; Kaljurand, I.; Saame, J.; Heering, A.; Plieger, P. G.; Vianello, R.; Leito, I. On the Basicity of Organic Bases in Different Media. *Eur J. Org. Chem.* **2019**, *40*, 6735-6748.

[3] Gans, P.; Sabatini, A.; Vacca, A. Investigation of equilibria in solution. Determination of equilibrium constants with the HYPERQUAD suite of programs. *Talanta* **1996**, *43*, 1739-1753.

[4] Mallick, D.; Nandi, A.; Datta, S.; Sarker, K. K.; Mondal, T. K.; Sinha, C. The synthesis, structure and photochromism of mercury(II)-iodide complexes of 1-$C_nH_{2n+1}$-2-(arylazo)imidazoles ($n$ = 4, 6, 8). *Polyhedron* **2012**, *31*, 506-514.
